# Isothermal self-assembly of multicomponent and evolutive DNA nanostructures

**DOI:** 10.1038/s41565-023-01468-2

**Published:** 2023-07-31

**Authors:** Caroline Rossi-Gendron, Farah El Fakih, Laura Bourdon, Koyomi Nakazawa, Julie Finkel, Nicolas Triomphe, Léa Chocron, Masayuki Endo, Hiroshi Sugiyama, Gaëtan Bellot, Mathieu Morel, Sergii Rudiuk, Damien Baigl

**Affiliations:** 1 PASTEUR, Department of Chemistry, École Normale Supérieure, PSL University, Sorbonne Université, CNRS, Paris, France; 2 Centre de Biologie Structurale, Université Montpellier, CNRS, Inserm, Montpellier, France; 3 Université Grenoble Alpes, CEA, Leti,, Grenoble, France; 4 Organization for Research and Development of Innovative Science and Technology, Kansai University, Suita, Japan; 5 Department of Chemistry, Graduate School of Science, Kyoto University, Kitashirakawa-Oiwakecho, Kyoto, Japan; 6 Institute for Integrated Cell-Material Sciences (WPI-iCeMS), Kyoto University, Yoshida-Ushinomaecho, Kyoto, Japan

**Keywords:** DNA nanostructures, DNA nanomachines, Supramolecular chemistry, Molecular self-assembly

## Abstract

Thermal annealing is usually needed to direct the assembly of multiple complementary DNA strands into desired entities. We show that, with a magnesium-free buffer containing NaCl, complex cocktails of DNA strands and proteins can self-assemble isothermally, at room or physiological temperature, into user-defined nanostructures, such as DNA origamis, single-stranded tile assemblies and nanogrids. In situ, time-resolved observation reveals that this self-assembly is thermodynamically controlled, proceeds through multiple folding pathways and leads to highly reconfigurable nanostructures. It allows a given system to self-select its most stable shape in a large pool of competitive DNA strands. Strikingly, upon the appearance of a new energy minimum, DNA origamis isothermally shift from one initially stable shape to a radically different one, by massive exchange of their constitutive staple strands. This method expands the repertoire of shapes and functions attainable by isothermal self-assembly and creates a basis for adaptive nanomachines and nanostructure discovery by evolution.

## Abstract (web summary)

In contrast to conventional thermal annealing approaches, the authors report on the self-assembly of complex mixtures of DNA at room or physiological temperature for generating user-defined programmable nanostructures capable of shape selection and transformation.

## Main

Self-assembly is a process whereby naturally occurring or rationally designed entities embed the necessary information to spontaneously interact and self-organize into functional superstructures of interest^1^. Synthetic self-assembled materials are usually equilibrium structures resulting from the spatial organization of a repeating single component into a stable supramolecular assembly, such as micelles or colloidal crystals, with a prescribed set of useful properties. They usually have, however, limited intrinsic reconfigurability, and producing the desired structures with more than a few different components is still highly challenging. In nature, self-assembled systems are key elements of living entities and, contrary to their synthetic counterparts, are usually out-of-equilibrium and multicomponent structures capable of dynamic behaviours such as reconfigurability, adaptation or evolution. Obtaining multiple-component self-assembling synthetic materials capable of such lifelike functions would arguably expand the applicability, versatility and sustainability of human-made smart materials. By exploiting the sequence-dependent base-pairing principle between synthetic DNA single strands, structural DNA nanotechnology^2^ appears as a particularly powerful approach to address this challenge because it makes it possible to programme the assembly of hundreds of different components into elaborate superstructures of desired shape^3,4^, size^5,6^ and site-specific functionality^3,7^, potentially at a large scale^8^, leading to a wide range of applications^9,10^. The assembly of such multicomponent structures is, however, usually directed by a thermal annealing process in which the DNA mixture is first heated above its melting temperature before being slowly cooled down to avoid kinetic traps and ensure proper sequence-specific DNA hybridization^11^. Such a thermal treatment hinders any possibility for spontaneous nanostructure formation or evolution under fixed environmental conditions. This also leads to energetically highly stabilized structures for which dynamic actuation and transformation is possible^12–16^, but typically relies on supplemental action on preformed objects^17^, using, for instance, linker strand hybridization^18–20^, strand displacement^21^, supramolecular interactions^22^, enzymatic editing^23^ or photoactuation strategies^24,25^. Thermal annealing thus produces structures that can be actuated once they are formed but are not intrinsically evolutive. It also constitutes an obstacle to one-pot, in situ functionalization with temperature-sensitive entities such as proteins. From a more fundamental viewpoint, it raises the question whether such sophisticated, multicomponent DNA nanostructures could self-assemble flawlessly at constant temperature. A few isothermal protocols have been described in the literature but each has limitations such as involving denaturing agents^26–28^, preformed assemblies^29^, specific sequence designs^30^ or specific working temperatures^31,32^. Most of these isothermal approaches were also dedicated to a specific DNA assembly method (for example, DNA origami^3^ or single-stranded tile (SST) assembly^4^), and lacked a generic character that could offer increased applicability and better understanding of DNA isothermal self-assembly. In this work, we show that the major methods of structural DNA nanotechnology, including DNA origamis, DNA nanogrids^33^ and SST assemblies, can be operated by the same generic isothermal DNA self-assembly principle, leading to a breadth of user-defined elaborate DNA nanostructures that can be spontaneously formed at room or body temperature, keeping intrinsic reconfigurability and offering the capability of complete shape transformation. By using a magnesium-free buffer containing suitable amounts of NaCl to electrostatically stabilize the forming structures while allowing enough reconfigurability, we established the range of temperatures and salt concentrations at which the self-assembly was operational and applied it to the isothermal production of various nanostructure designs and shapes, and their concomitant functionalization by proteins. We explored a range of structural complexity involving designs with (origamis) or without (SSTs, nanogrids) scaffolds, with a finite size (origamis, SSTs) or self-repeating (nanogrids), with bidimensional (2D) or tridimensional (3D) morphologies. By in situ real-time atomic force microscopy (AFM), we revealed the multiplicity of folding pathways in self-assembling 2D origamis. Finally, we performed a series of experiments demonstrating some unique characteristics of this thermodynamically controlled isothermal assembly method, ranging from shape selection in a highly multicomponent mixture of competing DNA strands to complete shape transformation by the massive exchange of hundreds of different strand components.

## Highly versatile and multicomponent self-assembly in NaCl

**Fig. 1:**
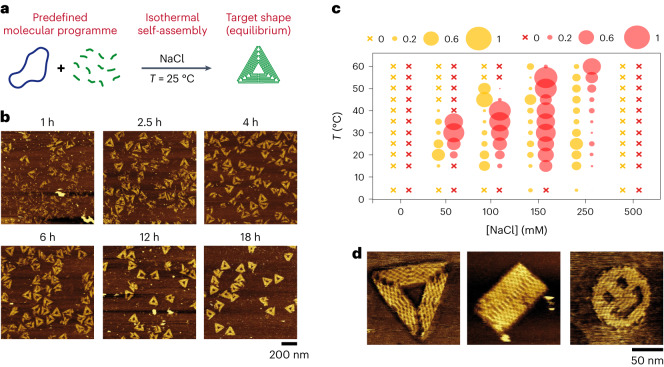
Isothermal self-assembly of user-defined DNA origamis in a magnesium-free NaCl buffer. **a**, An origami mix (M13 scaffold plus a 40× excess of desired staples) can spontaneously self-assemble at constant temperature into the target equilibrium shape (for example, a triangle) in TANa buffer. **b**, AFM observation of the isothermal origami formation at 25 °C in TANa ([NaCl] = 100 mM), for a set of staples coding for sharp triangles, as a function of incubation time. **c**, Fraction (bubble size) of partially folded (yellow) and fully folded (red) origamis after 24 h of isothermal self-assembly with a set of staples coding for sharp triangles, for various incubation temperatures (*T*) and NaCl concentrations. A cross symbol indicates a fraction of 0. For the sake of readability, the remaining fraction, which corresponds to non- or misfolded origamis, is not plotted in this graph but is displayed in Supplementary Fig. 3. All images used for these analyses are available in a citable public repository (doi: 10.5281/zenodo.7998757) and can be accessed directly at the following link: https://zenodo.org/record/7998757. The number *n* of analysed objects for each condition is given in Supplementary Table 1. **d**, Representative close-up AFM images of origamis obtained by isothermal assembly in TANa ([NaCl] = 100 mM) at 25 °C for staples coding for sharp triangles (left), tall rectangles (middle) and smileys (right). For all experiments: [M13] = 1 nM; each staple concentration is 40 nM; no staple purification was performed before AFM imaging. Source data

**Fig. 2:**
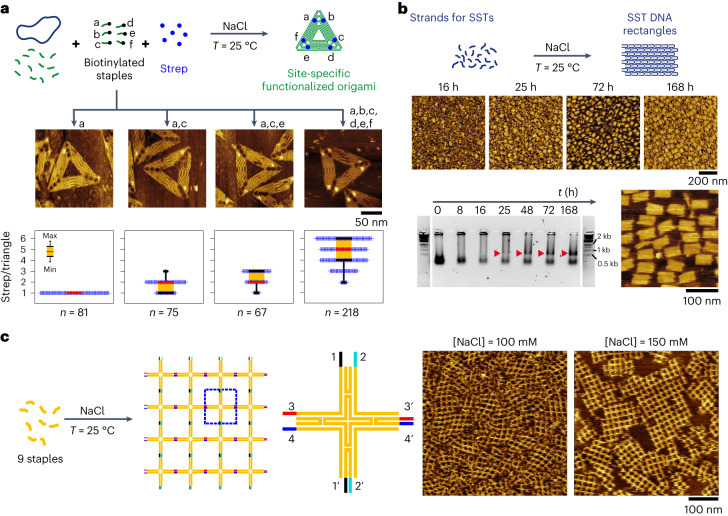
Expanded applicability of the isothermal self-assembly: protein functionalization, tile assembly and nanogrid formation at 25 °C in TANa buffer. **a**, Top: self-assembly of an origami mix with staples coding for sharp triangles, including biotinylated staples at specific positions a–f, in the presence of 2 µM streptavidin (Strep). Middle: representative AFM images of the obtained origamis after 24 h of assembly in TANa ([NaCl] = 100 mM), as a function of the biotinylated staples used in the mix. Larger-scale images are given in Supplementary Fig. 16. Bottom: fraction (%) of streptavidin detected on origamis functionalized with 1 to 6 streptavidin proteins. The box plots display a box (orange) ranging from the first to third quartiles (black bars), with extrema indicated by shorter black bars and the median shown as a red bar. Each of the *n* counted streptavidins is represented by a cross. All images used for these analyses are available in a citable public repository (doi: 10.5281/zenodo.7998757) and can be accessed directly at the following link: https://zenodo.org/record/7998757. [M13] = 1 nM; each staple concentration is 40 nM. **b**, Top: self-assembly of SSTs into an R4 rectangle with 100 mM NaCl. Middle: AFM images as a function of self-assembly time. Bottom left: electrophoresis gel of the self-assembling mixture as a function of time, with ladders shown on extreme left and right. Red arrowheads indicate the bands of fully formed R4 rectangles. Bottom right, AFM image of R4 rectangle after 63 h of self-assembly and purification by gel electrophoresis. **c**, Self-assembly of nine oligonuclotides (1 µM each) forming extended DNA nanogrids with representative AFM images after 24 h assembly with [NaCl] = 100 mM (left) or 150 mM (right). Larger-scale images are given in Supplementary Fig. 17. Source data

First, we simply assembled a DNA origami mix (M13 scaffold plus a 40× excess of staples coding for sharp triangles), without any thermal pretreatment, and let it incubate at 25 °C for several hours (Fig. 1a). When this was done in the conventionally used buffer (Trizma-base 40 mM, acetic acid 20 mM, MgCl_2_ 12.5 mM), with or without EDTA, we did not observe any properly shaped objects, regardless of the incubation time (Supplementary Fig. 1, left and middle), in agreement with a previous report^26^. We attribute this effect to the formation of kinetically trapped structures, for which the magnesium-stabilized base pairing would require a higher thermal energy to allow structure reconfigurability^34^. A similar observation was made using Ca^2+^ instead of Mg^2+^; isothermal assembly resulted in properly folded objects only at high temperatures (55–60 °C; Supplementary Fig. 2). We thus opted for an alternative buffer, referred to as TANa, which was solely composed of Tris–acetate buffer (Trizma-base 40 mM, acetic acid 20 mM), without EDTA or magnesium/calcium, to promote staple exchange and reconfiguration. It was supplemented with a monovalent salt (NaCl) to ensure sufficient electrostatic screening between the repulsive anionic DNA strands. Remarkably, with [NaCl] = 100 mM (Debye length *λ*_D_ ≈ 0.8 nm), we observed the progressive formation of properly folded sharp triangles in a few hours at 25 °C (Fig. 1b). We repeated the isothermal procedure for various fixed temperatures and NaCl concentrations and characterized the folding after 24 h of self-assembly without any staple purification (Fig. 1c). Without or with a high amount (500 mM) of NaCl, we could not detect any properly folded origamis for all tested temperatures. In contrast, for intermediate concentrations ([NaCl] = 50–250 mM), a fraction of partially or fully folded origamis was obtained for different temperatures between 15 °C and 60 °C (Fig. 1c and Supplementary Fig. 3). Sufficient electrostatic screening was thus necessary to allow base pairing between anionic DNA strands but should not be too high to maintain some short-distance electrostatic repulsion and hamper the formation of mismatched assemblies. It was found to be particularly efficient for [NaCl] = 100–150 mM, for which a majority of fully folded origamis was obtained when the assembly temperature was set in the range 25–40 °C with [NaCl] = 100 mM and 15–55 °C with [NaCl] = 150 mM (Supplementary Figs. 3–6). Similar results were obtained with other monovalent salts (Supplementary Text 1 and Supplementary Figs. 7 and 8), confirming that the assembly relies on electrostatically adjusted reconfigurability. We found that this was not specific to the sharp triangle origami shape, as simply changing the initial staple composition allowed us to successfully produce 2D origamis of other target shapes, such as tall rectangles or smileys, by isothermal self-assembly at 25 °C (Supplementary Fig. 9). Close-up AFM analysis revealed that, although the resulting folded origamis were not flawless, their overall shape and internal organization corresponded to the target structure (Fig. 1d) and were similar to those obtained by thermal annealing in the same buffer (Supplementary Fig. 10). Isothermal assembly was operational with a staple excess ranging from 2× to 100×, the best yield being obtained at 40× and 100× (Supplementary Text 2 and Supplementary Figs. 11 and 12). Finally, we found that the structures obtained at 25 °C were stable over several days in the presence of their staples in excess (Supplementary Fig. 1, right). The produced origamis could also be purified from their staples using poly(ethylene glycol) (PEG)-based precipitation^35^ and remained stable at 25 °C over a few days when redispersed in TANa in the absence of excess staples (Supplementary Figs. 13 and 14). We then exploited the isothermal conditions for in situ protein functionalization. We directly mixed the M13 scaffold, staples including biotinylated ones, streptavidin as a model protein, and let the system self-assemble in one pot at 25 °C in TANa for 24 h (Fig. 2a, top). We found that the majority of available biotin sites were occupied by a streptavidin (Supplementary Fig. 15), and most of the bound proteins were found at the positions prescribed by biotinylated staples (Fig. 2a and Supplementary Fig. 16). We also investigated the possibility of preparing scaffold-free DNA nanostructures. We first prepared an SST mix composed of 97 oligonucleotides coding for the so-called R4 rectangle^4^ in TANa and let it self-assemble at 25 °C. AFM observations revealed that the fraction of fully folded R4 rectangles increased slowly but substantially with time, as confirmed by a characteristic band of increasing intensity observed by gel electrophoresis, its purification leading to well-defined R4 rectangles (Fig. 2b). We then mixed nine DNA strands that could organize in self-repeating square units^33^. After 24 h of self-assembly in TANa at 25 °C, nanogrids of extended dimensions were successfully obtained with both 100 mM and 150 mM NaCl (Fig. 2c and Supplementary Fig. 17). All these results demonstrate that isothermal self-assembly in TANa is an electrostatically driven and robust way to generate a variety of user-defined nanostructures in a broad window of working temperatures. With this method, complex DNA strand mixtures spontaneously assemble in several hours into multicomponent structures, either scaffolded or not, and can be in situ functionalized by proteins with site specificity.

## Self-assembly of 3D origamis

**Fig. 3:**
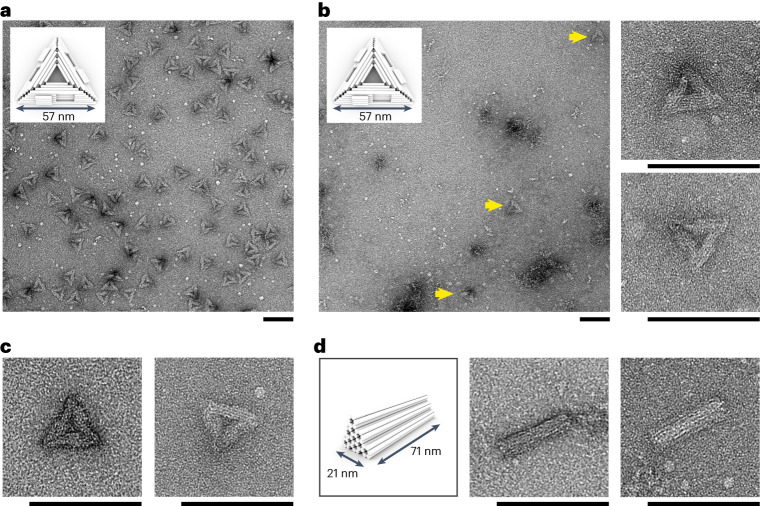
Isothermal self-assembly of elaborate 3D structures at room or body temperature leads to well-shaped 3D origamis at low yield. **a**–**d**, Negative-stain TEM images of the structures obtained by thermal annealing (**a**) or isothermal assembly (**b**–**d**) and after removal of excess staples by gel electrophoresis. **a**, T1 triangular structures (scheme in inset) obtained by 41 h of thermal annealing in an optimized Mg buffer (5 mM Tris–HCl, pH 8.0, 1 mM EDTA, 18 mM MgCl_2_). **b**–**d**, Structures obtained by isothermal self-assembly (no thermal pretreatment) in TANa buffer: T1 triangular structures (scheme in inset) indicated by yellow arrows and obtained with [NaCl] = 100 mM at 25 °C for 48 h (**b**); T1 triangular structures obtained with [NaCl] = 200 mM at 25 °C (left) and with [NaCl] = 100 mM at 37 °C (right) for 72 h (**c**); Tb ‘Toblerone’-like structures (left, scheme) obtained with [NaCl] = 100 mM at 25 °C for 48 h (middle) and with [NaCl] = 200 mM at 25 °C for 48 h (**d**). Scale bars, 100 nm. Source data

We then explored the applicability of our method for the isothermal self-assembly of 3D origamis. We first used a p8064 scaffold and 10× excess of a staple mix coding for a multilayer square-lattice pattern of 24 helix bundles forming a triangular structure with a side length of 57 nm (Fig. 3a,b, insets), called T1 (ref. ^36^). An optimized 41-h-long thermal annealing process in the presence of 18 mM MgCl_2_ led to properly folded structures, as evidenced by transmission electron microscopy (TEM) of the structures after gel electrophoresis purification from their staple excess (Fig. 3a). When the same DNA mix was assembled in TANa ([NaCl] = 100 mM) and incubated at 25 °C for 48 h, only a few triangular structures were obtained (Fig. 3b, left, yellow arrows) but all were well folded, as evidenced by their proper shape, dimensions and 3D arrangements of the helices (Fig. 3b, right). Similar results were obtained with isothermal self-assembly at 25 °C at a higher salt concentration ([NaCl] = 200 mM) or at 37 °C with [NaCl] = 100 mM (Fig. 3c). We applied the same procedure with a p7560 scaffold and a DNA staple mix coding for a denser structure composed of 36-helix bundles packed on a honeycomb lattice pattern and forming a 71-nm-long rod with equilateral triangular section of side length 10 nm, referred to as Tb for its shape similarity with ‘Toblerone’ bars (Fig. 3d, left). Again, isothermal assembly of this mix in TANa at 25 °C led to well-formed Tb structures with both 100 mM and 200 mM NaCl (Fig. 3d) yet at a yield much lower than what can be obtained with thermal annealing (Supplementary Fig. 18). Elaborate and well-assembled 3D nanostructures can thus be obtained by spontaneous self-assembly at both room or body temperature, without any thermal pretreatment, for both hollow and highly dense morphologies, composed of either square or honeycomb helix patterns, further exemplifying the versatility of this self-assembly method. At the same time, the very low yield highlights its current limitations, which might be overcome by staple and design optimization.

## Multiplicity of folding pathways

**Fig. 4:**
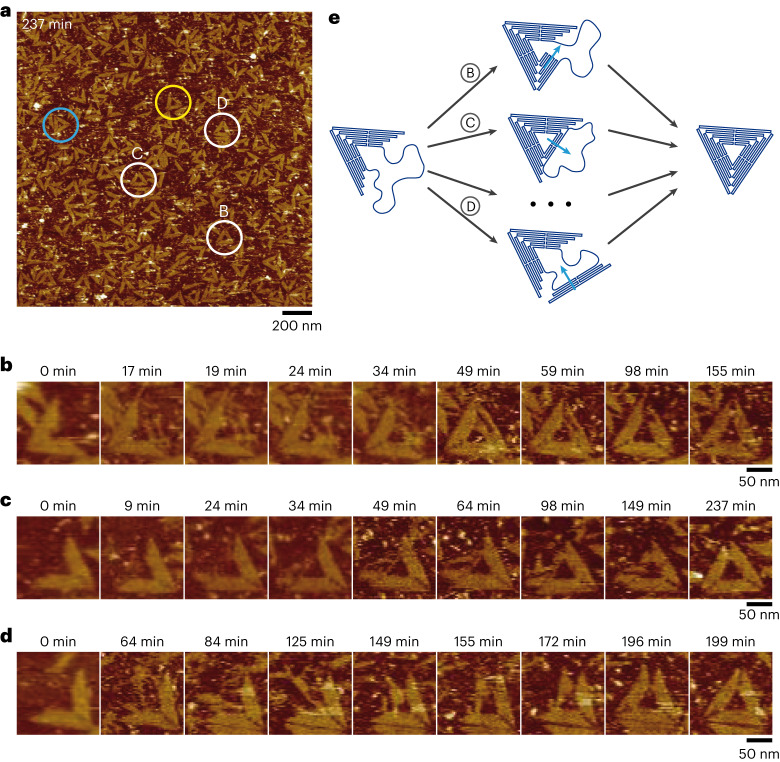
In situ visualization of the isothermal folding reveals the multiple folding pathways of a process under thermodynamic control. **a**, Cholesterol-modified Λ-shaped origamis were initially adsorbed on a supported lipid bilayer and carried an unfolded M13 fragment to be folded into a third sharp triangle side. The image is a snapshot obtained after 237 min of real-time AFM observation at 25 °C in TANa buffer ([NaCl] = 100 mM) supplemented with 1 mM EDTA, where *t* = 0 corresponds to the addition of staples programming the folding of the M13 fragment (Supplementary Movie 1). Circles indicate characteristic examples of (1) complete origami folding from initially adsorbed state (white), (2) complete origami folding in bulk prior to adsorption (blue), (3) adsorption of origamis followed by folding (yellow). **b**–**d**, Detailed evolution of the folding process of the three individual origamis indicated by white circles B (**b**), C (**c**) and D (**d**) in **a** evidencing three characteristic folding pathways from the initial Λ shape to the fully folded sharp triangle. Images are extracted from Supplementary Movies 2–4. **e**, Schematic diagram showing at least three folding pathways. [M13] = 1 nM; each staple concentration is 40 nM.

To characterize the mechanism of the isothermal self-assembly, we devised a method to follow in situ and in real time the folding pathway of 2D DNA origamis (Supplementary Text 3). Briefly, we adsorbed partially folded sharp triangles with a missing side (Λ shape) on a lipid bilayer-coated mica substrate^37^, using cholesterol anchors protruding from the folded sides (Supplementary Fig. 19). Staples of the missing triangle side were then added (*t* = 0), and isothermal folding of the free M13 fragment was followed by continuous AFM imaging at 25 °C inside the TANa buffer (Supplementary Movie 1). We observed that some of the adsorbed structures evolved over time to form well-defined triangular origamis, indicating complete folding of the M13 fragment into a perfectly arranged triangle side (Fig. 4a, white circles). Several fully folded triangles adsorbed during imaging, indicating that folding also occurred in bulk and at a higher speed than on the surface (Fig. 4a, blue circle). Incompletely folded entities also adsorbed from the bulk and continued their folding process (Fig. 4a, yellow circle). A detailed look at the initially adsorbed Λ-shaped origamis evolving to fully folded triangles revealed that individual origamis followed markedly different folding pathways (Fig. 4b–e). One observed pathway consisted of a folding nucleating at one triangle corner and progressing toward the opposite extremity (Fig. 4b and Supplementary Movie 2), a mechanism reminiscent of the DNA assembly nucleation–growth process in SSTs revealed by computer simulation^38^. Folding parallel to the triangle edge was also observed, either from the inside to the outside (Fig. 4c and Supplementary Movie 3) or from the outside to the inside (Fig. 4d and Supplementary Movie 4). These observations reveal that attaining the equilibrium structure for an individual origami is not constrained to one specific folding pathway^39^ but to multiple ones (Fig. 4e), the system escaping kinetic traps through dynamic staple exchange until it reaches its target equilibrium shape. The specific conditions of AFM imaging on a soft surface might affect the folding process itself, for instance, its kinetics. However, the partially folded structures adsorbing during the process (Fig. 4a, yellow circles) also displayed a variety of initial folding states, indicating that the multiplicity of folding pathways is not contingent on surface-assisted assembly. We thus conclude that isothermal origami formation in our conditions is a thermodynamically controlled process where the equilibrium state constituted by properly folded origamis can be spontaneously reached by self-assembly.

## Optimal shape selection and isothermal shape shifting

**Fig. 5:**
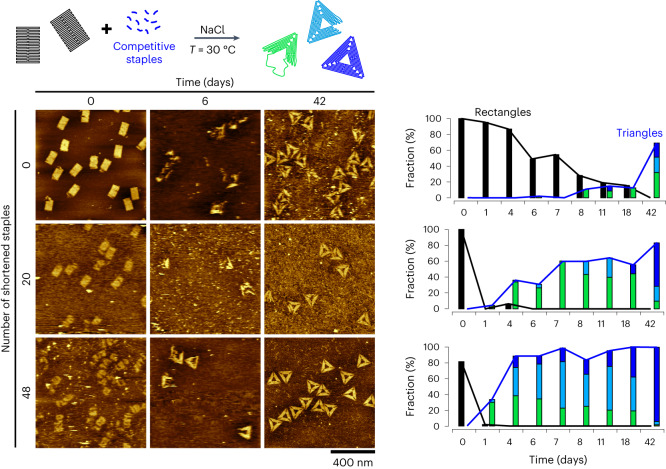
Evolution under thermal equilibrium: morphological transformation by massive strand displacement at constant temperature. Top: schematic principle of the experiment: tall rectangle origamis in the presence of their excess staple (40×) are mixed with a 10-fold larger excess of a competitive set of staples coding for sharp triangles in TANa buffer. The origamis progressively evolve into sharp triangles at constant temperature (30 °C). Bottom left: representative AFM images of the structures evolving in time when the initial set of staples for tall rectangles contained 0, 20 or 48 shortened staples. Bottom right: fraction of detected objects with a partial or fully folded rectangular shape (black) or a triangular shape with one (green), two (light blue) or three (dark blue) well-formed corners, after an increasing incubation time, with 0 (top), 20 (middle) or 48 (bottom) shortened staples in the rectangle. The remaining fraction of each histogram, which corresponds to misshaped or ill-defined objects, is not plotted in this graph. The bars for triangular shapes are shown in a stacked manner; the black and blue lines indicate the cumulative fraction of rectangular and triangular shapes, respectively. All images used for these analyses are available in a citable public repository (doi: 10.5281/zenodo.7998757) and can be accessed directly at the following link: https://zenodo.org/record/7998757. The number *n* of analysed objects for each condition is given in Supplementary Table 3. [M13] = 0.25 nM; each staple concentration is 10 nM (tall rectangle) or 100 nM (sharp triangle). Source data

To further explore the capacity of this self-assembly process to isothermally evolve toward its thermodynamically most stable state, we performed a competition experiment by mixing the M13 scaffold with an equimolar mixture of two sets of staples coding for sharp triangles and staple edge-free tall rectangles. Isothermal assembly in TANa ([NaCl] = 100 mM) at 25 °C led to sharp triangle origamis only, due to their lower free energy level (Supplementary Text 4 and Supplementary Fig. 20), showing that the system was capable of self-selecting its most stable state in a complex pool of competing strands. Then, we prepared tall rectangle origamis in the same buffer by thermal annealing, and exposed them without any purification to additional staples coding for sharp triangles and let the system evolve at a constant temperature (Fig. 5, top row). Remarkably, at 30 °C and with a 10-fold excess of sharp triangle staples, tall rectangles disappeared in time to be progressively replaced with an increasing number of triangular structures. This process was probably facilitated by the large number of unpaired bases on the rectangle edges. With a concentration much lower than that of each staple, the M13 scaffold was the limited reagent in this system. We thus witnessed what appeared to be the first complete origami shape transformation by massive strand displacement of all of its constitutive staples. We further exploited this feature to tune the efficiency and speed of the transformation. To this end, we identified the staples in the tall rectangle structures binding by one extremity to a sequence of eight consecutive bases of the scaffold sequence that was bound by a unique staple in the edges of the sharp triangle structure (Supplementary Materials). Forty-eight staples of the tall rectangle satisfied this criterion. We created shortened versions of these staples by removing the three first (5′ side) or last (3′ side) bases to these specific extremities, and repeated the isothermal transformation experiment using 20 or 48 shortened staples in the initial tall rectangle design. In this case (Fig. 5, middle and bottom rows), tall rectangles disappeared in only 1 day whereas it took over 40 days with full-length staples. This was accompanied by a faster formation of sharp triangles at a rate increasing with an increase in the fraction of shortened staples, that is, increasing amounts of unpaired single-stranded parts to initiate the formation of the triangle edges. With 48 shorter staples, the initial rectangles displayed a looser structure but the transformation was particularly efficient with 88.1% of origamis having a triangular shape with at least one well-formed corner after only 4 days of isothermal transformation. After 42 days, 93.4% of the origamis were perfectly formed sharp triangles. Using a higher salt concentration ([NaCl] = 150 mM) at 30 °C led to the stabilization of the initial tall rectangle structures, and shortening the staples was necessary to observe transformation into sharp triangles (Supplementary Fig. 21). With [NaCl] = 100 mM but at 25 °C, tall rectangles slowly acquired a looser structure but did not transform into triangles within the 54 days of the experiment. Shortening the staples improved the process as the transformation from tall rectangle to well-formed sharp triangles was observed in a few days with a kinetic and efficiency comparable to that at 30 °C (Supplementary Fig. 22). Decreasing the triangle versus rectangle staple ratio from 10 to 1 resulted in a much slower transformation, even in the presence of shortened staples (Supplementary Fig. 23). All these results demonstrated that our self-assembly method makes it possible to achieve spontaneous evolution under thermal equilibrium from one origami shape to a dramatically different one when the system is exposed to a competitive set of staples leading to a thermodynamically more stable shape. Based on a multicomponent massive strand displacement process, the kinetic of this transformation was dependent on the staple composition and could be accelerated and adjusted by increasing the ratio of competitive staples and creating sticky fragments in the initial shape.

## Conclusions

We report here that by using a generic magnesium-free and monovalent saline buffer, highly multicomponent mixtures of DNA strands spontaneously self-assembled at constant temperature, in a broad range of temperatures (15–60 °C), into properly shaped objects either scaffolded (origamis) or not (SST assemblies, DNA nanogrids). Notably, at [NaCl] = 100 mM or 150 mM, all these structures could be efficiently obtained at room temperature, producing highly multicomponent motifs of well-defined size and shapes (origamis, SST) or self-repeating units extending in space (DNA nanogrids), while allowing in situ site-specific functionalization by proteins and programmable stepwise assembly (Supplementary Text 5 and Supplementary Fig. 24). Isothermal self-assembly of well-folded 3D origamis with different shapes, compacities and helix patterns was also achieved at both room temperature and body temperature, but at low yields, a challenge that will likely be addressed by staple and design optimization. This isothermal self-assembly process was shown to be thermodynamically driven. With an ionic buffer composition allowing reconfigurability and folding/assembly pathway exploration, kinetic traps were avoided and isothermal conditions let the DNA mixture progressively evolve until it reached the most stable state defined by the properly assembled nanostructure. This feature opens up horizons for autonomous structure selection and evolution in competitive systems. For instance, we demonstrated that a complex mixture of competitive DNA strands coding simultaneously for different shapes led to successful and selective formation of the most stable shape only; thermodynamically unfavoured competitive shapes, chimera or misfolded structures were avoided. Our demonstration here used only two different competing shapes; it would be interesting to explore more complex mixtures, either to test models with designed strand mixes^39,40^ or to discover new sets of DNA strands producing structures with better shape or functionality, for example, to recognize and/or capture a target protein^41,42^. Finally, we showed that this competitive selection capability was also effective starting from preformed assemblies to produce new dynamic responses. We demonstrated in particular that preformed DNA origamis exposed to a full set of competitive staples could spontaneously evolve and transform into a radically different shape by the exchange of all its constitutive DNA staples through a massive strand-displacement reaction. Thermodynamically driven and adjustable through both staple composition and temperature, this process allows for complex DNA assemblies to morphologically evolve each time a new free energy minimum appears. This constitutes a valuable route to build smart nanomachines capable of morphologically evolving with an encoded predefined dynamics or self-adapting to a changing environment. It opens particularly interesting perspectives for dynamic operations in ambient environment or living systems where the temperature is fixed, and for nanostructure discovery by directed evolution-inspired protocols where improved structures of complex shapes (for example, protein binder) could emerge and be selected from large libraries of DNA components.

## Methods

### Incubation

All incubation processes were carried out in a ThermoMixer C (Eppendorf) except for Supplementary Figs. 1 and 9 (Dry Bath FB15103 incubator, Fisher Scientific) and for Fig. 1c and Supplementary Figs. 3–6 (QuantStudio5, Applied Biosystems by Thermo Fisher Scientific).

### Isothermal self-assembly of 2D DNA origamis

See Fig. 1 and Supplementary Figs. 1–9 and 11–14. We used the staple cocktail without any thermal pretreatment and directly mixed it with the desired buffer prior to brief vortexing and addition of the M13 template (1 nM) to the solution and gentle up-and-down mixing with a pipette. The solution was left to incubate, without further mixing, at a fixed temperature for the desired amount of time.

### Thermal annealing of DNA origamis in TANa buffer

See Supplementary Fig. 10. We assembled the M13 template (1 nM) with a mixture of staples (40 nM each staple) in TANa supplemented with 100 mM of NaCl. The sample was incubated for 10 min at 90 °C and then subjected to a thermal ramp in a peqSTAR 2X thermocycler (Peqlab) from 70 °C to 20 °C at a rate of −1 °C per 10 min.

### Purification by PEG precipitation

See Supplementary Figs. 13 and 14. DNA origamis obtained by isothermal assembly in TANa buffer ([NaCl] = 100 mM) at 25 °C were purified from their staple strands by PEG precipitation. The method was inspired by the protocol introduced in a previous report^35^. The DNA origamis were diluted three times with a solution of PEG 8000 and NaCl to reach final concentrations of 4% w/v and 500 mM, respectively. After gentle mixing, the solution was left to incubate for 15 min at room temperature and centrifuged at 15,000*g* for 15 min. The supernatant was removed and the origamis were resuspended to their initial volume in TANa buffer ([NaCl] = 100 mM). If necessary, the process was repeated for a second consecutive purification.

### Gel electrophoresis of purified origamis

See Supplementary Fig. 13. We prepared 50 ml of agarose (type I low EEO, Sigma Aldrich) gel at 1.5% containing 4 μl of GR-Green 10,000× (Excellgen) in TBE 1× buffer. After the gel had cooled down, we introduced, in each well, 18 µl of 100 bp DNA Ladder (New England Biolabs) or 18 µl of sample supplemented with 1× of DNA loading dye SDS solution (Thermo Scientific). The migration was performed at 100 V for 1 h in a 7 cm electrophoresis cell filled with TBE 1× buffer.

### Isothermal stepwise assembly

See Supplementary Fig. 24. The staples of the triangle were assembled into three separate lots, each coding for the top corner, the intermediate part and the opposite edge. One lot (40 nM each staple) was mixed with M13 (1 nM) in TANa buffer ([NaCl] = 100 mM) and the system was left to incubate at 25 °C without further mixing. Every 24 h, we removed the volume necessary for AFM imaging, added one lot coding for an additional part of the triangle (40 nM each staple) and let the system incubate at 25 °C in TANa buffer ([NaCl] = 100 mM). We performed two different ways of stepwise assembly, from the corner to the opposite side and from one side to the opposite corner.

### Isothermal preparation of streptavidin-modified triangles

See Fig. 2a and Supplementary Figs. 15 and 16. In the same tube, we mixed 1 nM of M13, 40 nM of each of the staples including biotinylated ones, and 2 µM of streptavidin in TANa buffer supplemented with 100 mM of NaCl. The sample was left to incubate at 25 °C without further mixing for 24 h.

### Isothermal preparation of SST R4 rectangles

See Fig. 2b. We mixed all strands of the R4 rectangle in the buffer to a final concentration of 100 nM in each strand in TANa supplemented with 100 mM NaCl. The sample was left to incubate at 25 °C without further mixing for 24 h.

### Gel electrophoresis of SST R4 rectangles

See Fig. 2b. A 1.5% agarose gel (type I low EEO, Sigma Aldrich) was prepared in TBE 0.5× buffer supplemented with 11 mM MgCl_2_ and GB green DNA stain. Gel electrophoresis was performed in an ice-water bath for 2 h at 100 V of voltage using a 1 kb DNA ladder. For purification, the target band of the gel was cut into small pieces and put into a tube with a spin column, and column was subjected to centrifugation at 5,000*g* for 10 min. For AFM imaging, eluted sample was directly adsorbed on a mica plate for 10 min inside the atmosphere-controlled chamber. The sample was then rinsed with 1 ml of in 0.5× TBE + 11 mM MgCl_2_ and observed using AFM in 0.5× TBE + 11 mM MgCl_2_.

### Isothermal preparation of DNA nanogrids

See Fig. 2c and Supplementary Fig. 17. We mixed the nine oligonucleotides (1 µM of each nucleotide) in TANa buffer supplemented with 100 mM or 150 mM of NaCl. The sample was left to incubate at 25 °C without further mixing for 24 h.

### Thermal annealing of 3D origamis

See Fig. 3a and Supplementary Fig. 18. The scaffold (7,560 nt M13 for Tb, 8,064 nt M13 for T1) and the staple mix (10× excess in each staple) were mixed in buffer containing 5 mM Tris–HCl, pH 8.0, 1 mM EDTA and 18 mM MgCl_2_. The mixture was heated to 65 °C for 15 min to denature all DNA strands prior to being slowly cooled down in a gradient from 60 °C to 40 °C, over 41 h to anneal and assemble the 3D origami nanostructures.

### Negative-stain TEM

See Fig. 3 and Supplementary Fig. 18. For TEM characterization, DNA nanostructures were first purified from 1% agarose (0.5× TBE, 45 mM Tris–borate, 1 mM EDTA, pH 8.3) supplemented with 11 mM MgCl_2_ and 0.5 mg ml^−1^ Sybr SAFE. Samples were migrated on the gel for 3 h with a running buffer of 0.5× TBE, 11 mM MgCl_2_ at 2.85 V cm^−1^ at room temperature. Bands corresponding to self-assembled structures were excised and transferred to a DNA gel-extraction spin column (Merck) and centrifuged at 5,000g for 5 min at 4 °C. Purified origamis were then deposited by adsorption onto glow-discharged carbon-coated grid (Quantifoil Micro Tools), stained for 60 s with a 2% (w/v) aqueous uranyl acetate (Merck) solution and then dried with ashless filter paper (VWR). TEM observations were carried out on a JEM-1400 Flash Tungsten microscope working at 120 kV, equipped with a Gatan OneView camera.

### Isothermal competition

See Supplementary Fig. 20. We briefly vortexed the mixtures of two staple sets coding for triangles and rectangles (40 nM final concentration for each staple) with the buffer (TANa supplemented with 100 mM NaCl) prior to adding M13 to the mixture (1 nM final concentration) and gentle mixing with a pipette. The sample was left to incubate at 25 °C without further mixing.

### AFM observation of DNA nanostructures in liquid

Environmental high-resolution AFM observations in sample buffer were used for all AFM data and images shown in this article. Except for Fig. 4 (see the specific protocol below), DNA nanostructures obtained in TANa buffer (DNA origamis with or without protein modification, SST R4 rectangles, DNA nanogrids) were adsorbed on freshly cleaved 10-mm-diameter mica discs (Nano-Tec V-1 grade Muscovite, Micro to Nano Innovative Microscopy Supplies) previously glued to a metal disc and treated with 20 µl of a spermine tetrachloride solution (0.1 M in MilliQ water) for 10 min and washed abundantly, first with MilliQ water and then with the TANa buffer. For sample adsorption, 15–20 µl of sample was deposited on the freshly spermine-treated mica and left to adsorb for 10 min, except for isothermal transformation experiments (Fig. 5 and Supplementary Figs. 21–23) where the time was increased to 20 min due to the lower concentration of origamis. The mica plate was then gently rinsed with 200 µl of the buffer to remove the excess of staples and non-adsorbed objects. To prevent the drying of the sample during mica manipulation, we left a thin layer of buffer on the top of the adsorbed sample and kept it at room temperature in an atmosphere-controlled chamber (a sealed container containing a piece of Kimtech wipe wetted with MilliQ water). The same protocol was done with samples containing magnesium (Supplementary Fig. 1, TAEMg and TAMg buffers) except that the samples were directly adsorbed for 5 min on the freshly cleaved mica without any treatment. The samples were observed with a Cypher ES atomic force microscope (Oxford Instruments) in tapping mode with 17–45 kHz resonance frequency in liquid and a 0.09 N m^−1^ force constant tip (BL-AC40TS, Olympus), using the blueDrive photothermal excitation mode. Raw images were subjected to polynomial background subtraction, plane-level correction, row alignment using various methods and horizontal scar correction in Gwyddion.

### Real-time imaging of the Λ → Δ isothermal evolution on a lipid bilayer surface

See Fig. 4, Supplementary Text 3 and Supplementary Movies 1–4. Supported lipid bilayers (SLBs) were obtained from DOPC liposomes via our previously described method^37^. Vesicles were prepared from a chloroform stock of DOPC. After evaporation of the chloroform under a stream of nitrogen gas, the lipids were rehydrated in MilliQ water to reach a final lipid concentration of 2 mg ml^−1^. The lipid mixture was then vortexed and sonicated for 60 min to produce small unilamellar vesicles. To prevent the drying of the bilayers, the following steps were carried out in an atmosphere-controlled chamber. SLBs were formed by depositing 2 ml of the vesicle solution onto freshly cleaved mica discs (previously stuck on a magnetic metallic plate with glue), followed by 2 µl of TAEMg (Tris–acetate 1×, [EDTA] = 1 mM, [MgCl_2_] = 12.5 mM). After 30 min of adsorption, the sample was rinsed with 2 µl of TAEMg buffer to remove unadsorbed liposomes and this adsorption process was repeated a second time to ensure optimal coverage of the mica surface by the bilayer. At the end of the adsorption process, the sample was rinsed with 5 µl of TAENa (Tris–acetate 1×, [EDTA] = 1 mM, [NaCl] = 100 mM) to ensure that there will be no remaining free Mg^2+^ ions on the sample, which would prevent isothermal folding of the origamis.

Cholesterol-modified Λ-shaped origamis (Supplementary Fig. 19) were prepared by mixing a solution of 10 nM of M13 in the TAENa buffer with 20 nM of each of the staples and annealing by reducing the temperature from 70 °C to 4 °C at a rate of −0.1 °C min^−1^. The resulting origami solution was used without further purification. Then, 2 µl of the cholesterol-modified Λ origamis solution was deposited onto the preformed SLB, followed by 2 µl of TAENa buffer. The sample was incubated for 60 min at room temperature in the atmosphere-controlled chamber, and the surface was then directly imaged at room temperature (*T* = 26 °C) by AFM in 20 µl of TAENa buffer without surface rinsing. After selecting a position containing a sufficient number of Λ origamis adsorbed on the SLB, 8 µl of the A-side staples in TAENa were added onto the sample without moving it or removing it from the AFM stage. The solution overhanging the sample was then gently mixed by slowly moving the AFM probe up and down several times to accelerate the diffusion on the A-side staples towards the mica surface. The same position was then scanned on average every 3 min for 223 min. The image resolution was 512 × 512 from *t* = 0 to *t* = 41 min and was then changed to 640 × 640. The *z* scale of the images was 0–10 nm from *t* = 0 to *t* = 47 min and 0–7 nm afterwards. Because of the evaporation during the imaging process, supplementary buffer (8 µl) was added at *t* = 63 min, *t* = 144 min and *t* = 161 min, and 8 µl of the A-side staples were furthermore added after *t* = 170 min.

AFM images were obtained in TAENa at room temperature using a Brücker Fast Scan atomic force microscope in tapping mode. All AFM experiments were performed using Olympus probes. Images obtained by this protocol are displayed in Fig. 4 and Supplementary Movies 1–4.

### Isothermal morphological transformation

See Fig. 5 and Supplementary Figs. 21–23. DNA rectangle origamis, without or with shortened staples, were first prepared by thermal annealing: after assembly of 1 nM of the M13 template with a mixture of staples (40 nM each) in TANa buffer (with 100 mM or 150 mM NaCl), the sample was incubated for 10 min at 90 °C and then subjected to a thermal ramp in a peqSTAR 2X thermocycler (Peqlab) from 70 °C to 20 °C at a rate of −0.1 °C per 2 min. Triangle staples were then mixed directly to the sample, without any purification (rectangle staples are kept in the system), with a pipette to a desired concentration (10 or 100 nM of each staple) with a final concentration of 0.25 nM in [M13] and 10 nM in each rectangle staple. The resulting sample was left to incubate at 25 °C or 30 °C without further mixing.

### Statistics and reproducibility

Except for the experiment displayed in Fig. 4, which was only performed once due to the set-up complexity, all other investigations were replicated several times to ensure reproducibility. All the AFM image analyses were performed on a large number *n* of individual origamis taken from different images, at different positions of samples obtained in the same conditions. All detected origamis were analysed. No origami, properly folded or not, was excluded from these analyses. The number *n* of analysed objects in each condition shown in the different figures is displayed in Supplementary Tables 1–5. No statistical method was used to predetermine sample size. No data were excluded from the analyses. The experiments were not randomized. The investigators were not blinded to allocation during experiments and outcome assessment.

## Online content

Any methods, additional references, Nature Portfolio reporting summaries, source data, extended data, supplementary information, acknowledgements, peer review information; details of author contributions and competing interests; and statements of data and code availability are available at 10.1038/s41565-023-01468-2.

### Supplementary information

Supplementary Information Materials, Supplementary Figs. 1–24, Texts 1–5, Tables 1–5, legends of movies, references and source data.

Supplementary Video 1 Direct observation of the Λ-to Δ-origami transition on a mica-supported lipid bilayer (DOPC) in the TAENa buffer at room temperature (T = 26 °C). At *t* = 0 min, addition of the missing A-side staples (Supplementary Fig. 19), following the protocol given in Methods section, paragraph “Real-time imaging of the Λ→Δ isothermal evolution on a lipid bilayer surface”. Observation by AFM at the same position over 223 min. Scale bar: 300 nm.

Supplementary Video 2 Crop (375 nm x 375 nm) of Supplementary Movie 1 around the origami labelled B in Fig. 4.

Supplementary Video 3 Crop (375 nm x 375 nm) of Supplementary Movie 1 around the origami labelled C in Fig. 4.

Supplementary Video 4 Crop (375 nm x 375 nm) of Supplementary Movie 1 around the origami labelled D in Fig. 4.

### Source data

Source Data Fig. 1 Source data of Figure 1C

Source Data Fig. 2 Source data of Figure 2A, Uncropped AFM image of Figure 2B, Uncropped AFM image of Figure 2B, Uncropped AFM image of Figure 2B, Uncropped AFM image of Figure 2B, Uncropped AFM image of Figure 2B, Uncropped Gel image of Figure 2B

Source Data Fig. 3 Uncropped image of Figure 3A, Uncropped image of Figure 3B left, Uncropped image of Figure 3B right bottom, Uncropped image of Figure 3B right top, Uncropped image of Figure 3C left, Uncropped image of Figure 3C right, Uncropped image of Figure 3D left, Uncropped image of Figure 3D right

Source Data Fig. 5 Source data of Figure 5

## Data Availability

Most data are available in the main text and the Supplementary Information. Raw data of the graphics and uncropped images (gel, AFM and TEM) of the figures are provided as source data files. If not provided as source data file, images in the figures were not cropped and are displayed as is. Original image data used to generate the different analyses (Figs. 1c, 2a and 5), and cadnano files (.eps and.json) of the origami structures (2D tall rectangle, 2D sharp triangle, 3D T1 triangle, 3D Tb Toblerone) are available in a citable public repository (10.5281/zenodo.7998757) and can be accessed directly at the following link https://zenodo.org/record/7998757. Additional data can be obtained upon request to the corresponding author. Source data are provided with this paper.
