# Supplementary material for: Isothermal self-assembly of multicomponent and evolutive DNA nanostructures
Source: Nat Nanotechnol. 2023 Jul 31;18(11):1311–8. doi: 10.1038/s41565-023-01468-2 (PMC10656289; doi:10.1038/s41565-023-01468-2)
Supplement: Supplementary file 1 — Materials, Supplementary Figs. 1–24, Texts 1–5, Tables 1–5, legends of movies, references and source data. [file 41565_2023_1468_MOESM1_ESM.pdf]

nature nanotechnology

Article

https://doi.org/10.1038/s41565-023-01468-2

Isothermal self-assembly of multicomponent and evolutive DNA nanostructures

In the format provided by the authors and unedited

Table of Contents

1. Materials, page 3

2. Supplementary Figures and Texts

Supplementary Fig. 1, page 25
Supplementary Fig. 2, page 26
Supplementary Fig. 3, page 27
Supplementary Fig. 4, page 28
Supplementary Fig. 5, page 29
Supplementary Fig. 6, page 30
Supplementary Text 1, page 31
Supplementary Fig. 7, page 32
Supplementary Fig. 8, page 33
Supplementary Fig. 9, page 34
Supplementary Fig. 10, page 35
Supplementary Text 2, page 36
Supplementary Fig. 11, page 37
Supplementary Fig. 12, page 38
Supplementary Fig. 13, page 39
Supplementary Fig. 14, page 40
Supplementary Fig. 15, page 41
Supplementary Fig. 16, page 42
Supplementary Fig. 17, page 43
Supplementary Fig. 18, page 44
Supplementary Text 3, page 45
Supplementary Fig. 19, page 46
Supplementary Text 4, page 47
Supplementary Fig. 20, page 48
Supplementary Fig. 21, page 49
Supplementary Fig. 22, page 50
Supplementary Fig. 23, page 51
Supplementary Text 5, page 52
Supplementary Fig. 24, page 53

3. Supplementary Tables 1–5, pages 54–55

4. Legends of supplementary movies, page 56

5. Supplementary references, page 57

6. Supplementary Source data, page 58

1) Materials

Materials. For all experiments except otherwise specified, single-stranded M13mp18 template DNA (105 nM, in 10 mM Tris-HCl and 1 mM EDTA, pH 8.0), abbreviated M13 in this article, was purchased from New England Biolabs. Oligonucleotide staple strands were obtained from Sigma-Aldrich as 100 µM water solutions and used without further purification. For the 3D origamis, the staple strands were from IDT. For the multiple pathway characterization (Fig. 3), M13 template DNA, DNA staples strands and Cholesterol-modified staple strands were obtained from Eurofins Genomics. Affinity purified Pierce^{TM} streptavidin was obtained from Thermo Scientific. Magnesium chloride anhydrous (≥ 98%) ($MgCl_2$), Sodium Chloride (max 0.00002% Al) (NaCl), trizma base (≥ 99.9%), acetic acid (≥ 99.8%), spermine (≥ 97%), Lithium Chloride (≥ 99%, LiCl), Calcium Chloride (≥ 99%, $CaCl_2$), sodium acetate (≥ 99%), Poly(ethylene glycol) for molecular biology mol wt. 8000 (PEG 8000) were obtained from Sigma. Deionized MilliQ water (resistivity 18 MΩ·cm) was used for all experiments.

TANa buffer. For all experiments except otherwise specified, we used a TANa buffer with the following final composition: Trizma-base (40 mmol·L^{-1}), acetic acid (20 mmol·L^{-1}), and a desired concentration of NaCl.

DNA nanostructure design and strand sequences

All sequences are presented below from the 5' (left) to the 3' (right) end.

► For the DNA origami with a sharp triangle and with a smiley design, we used the same staple strand mixes as described in the 2006 article by Rothemund.[1]

► For the tall rectangle origami design, we used the staples sequences described in the 2006 article by Rothemund[1], but we removed staples from the short edges in order to prevent inter-origami stacking. The list of removed staples together with the reference used in the 2006 article by Rothemund[1] is given below:

- Removed edge staples (32 staples):

t7r0f	TGAAAGTATTAAGAGGCTATTATT
t7r10f	AAAAGTAAAACGTCAAAAATGAAAAAACGATT
t7r12f	TTTTGTTTGCTTATCCGGTATTCTAAATCAGA
t7r14f	TATAGAAGACGCGCCTGTTTATCAGTTCAGCT

t7r16f	AATGCAGAGAAAAAGCCTGTTTAGGGAATCAT
t7r18f	AATTACTACATAGGTCTGAGAGACGTGAATTT
t7r20f	ATCAAAATGAAGATGATGAAACAAAATTACCT
t7r22f	GAGCAAAAACTTCTGAATAATGGATGATTGTT
t7r24f	TGGATTATGCCGTCAATAGATAATCAACTAAT
t7r26f	AGATTAGACCAGCAGAAGATAAAAAATACCGA
t7r28f	ACGAACCACTACATTTTGACGCTCACGCTCAT
t7r2f	CTGAAACAGTCAGACGATTGGCCTCAGGAGGT
t7r30j	GGAAATACCAGGAACGGTACGCCATTAAAGGGATTTTAGA
t7r4f	TGAGGCAGGCGTCAGACTGTAGCGATCAAGTT
t7r6f	TGCCTTTAAGACAAAAGGGCGACAGGTTTACC
t7r8f	AGCGCCAAGCAGATAGCCGAACAATTTTTAAG
t-7r10e	CATTCAACCTTGCCCTGACGAGAACATTCAGT
t-7r12e	AAACAGTTTAATGCAGATACATAAGAATACCA
t-7r14e	TTTTTGCGCAGAAAACGAGAATGAAATGCTTT
t-7r16e	TCAATTCTGATGGCTTAGAGCTTAAGAGGTCA
t-7r18e	AGGTAAAGACTAATAGTAGTAGCAAGGTGGCA
t-7r20e	AGAAAAGCATTCAAAAGGGTGAGATAATGTGT
t-7r22e	GATTGACCCCCAAAAACAGGAAGATGATAATC
t-7r24e	CAGCTGGCGTAATGGGATAGGTCAAAACGGCG
t-7r26e	GCATAAAGGAAAGGGGGATGTGCTTATTACGC
t-7r28e	GAGTTGCATGTAAAGCCTGGGGTGAGCCGGAA
t-7r2i	AATAATAAATAGGAACCCATGTACAGGGATAGCAAGCCCA
t-7r30e	ACCCAAATGCAAGCGGTCCACGCTCCCTGAGA
t-7r32e	CAAGTTTTTTGGGGTCGAACCATC
t-7r4e	CAGCGAAATTTTTTCACGTTGAAAGAATTGCG
t-7r6e	CGCCTGATGACAGCATCGGAACGAACCCTCAG
t-7r8e	GAATAAGGAAATTGTGTCGAAATCTGTATCAT

► For the isothermal stepwise assembly (Supplementary Fig. 24), the staples of the sharp triangles were divided into three lots, each one corresponding to the top corner (red part in Supplementary Fig. 24), the intermediate part completing the two sides adjacent to the corner (blue part in Supplementary Fig. 24), or the opposite edge (green part in Supplementary Fig. 24). The list of the staple sequences together with the reference used in the 2006 article by Rothemund[1] is given below for each lot:

- Sequences of the top corner (69 staples):

t11s18h	AATACTGCGGAATCGTAGGGGGTAATAGTAAAATGTTTAGACT
t1s12i	TCATATGTGTAATCGTAAAACTAGTCATTTTC
t1s14i	GTGAGAAAATGTGTAGGTAAAGATACAACTTT
t1s16i	GGCATCAAATTTGGGGCGCGAGCTAGTTAAAG

t1s18i	TTCGAGCTAAGACTTCAAATATCGGGAACGAG
t1s20g	GAATACCACATTCAACTTAAGAGGAAGCCCGATCAAAGCG
t2s11g	AGAAAAGCCCCAAAAAGAGTCTGGAGCAAACAATCACCAT
t2s13g	ACAGTCAAAGAGAATCGATGAACGACCCCGGTTGATAATC
t2s15f	ATAGTAGTATGCAATGCCTGAGTAGGCCGGAG
t2s17f	AACCAGACGTTTAGCTATATTTTCTTCTACTA
t3s14e	CAATATGACCCTCATATATTTTAAAGCATTAA
t3s16e	CATCCAATAAATGGTCAATAACCTCGGAAGCA
t3s18g	AACTCCAAGATTGCATCAAAAAGATAATGCAGATACATAA
t3s20g	CGCCAAAAGGAATTACAGTCAGAAGCAAAGCGCAGGTCAG
t4s11g	GCAAATATTTAAATTGAGATCTACAAAGGCTACTGATAAA
t4s13g	CGTTCTAGTCAGGTCATTGCCTGACAGGAAGATTGTATAA
t4s15f	CAGGCAAGATAAAAATTTTTAGAATATTCAAC
t4s17f	GATTAGAGATTAGATACATTTCGCAAATCATA
t5s14e	TTAATGCCTTATTTCAACGCAAGGGCAAAGAA
t5s16e	TTAGCAAATAGATTTAGTTTGACCAGTACCTT
t5s18g	TAATTGCTTTACCCTGACTATTATGAGGCATAGTAAGAGC
t5s20g	AACACTATCATAACCCATCAAAAATCAGGTCTCCTTTTGA
t6s15g	ATAAAGCCTTTGCGGGAGAAGCCTGGAGAGGGTAG
t6s17f	TAAGAGGTCAATTCTGCGAACGAGATTAAGCA
t7s14e	ATGACCCTGTAATACTTCAGAGCA
t7s16e	TAAAGCTATATAACAGTTGATTCCCATTTTTG
t7s18g	CGGATGGCACGAGAATGACCATAATCGTTTACCAGACGAC
t7s20g	GATAAAAACCAAAATATTAAACAGTTCAGAAATTAGAGCT
t8s17g	TAATTGCTTGGAAGTTTCATTCCAAATCGGTTGTA
t9s16e	ACTAAAGTACGGTGTCGAATATAA
t9s18g	TGCTGTAGATCCCCCTCAAATGCTGCGAGAGGCTTTTGCA
t9s20h	AAAGAAGTTTTGCCAGCATAAATATTCATTGACTCAACATGTT
t-10s27h	AACTCACATTATTGAGTGTTGTTCCAGAAACCGTCTATCAGGG
t-12s29h	ACGTGGACTCCAACGTCAAAGGGCGAATTTGGAACAAGAGTCC
t-1s22i	CGCGTCTGATAGGAACGCCATCAACTTTTACA
t-1s24e	CAGTTTGACGCACTCCAGCCAGCTAAACGACG
t-1s24i	AGGAAGATGGGGACGACGACAGTAATCATATT
t-1s26e	GCCAGTGCGATCCCCGGGTACCGAGTTTTTCT
t-1s26i	CTCTAGAGCAAGCTTGCATGCCTGGTCAGTTG
t-1s28g	TTTCACCAGCCTGGCCCTGAGAGAAAGCCGGCGAACGTGG
t-1s28i	CCTTCACCGTGAGACGGGCAACAGCAGTCACA
t-1s30e	CGAGAAAGGAAGGGAAGCGTACTATGGTTGCT
t-2s21g	GCTCATTTTTTAACCAGCCTTCCTGTAGCCAGGCATCTGC
t-2s23g	GTAACCGTCTTTCATCAACATTAAAATTTTTGTTAAATCA
t-2s25f	ACGTTGTATTCCGGCACCGCTTCTGGCGCATC
t-2s27f	CCAGGGTGGCTCGAATTCGTAATCCAGTCACG
t-3s24e	TGTAGATGGGTGCCGGAAACCAGGAACGCCAG
t-3s26e	GGTTTTCCATGGTCATAGCTGTTTGAGAGGCG

t-3s28g	GTTTGCGTCACGCTGGTTTGCCCCAAGGGAGCCCCCGATT
t-3s30g	TAGAGCTTGACGGGGAGTTGCAGCAAGCGGTCATTGGGCG
t-4s21g	GTTAAAATTCGCATTAATGTGAGCGAGTAACACACGTTGG
t-4s23g	GGATAGGTACCCGTCGGATTCTCCTAAACGTTAATATTTT
t-4s25f	AGTTGGGTCAAAGCGCCATTCGCCCCGTAATG
t-4s27f	CGCGCGGGCCTGTGTGAAATTGTTGGCGATTA
t-5s26e	TGCTGCAAATCCGCTCACAATTCCCAGCTGCA
t-5s28g	TTAATGAAGTTTGATGGTGGTTCCGAGGTGCCGTAAAGCA
t-5s30g	CTAAATCGGAACCCTAAGCAGGCGAAAATCCTTCGGCCAA
t-6s23f	CGGCGGATTGAATTCAGGCTGCGCAACGGGGGATG
t-6s25c	TGGCGAAATGTTGGGAAGGGCGAT
t-6s27f	TGTCGTGCACACAACATACGAGCCACGCCAGC
t-7s28g	TTCCAGTCCTTATAAATCAAAAGAGAACCATCACCCAAAT
t-7s30g	CAAGTTTTTTGGGGTCGAAATCGGCAAAATCCGGGAAACC
t-8s25f	TCTTCGCTATTGGAAGCATAAAGTGTATGCCCGCT
t-8s27c	GCGCTCACAAGCCTGGGGTGCCTA
t-9s30g	CGATGGCCCACTACGTATAGCCCGAGATAGGGATTGCGTT
t-5s22e-t6s13c-3T	GTGGGAACAAATTTCTATTTTTGAG
t-7s24e-t8s15c-2T	CGGTGCGGGCCTTCCAAAAACATT
t-9s26e-t10s17c-1T	ATGAGTGAGCTTTTAAATATGCA
t-11s28e-t12s19c-0T	ACTATTAAAGAGGATAGCGTCC

- Sequences of the intermediate part (65 staples):

t11s28h	TCTTTGATTAGTAATAGTCTGTCCATCACGCAAATTAACCGTT
t1s22i	TCGGGAGATATACAGTAACAGTACAAATAATT
t1s24i	CCTGATTAAAGGAGCGGAATTATCTCGGCCTC
t1s26i	GCAAATCACCTCAATCAATATCTGCAGGTCGA
t1s28i	CGACCAGTACATTGGCAGATTCACCTGATTGC
t1s30g	TTGACGAGCACGTATACTGAAATGGATTATTTAATAAAAG
t2s21g	CCTGATTGCTTTGAATTGCGTAGATTTTCAGGCATCAATA
t2s23g	TGGCAATTTTTAACGTCAGATGAAAACAATAACGGATTCG
t2s25f	AAGGAATTACAAAGAAACCACCAGTCAGATGA
t2s27f	GGACATTCACCTCAAATATCAAACACAGTTGA
t3s24e	TAATCCTGATTATCATTTTGCGGAGAGGAAGG
t3s26e	TTATCTAAAGCATCACCTTGCTGATGGCCAAC
t3s28g	AGAGATAGTTTGACGCTCAATCGTACGTGCTTTCCTCGTT
t3s30g	AGAATCAGAGCGGGAGATGGAAATACCTACATAACCCTTC
t4s21g	GCGCAGAGGCGAATTAATTATTTGCACGTAAATTCTGAAT
t4s23g	GATTATACACAGAAATAAAGAAATACCAAGTTACAAAATC
t4s25f	TAGGAGCATAAAAGTTTGAGTAACATTGTTTG
t4s27f	TGACCTGACAAATGAAAAATCTAAAATATCTT
t5s24e	AATGGAAGCGAACGTTATTAATTTCTAACAAC

t5s26e	TAATAGATCGCTGAGAGCCAGCAGAAGCGTAA
t5s28g	GAATACGTAACAGGAAAAACGCTCCTAAACAGGAGGCCGA
t5s30g	TTAAAGGGATTTTAGATACCGCCAGCCATTGCGGCACAGA
t6s25g	TCAATAGATATTAAATCCTTTGCCGGTTAGAACCT
t6s27f	CAATATTTGCCTGCAACAGTGCCATAGAGCCG
t7s24e	ACAATTCGACAACTCGTAATACAT
t7s26e	TTGAGGATGGTCAGTATTAACACCTTGAATGG
t7s28g	CTATTAGTATATCCAGAACAATATCAGGAACGGTACGCCA
t7s30g	GAATCCTGAGAAGTGTATCGGCCTTGCTGGTACTTTAATG
t8s27g	CGCGAACTAAAACAGAGGTGAGGCTTAGAAGTATT
t9s26e	ACCACCAGCAGAAGATGATAGCCC
t9s28g	TAAAACATTAGAAGAACTCAAACTTTTTATAATCAGTGAG
t9s30h	GCCACCGAGTAAAAGAACATCACTTGCCTGAGCGCCATTAAAA
t-10s17h	ACCAACCTAAAAAATCAACGTAACAAATAAATTGGGCTTGAGA
t-12s19h	CCTGACGAGAAACACCAGAACGAGTAGGCTGCTCATTCAGTGA
t-1s12i	AGGGATAGCTCAGAGCCACCACCCCATGTCAA
t-1s14e	ATTTTCTGTCAGCGGAGTGAGAATACCGATAT
t-1s14i	CAACAGTTTATGGGATTTTGCTAATCAAAAGG
t-1s16e	ATTCGGTCTGCGGGATCGTCACCCGAAATCCG
t-1s16i	GCCGCTTTGCTGAGGCTTGCAGGGGAAAAGGT
t-1s18g	CGACCTGCGGTCAATCATAAGGGAACGGAACAACATTATT
t-1s18i	GCGCAGACTCCATGTTACTTAGCCCGTTTTAA
t-1s20e	ACAGGTAGAAAGATTCATCAGTTGAGATTTAG
t-2s11g	CCTCAGAACCGCCACCCAAGCCCAATAGGAACGTAAATGA
t-2s13g	AGACGTTACCATGTACCGTAACACCCCTCAGAACCGCCAC
t-2s15f	CACGCATAAGAAAGGAACAACTAAGTCTTTCC
t-2s17f	ATTGTGTCTCAGCAGCGAAAGACACCATCGCC
t-3s14e	GTTTTGTCAGGAATTGCGAATAATCCGACAAT
t-3s16e	GACAACAAGCATCGGAACGAGGGTGAGATTTG
t-3s18g	TATCATCGTTGAAAGAGGACAGATGGAAGAAAAATCTACG
t-3s20g	TTAATAAAACGAACTAACCGAACTGACCAACTCCTGATAA
t-4s11g	AGGTTTAGTACCGCCATGAGTTTCGTCACCAGGATCTAAA
t-4s13g	AGCGTAACTACAAACTACAACGCCTATCACCGTACTCAGG
t-4s15f	TAGTTGCGAATTTTTTCACGTTGATCATAGTT
t-4s17f	GTACAACGAGCAACGGCTACAGAGGATACCGA
t-5s16e	AACAGCTTGCTTTGAGGACTAAAGCGATTATA
t-5s18g	CCAAGCGCAGGCGCATAGGCTGGCAGAACTGGCTCATTAT
t-5s20g	ACCAGTCAGGACGTTGGAACGGTGTACAGACCGAAACAAA
t-6s13f	ACAGACAGCCCAAATCTCCAAAAAAAAATTTCTTA
t-6s15c	CGAGGTGAGGCTCCAAAAGGAGCC
t-6s17f	ACCCCCAGACTTTTTCATGAGGAACTTGCTTT
t-7s18g	AAAACACTTAATCTTGACAAGAACTTAATCATTGTGAATT
t-7s20g	ACCTTATGCGATTTTATGACCTTCATCAAGAGCATCTTTG
t-8s15f	CGGTTTATCAGGTTTCCATTAAACGGGAATACACT

t-8s17c	GGCAAAAGTAAAATACGTAATGCC
t-9s20g	TGGTTTAATTTCAACTCGGATATTCATTACCCACGAAAGA

- Sequence of the opposite edge (73 staples):

t11s8h	CAGAAGGAAACCGAGGTTTTTAAGAAAAGTAAGCAGATAGCCG
t1s10g	GACGGGAGAATTAACTCGGAATAAGTTTATTTCCAGCGCC
t1s2i	CGGGGTTTCCTCAAGAGAAGGATTTTGAATTA
t1s4i	AGCGTCATGTCTCTGAATTTACCGACTACCTT
t1s6i	TTCATAATCCCCTTATTAGCGTTTTTCTTACC
t1s8i	ATGGTTTATGTCACAATCAATAGATATTAAAC
t2s1g	GATAAGTGCCGTCGAGCTGAAACATGAAAGTATACAGGAG
t2s3g	TTTGATGATTAAGAGGCTGAGACTTGCTCAGTACCAGGCG
t2s5f	CCGGAACCCAGAATGGAAAGCGCAACATGGCT
t2s7f	AAAGACAACATTTTCGGTCATAGCCAAAATCA
t3s10g	GTCAGAGGGTAATTGATGGCAACATATAAAAGCGATTGAG
t3s4e	TGTACTGGAAATCCTCATTAAAGCAGAGCCAC
t3s6e	CACCGGAAAGCGCGTTTTCATCGGAAGGGCGA
t3s8g	CATTCAACAAACGCAAAGACACCAGAACACCCTGAACAAA
t4s1g	TAGCCCGGAATAGGTGAATGCCCCCTGCCTATGGTCAGTG
t4s3g	TTTAACGGTTCGGAACCTATTATTAGGGTTGATATAAGTA
t4s5f	CTCAGAGCATATTCACAAACAAATTAATAAGT
t4s7f	GGAGGGAATTTAGCGTCAGACTGTCCGCCTCC
t5s10g	GATAACCCACAAGAATGTTAGCAAACGTAGAAAATTATTC
t5s4e	CCTTGAGTCAGACGATTGGCCTTGCGCCACCC
t5s6e	TCAGAACCCAGAATCAAGTTTGCCGGTAAATA
t5s8g	TTGACGGAAATACATACATAAAGGGCGCTAATATCAGAGA
t6s5g	CAGAGCCAGGAGGTTGAGGCAGGTAACAGTGCCCG
t6s7f	ATTAAAGGCCGTAATCAGTAGCGAGCCACCCT
t7s10g	ATAAGAGCAAGAAACATGGCATGATTAAGACTCCGACTTG
t7s4e	GCCGCCAGCATTGACACCACCCTC
t7s6e	AGAGCCGCACCATCGATAGCAGCATGAATTAT
t7s8g	CACCGTCACCTTATTACGCAGTATTGAGTTAAGCCCAATA
t8s7g	AGCCATTTAAACGTCACCAATGAACACCAGAACCA
t9s10h	TATCTTACCGAAGCCCAAACGCAATAATAACGAAAATCACCAG
t9s6e	CCATTAGCAAGGCCGGGGGAATTA
t9s8g	GAGCCAGCGAATACCCAAAAGAACATGAAATAGCAATAGC
t-10s7h	ACGACAATAAATCCCGACTTGCGGGAGATCCTGAATCTTACCA
t-12s9h	TGCTATTTTGCACCCAGCTACAATTTTGTTTTGAAGCCTTAAA
t-1s10e	AGAGAATAACATAAAAACAGGGAAGCGCATTA
t-1s2i	CCTTTTTTCATTTAACAATTTCATAGGATTAG
t-1s4e	TTATCAAACCGGCTTAGGTTGGGTAAGCCTGT
t-1s4i	TTTAACCTATCATAGGTCTGAGAGTTCCAGTA

t-1s6e	TTAGTATCGCCAACGCTCAACAGTCGGCTGTC
t-1s6i	AGTATAAAATATGCGTTATACAAAGCCATCTT
t-1s8g	TTTCCTTAGCACTCATCGAGAACAATAGCAGCCTTTACAG
t-1s8i	CAAGTACCTCATTCCAAGAACGGGAAATTCAT
t-2s1g	AAAACAAAATTAATTAAATGGAAACAGTACATTAGTGAAT
t-2s3g	AGAGTCAAAAATCAATATATGTGATGAAACAAACATCAAG
t-2s5f	ACTAGAAATATATAACTATATGTACGCTGAGA
t-2s7f	TCAATAATAGGGCTTAATTGAGAATCATAATT
t-3s10g	AACGTCAAAAATGAAAAGCAAGCCGTTTTTATGAAACCAA
t-3s4e	GATTAAGAAATGCTGATGCAAATCAGAATAAA
t-3s6e	CACCGGAATCGCCATATTTAACAAAATTTACG
t-3s8g	AGCATGTATTTCATCGTAGGAATCAAACGATTTTTTGTTT
t-4s1g	GAGCAAAAGAAGATGAGTGAATAACCTTGCTTATAGCTTA
t-4s3g	ACATAGCGCTGTAAATCGTCGCTATTCATTTCAATTACCT
t-4s5f	GTTAAATACAATCGCAAGACAAAGCCTTGAAA
t-4s7f	CCCATCCTCGCCAACATGTAATTTAATAAGGC
t-5s10g	TCCCAATCCAAATAAGATTACCGCGCCCAATAAATAATAT
t-5s6e	GTGTGATAAGGCAGAGGCATTTTCAGTCCTGA
t-5s8g	ACAAGAAAGCAAGCAAATCAGATAACAGCCATATTATTTA
t-6s3f	TCCCTTAGAATAACGCGAGAAAACTTTTACCGACC
t-6s5c	GTTTGAAATTCAAATATATTTTAG
t-6s7f	AATAGATAGAGCCAGTAATAAGAGATTTAATG
t-7s10g	GCCAGTTACAAAATAATAGAAGGCTTATCCGGTTATCAAC
t-7s8g	GCGCCTGTTATTCTAAGAACGCGATTCCAGAGCCTAATTT
t-8s5f	TTCTGACCTAAAATATAAAGTACCGACTGCAGAAC
t-8s7c	TCAGCTAAAAAAGGTAAAGTAATT
t-9s10g	ACGCTAACGAGCGTCTGGCGTTTTAGCGAACCCAACATGT
t-5s2e-t6s23c-3T	TTAATTAATTTTTTACCATATCAAA
t-7s4e-t8s25c-2T	TTAATTTCATCTTAGACTTTACAA
t-9s6e-t10s27c-1T	CTGTCCAGACGTATACCGAACGA
t-11s8e-t12s29c-0T	TCAAGATTAGTGTAGCAATACT
t-5s12e-t6s3c-3T	TGTAGCATTCCTTTTATAAACAGTT
t-7s14e-t8s5c-2T	TTTAATTGTATTTCCACCAGAGCC
t-9s16e-t10s7c-1T	ACTACGAAGGCTTAGCACCATTA
t-11s18e-t12s9c-0T	ATAAGGCTTGCAACAAAGTTAC

► For the protein-functionalized sharp triangle origamis (Figs. 2A, Supplementary Figs. 15,16), we used the staples sequences from the same reference and we replaced 6 staples localized in the corners by 6 modified staples having the same sequence (red) with a biotin-TEG modification [Btn-TEG] separated by an additional TTTT spacer (blue):

[BtnTEG]TTTTTAATTGCTTGGAAGTTTCATTCCAAATCGGTTGTA

[BtnTEG]TTTTCGCGAACTAAAACAGAGGTGAGGCTTAGAAGTATT

[BtnTEG]TTTTAGCCATTTAAACGTCACCAATGAACACCAGAACCA
TCTTCGCTATTGGAAGCATAAAGTGTATGCCCGCTTTTT[BtnTEG]
CGGTTTATCAGGTTTCCATTAAACGGGAATACACTTTTT[BtnTEG]
TTCTGACCTAAAATATAAAGTACCGACTGCAGAACTTTT[BtnTEG]

►For the rectangle-to-triangle isothermal transformation (Figs. 5, Supplementary Figs. 21–23), some experiments were done with shortened staples in the initial rectangle origami design. We used the rectangle design after the staple edges being removed (see details before) and we targeted the staples binding by one extremity to a sequence of 8 consecutive bases of the scaffold sequence that was bound by a unique staple in the edges of the triangle structure. To select the identify and select the staples to shorten, we first identified the parts of the scaffold binding to the 63 staples involved in the edges of the triangle. We analyzed the rectangle staples binding to these specific parts of the scaffold and selected those binding by an extremity with at least 8 bases in common with a triangle staple. We found 48 staples in the rectangle design satisfying this criteria. The shortening was done by removing the three first bases when the extremity with 8 common bases was at the 5' or the three last bases when the extremity with 8 common bases was at the 3'end. When a rectangle staple had both extremities having 8 common bases with two different triangle staples, we used a mixture of the staple shortened at the 5' end and the staple shortened at the 3' end, while keeping the overall concentration of the shortened staple identical to the concentration of each other staple. For rectangles with 48 shortened staples, we used the staple mix of the rectangle described above (after removing the staple edges) and replaced the 48 staples by their shortened version. For rectangles with 20 shortened staples, we selected 20 staples among the 48 candidates to ensure a homogenous distribution of the shortened staples in the rectangle design. We thus used the staple mix of the rectangle and replaced the 20 selected staples by their shortened version. The list of the shortened staples is given below together with their schematic position in the rectangle design as well as the reference of the corresponding complete sequence used in the 2006 article by Rothemund[1]:

- Sequences of the 20 shortened staples:

Rothemund's nomenclature	
t5r14e	GTCCTGCGCCCAATAGCAAGCAAGAACGC
t3r12f	AATTTAAGCCTTAAATCAAGAATCGAGAACCTAATTTAAGCC TTAAATCAAGAATCGA
t-3r20e	AGAGAATCAGCTGATAAATTAATGCTTTA
t5r24f	GGCAAAAGTATTAGACTTTACAAGGTTAT
t-1r8e	CCAGGCGCGAGGACAGATGAACGGTAGA
t1r18e	AAGAAAATTTCATCTTCTGACAGAATCGC

t5r10f ATAGCAGAGAATAACATAAAAACAGCCATGCAATAGCAGAG AATAACATAAAAACAGC
t-3r18f CAAAATTAGGATAAAAATTTTTAGGATAT
t5r26f CTAAAATAAGTATTAACACCGCCTCGAAC
t-1r14f AGGAACGAGCTTCAAAGCGAAAGTTTCAT
t-5r10f ACGAGTAGATCAGTTGAGATTTAGCGCCA
t-5r12f ATTACCATTGAATCCCCCTCACCATAAAT
t5r16f GACGACAAATTCTTACCAGTAGATAAATA
t-1r28f GCCAGCTGCGGTTTGCGTATTGGGAATCA
t-3r28f ACTGCCCGCTTTTCACCAGTGAGATGGTG
t-5r28f GTGAGCTAGCCCTTCACCGCCTGGGGTTT
t-1r22e TAATTTTTAACCAATAGGAACAACAGTAC
t-1r6f CGTAAGAGGCAAAAGAATACACTGACCAAATACGTAAGAGGC AAAAGAATACACTGAC
t3r22e ACAGAAATCTTTGAATACCAAGTTAATTT
t1r30f ACTTGAATACTTCTTTGATTAGTTGTTCC

Schematic representation of the position of the 20 shortened staples, indicated by a yellow star, in the rectangle design:

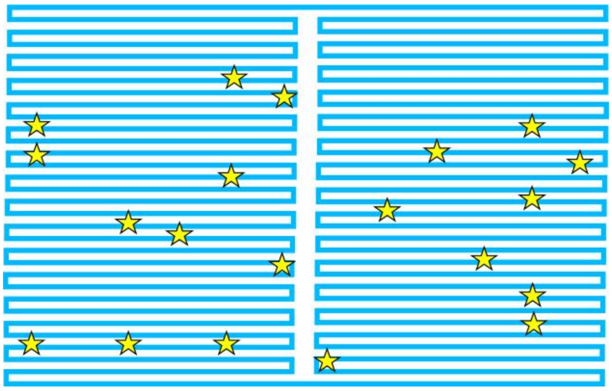

- Sequences of the 48 shortened staples:

Rothemund's nomenclature
t1r12f TACCAACCCAGCTACAATTTTAAAGAAGT
t-1r12e TTTGCCAGGCGAGAGGCTTTTGCAATCCT
t5r14e GTCCTGCGCCCAATAGCAAGCAAGAACGC
t3r12f AATTTAAGCCTTAAATCAAGAATCGAGAACCTAATTTAAGCCT TAAATCAAGAATCGA
t5r16e GCGTTATACGACAATAAACAACATACAAT
t5r12f ATTTATTAGCGAACCTCCCGACGTAGGAAATTATTTATTAGCGA ACCTCCCGACGTAG
t-5r18f TAAATAAATGCAATGCCTGAGAAGGCCGG
t3r14f CAAGCAAGCGAGCATGTAGAAACCAGAGA
t-3r20e AGAGAATCAGCTGATAAATTAATGCTTTA
t-5r8f GCTCCATGACGTAACAAAGCTGCTACACC

t3r8f	CAAAGAAGAACTGGCATGATTTGAGTTAAACGCAAAGAAGAA CTGGCATGATTTGAGT
t5r24f	GGCAAAAGTATTAGACTTTACAAGGTTAT
t1r8f	ATACACAGTATGTTAGCAAACTGTACAGA
t-1r10e	GGACGTTGAGAACTGGCTCATTATGCGCT
t1r10f	AGAGAGTCAGAGGGTAATTGAACCAGTCAATCAGAGAGTCAG AGGGTAATTGAACCAG
t1r12e	TATTTTGCACGCTAACGAGCGTCTGAACA
t-1r8e	CCAGGCGCGAGGACAGATGAACGGGTAGA
t3r10f	CAATAGACGGGAGAATTAACTTTCCAGAGGCCCAATAGACGG GAGAATTAACTTTCCA
t1r18e	AAGAAAATTTCATCTTCTGACAGAATCGC
t5r10f	ATAGCAGAGAATAACATAAAAACAGCCATGCAATAGCAGAGA ATAACATAAAAACAGC
t5r8f	TCACAATCCCGAGGAAACGCAATAATGAA
t1r6f	CACCACCATTTGGGAATTAGACCAACCTA
t-3r10f	CAACTACGGAACAACATTATTAACACTATTTTCAACTACGGAA CAACATTATTAACAC
t-3r8f	ACGGTCAATGACAAGAACCGGATATGGTT
t-3r18f	CAAAATTAGGATAAAAATTTTTAGGATAT
t5r14f	TTACCGAACAAGAAAAATAATAATTCTGT
t-1r8f	CTTTGAAAATAGGCTGGCTGACCTACCTT
t-1r10f	TTTTAGGAAGAAAAATCTACGGATAAAAACGATTTTAGGAAGA AAAATCTACGGATAA
t5r26f	CTAAAATAAGTATTAACACCGCCTCGAAC
t-1r22e	TAATTTTTAACCAATAGGAACAACAGTAC
t-1r14f	AGGAACGAGCTTCAAAGCGAAAGTTTCAT
t-5r10f	ACGAGTAGATCAGTTGAGATTTAGCGCCA
t-3r14f	GAAGCCTCCAACAGGTCAGGATTTAAATA
t-5r12f	ATTACCATTGAATCCCCCTCACCATAAAT
t-3r12f	AACCCGCGTCCAATACTGCGGTATTATAG
t-5r14f	AAATCATTGCTCCTTTTGATAATTGCTGA
t5r16f	GACGACAAATTCTTACCAGTAGATAAATA
t-1r12f	AAATAAGGGGGTAATAGTAAAAAAAGATT
t-1r16f	TCCATATATTTAGTTTGACCATTAAGCAT
t-5r12e	ATATTGAGGCATAGTAAGAGCACAGGTAG
t-1r28f	GCCAGCTGCGGTTTGCGTATTGGGAATCA
t-3r28f	ACTGCCCGCTTTTCACCAGTGAGATGGTG
t-5r28f	GTGAGCTAGCCCTTCACCGCCTGGGGTTT
t5r8e	GGAAAAATAGAAAATTCATATTTCAACCG
t-1r6f	CGTAAGAGGCAAAAGAATACACTGACCAAATACGTAAGAGGC AAAAGAATACACTGAC
t3r22e	ACAGAAATCTTTGAATACCAAGTTAATTT
t3r6f	CCGGAAACTAAAGGTGAATTATCATAAAA
t1r30f	ACTTGAATACTTCTTTGATTAGTTGTTCC

Schematic representation of the 48 shortened staples, indicated by a yellow star, in the rectangle design:

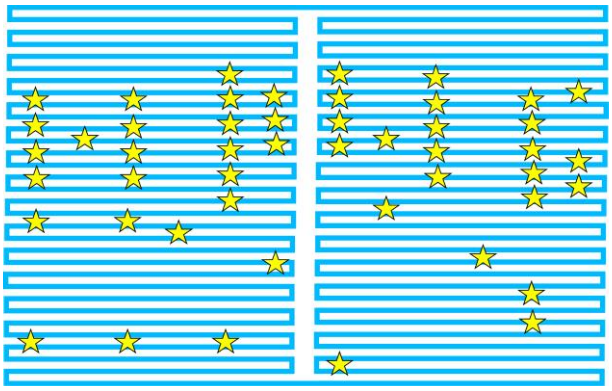

► For the single-stranded tile (SST) R4-rectangles (Fig. 2B), we used the strand mix for R4-rectangle described in the 2012 article by Wei *et al.*[2]

► For DNA nanogrids (Fig. 2C, Supplementary Fig. 17), we used a mixture of 9 DNA oligonucleotides described the 2003 article by Yan *et al.*[3]:

1 GGATAGCGCCTGATCGGAACGCCTACGATGGACACGCCGACC
2 GGCGTGTGGTTGC
3 GAGCGCAACCTGCCTGGCAAGACTCCAGAGGACTACTCATCC
4 ATCGGATGAGTAGTGGGCTCAGTGGAG
5 GACTGAGCCCTGCTAGGATCGACTTCACTGGACCGTTCTACC
6 GCTCGGTAGAACGGTGGAAGCCAACGGTC
7 GTTGGCTTCCTGTACGGCAGAACTCCGTTGGACGAACACTCC
8 TGTTCGTGGCGCT
9 AGGCACCATCGTAGGTTTTCGTTCCGATCACCAACGGAGTTTTTCTGCCGTACA
CCAGTGAAGTTTTTCGATCCTAGCACCTCTGGAGTTTTTCTTGCC

► For the T1 3D origamis (triangular structure, Figs. 3A–C), we used the CaDNAno file published by Sigl *et al.*[4] consisting of a 8064-nt M13 scaffold named p8064, and the following staples:

T1_01	GCTCATGGAGCACCAGCAGAAGATAAAACAGAGGTGACAAATCAACA
T1_02	TCGGCGAACTCCGTGGTGAAGGGAAAGATGATAACCTATTGTTAATGC
T1_03	ATAATTCGCATTAAATGTGATTGAATCCCTTTTTCTCA

T1_04	GGAAACCACGGTGCGGGCCTCTTCAGCCCAATGTATAAGCAAAAGCCC
T1_05	CTGGTGTGTTCAGCAAATCAGCGGGAGCTAATATCTTCTTTG
T1_06	TTTTTATTTTCATCGTAGGAATCACAGACGACGAC
T1_07	TTATTTATCCCCCAATAGCAAGCAAATCAGATTTTTTTAGAAGGCTTATCCG
T1_08	ATGACCCAATCACCATCAATATGATTTTTTTTCATTGACCATTAGATACA
T1_09	GCTGCAACAAGGGAAGAAAGTTTTTGAAAGGAGCGGGCCTTGACGG
T1_10	AGGTAAATATTGTTTTTCGGAAATTATTCATGCCAGTT
T1_11	ACAAAGTACAATTCCTGAGTTT
T1_12	AAACTTAAAAAGAGACGCAGAAACGAAAGGATTAGGATTATGCCGTC
T1_13	GATAAGAGAAGTACGGGATTTAGTACCGTTCTATTTTTCTGATAAA
T1_14	AAAATCAGTTCATCAACGTCTGGCCTTCCTGTAATTGCGT
T1_15	CCTTATGCGGTGGTTCCGAAATCGGAAAATCCTGTTTGAT
T1_16	AGTAAATGAATTTCTCCAAAATGAGGCTTACGATAAAAACGCCAA
T1_17	ACGTTGGTGGATTGACCGTAATGGATATTATTTGCACGTAAGGTTTA
T1_18	TAATTACTAGAAATCAAGAATCCTGAATCTTACCGCCATTTGCAATCAAT
T1_19	TTCACCAGTGACACTATCATAACCGGAAGCATCAGAAGC
T1_20	CATATATTAATCAGTAGCGAGGAACCGCCT
T1_21	GGCGATTATGGCGAAAGGGGGATGAATAAATTTTTGTTAAATCAAAA
T1_22	AAAACGACGGCCAGTGAATCAATAAAATAGCACTAATATC
T1_23	ATCATACAGGCAAGGGGAACGCCATCAGCTCATTTTTT
T1_24	AAATATCAATAAGAAACGATTTTTTTTTTGTTTAACGTACAAAATA
T1_25	TTAAAGGTGCCAAAGACAAAAGGGCTTTTTACATTCAACCGATTGA
T1_26	ATCTTGACAAGAACCGGATATTCACCAAGCGCGAA
T1_27	TGATATAAGTATAGCCAACCAATACAAAGAATTAATTAATATTTTGT
T1_28	GCGGCCTTCCCACGCAACTTTTTAGCTTACGGCTGTGCCGGTG
T1_29	ATGGTCAATAACCAGCACTAACAACTTTGCCCGAACGGTTGATTCCCA
T1_30	GCTAGGGCAGCTAACTGGTTTCTGCCAGCACGTACCTGCACACGTATA
T1_31	CCATATTTAACAAGTAATTTACTCCCGATTTCCAGAGCCTAATT
T1_32	TCTGACCTATTGGCAGATTCACCAGTTTTTCACACGACCAGTAATA
T1_33	GGTCCGTTTTTTCGTTTTTTTCGTCGCTGGCAGCATTGCCGTTC
T1_34	GCTATTACTAAAATTCGCATTGCTTTAAACAGTT
T1_35	GCCACCGCCACCCTCAGAGAGCCCAATAATACGAGGAAAGTGTATCA
T1_36	CGTCACCAGTACTTTCCGGCACCGCTTCTGGTGCCTGCTGCAA
T1_37	CTTGAGATGGTTTAATTTCAACTTCGAAAGACTAGTAAGAGCAAGAC
T1_38	CCCTCAGCCCCTGCCACTGGTAACAATAATCATTAAACC
T1_39	GCCAGCGGGAGGTGTCCAGCATCAATCCCGTAAAAAAAGCCGCACAG
T1_40	CCAAAAGGCACAGACAGCCCTCATTCGGCCTCAGGAAGATCGCACTC
T1_41	TCCTCATATACCATATCAAAAATCAGTGAGGCCA
T1_42	TATGTGAGCCTTTTTTTTCCCTTGTAGGGCTTAATTGAGAATCG
T1_43	ATGGGTAAAGGTTGTCATAAAGTTGGGCG

T1_44	TCAGTATTTAACATCACCATCACGTTAAAGGGGATTCGCCGTTGTGT
T1_45	ATTTACGAGCATGTAGAGATAAGTTATAAACAATTCTGAACACCGGA
T1_46	GTTGAAATCAATATAAAAGAAACCCTGAGAGCAGCAGGC
T1_47	TGGCTTTTAACGGGGTAGGTTGAGACCCTCAGAACCGCCA
T1_48	GCCTTTAGCGTCACAAAGGATAAAAATTTTTAGAACCCT
T1_49	GTAGATGGGCGCATCGTAACCGTGAACAACTAAAG
T1_50	GCTATTAATTAATAACCTCCGTGTGATAACCCTGAACAGCCTTTA
T1_51	AACACCGCCTGCAATCAGCCGCCACCAG
T1_52	TAAAGAACGTGGACTCCAACGTCAAATGAGTG
T1_53	CTACATTTTTTGTTCCAGTAAGCGTCATACAATCAAGTT
T1_54	CCAAGTTACAAAAGAAATTTCTGCTCATTTGCCGCCAGCACATCCCTTACA
T1_55	CAGTGCCTTGAGTACCCATCCTATTTTGGGGGTGAACCA
T1_56	AACAGTTTCAGCGTAGAAAGGCATCTGCC
T1_57	ATATAAAGTATTTTTCGACAAAAGTTCATTTTTCTTCTGACCTAAA
T1_58	CCAGTAGCACCGTCACCGACTTGAAACGCTAAATAACATA
T1_59	TTTCATCGGCATTTTTTTTCGGACCAGAGCCTTTTTCCACCCAGAA
T1_60	CGGCAAACGCGTACGCCAGAATCACGATTTTAGA
T1_61	AATACCCAACGGAATACAAGATAGCAGCACCG
T1_62	AGCATTAACATCCTTTTTATAACATAAAGCTTTTTTAATCGGCGAG
T1_63	CGCCACCCTCAGAACCGGAATAGCGCAATAATAGGACTGTAGCGCGT
T1_64	CCCCCTGCTTTTTTCAGACGATCCAGCGGCCGGCCAGAATGCGG
T1_65	GTTAGCATATTTCGGGAAACAAACCTGTTTATCAACAATAAACCAAT
T1_66	CAATCAAAAAAATCTAAAGCATAGATTTTCAAACAGA
T1_67	TTTTGTCAGGATATCAGGTCATTGCCTGAGAACCCAGCT
T1_68	AGCCCGAGGAAGATTAGGAACCCATAAATTGTGTCGAAAAAGGGAAC
T1_69	AACGTAGAAAATTTTTACATACATAAAGGCAGTAT
T1_70	GGGGACGACGACAGTAAGTTAGCGGCGGAATCTGCAAAAG
T1_71	GAATTGCGAATAATAATTTGGTAATAGTAAATAGTATTATAG
T1_72	CAAAAATGCATTTTCGTTTGAAATACCGACCGGCTTAGGTATTGAGCG
T1_73	TAACGATCTGAAAATTCTGTATGCATGAGGAAGTTTCCATTAAA
T1_74	CCGAGTAAAAGAGTCTGTCTTGCCTGTCCTCGTTCAGGGCGC
T1_75	GTCTGGAGAACTAGCAGACAGTCATGTAATACTTTTGAGG
T1_76	TTACAAACTGCGGAACAAAGGGACGTTTGCCCAGTTGCAGCAAGCGGT
T1_77	AAAAGATTTTCGAGCTTCAAAGCGCTCCTTTTAAGGAATTGGAAGAAA
T1_78	GATAGAGAGTTGAGATTTAGTTTTTAATACCACATTCAACGGAACA
T1_79	AACTCCAAAGGTGTTTAGCTATATTTTCATTTGGGGCGTTGTACCAA
T1_80	TATCAAAATCATAGGTCTGAGAATTGAGTTACCA
T1_81	AACGAGTATGTCTGGAAGTTTCATGGACGTTGACGAGGCA
T1_82	CAGCCAGCAAACTACAACTTTTTCCTGTAGCATTCAGCCTTTA
T1_83	ACCCGTCGGATTCTCAGCCAGCTGTCTTTACTGATACTAATAGTAGT

T1_84	TGAATATACAGTAACAGTACCAGGTAGCGTTTGCATAAGAGGCTGAG
T1_85	TGCTGTAGCTCATTTTTCATGTTTTAAATAACGAACTA
T1_86	GTGGCATCAATTCACCTCAAATATATATCTTT
T1_87	CTTGCGGGAGGTTTTGAAGCCTTAAAAAGCCTAAAACAGGGAAGCGG
T1_88	AACCACCACCAGGCCACCA
T1_89	ATTGTATCTTTTTGTTTATCAGCTTGCTCGGTTGCGCCGACAAT
T1_90	TTAATGAATCGCAGAGCACCGT
T1_91	ATGTAAATTTTTTCTGATGCAAATCCAATGTTATTTTAGTTAAT
T1_92	TTAATGCCAGAATCGAGAATATAAACGGTGTACCAACTTTGAAAGAGG
T1_93	ATTATCATCATAGTCGGGCAACAGAACTGGCGTGAATTA
T1_94	TTACCAGCGAATTATCACCATTACTATTCACAATTTTTCAAATAAA
T1_95	TTATTTACGAAAGCGTAACTGATAATTTAGAAGTTTTTATTAGACT
T1_96	CCCTCAGTTTTCATACCCTCAGAAAATAACAGTGCCCGCCTGAACAATTACC
T1_97	AATGCGCGAAGAATACGTGGCACAGCACTAAAGCGCGTAA
T1_98	AGAAAAATAATCCGCGCTTAATGGGAAAAACATTAGTAA
T1_99	ACCCTCAGCAGTAATCATTTCATTATACCAGTCATCCATATAAGAGTACC
T1_100	GCTGGCAAGTTGCTTTGACTGCACTCTGTGGT
T1_101	AATAAAGCAAATTGTAAACGCGGCCACCCTC
T1_102	ACGTCAGAGAGAAACAATACTGAGAAGTGTTTTTTTTT
T1_103	CTGAAAAGAAAGCGGAATAAATCAGATAGCGTGAGTAACAAGTTTGA
T1_104	CGAGAAACACCAGAACGAGTAGTACACCAACC
T1_105	ACGTCGCGCAGAGGCGAATACAGGAGGCCGACAAATTAATACATCGG
T1_106	AGAGACTATGACTGAAGACGATAACCTTGCTTCTGTAAATCGTC
T1_107	TACCGAACGAACCCGCCGCCAGCACTCTGAATTTACCTGAATGGCTAT
T1_108	GAGAGGGTCCGTACTCAGGCGAAGCCCTTTTTTTTTAA
T1_109	AGCCGCCGCAGGTCAGACGATTGGCTTTTTTTGACATTAGCAAGGCCGGA
T1_110	ATCCCCGGGTACCCACTACTCGAGGTGCCGTAAAGACAATATTGACGCTC
T1_111	ACTATCGATCCTGATAACCGCAATTTGCGTATTGGGCGCCGCCTGGC
T1_112	CGAACGTGGCGAGATAGGGTTGAGTGTTGTTCTTTTTAGTTTGGAACAAGAG
T1_113	CTTATTACGTGGCAACATATAAAACATATGGTCAGAGAGACGAGCGTC
T1_114	TTTAAATGCAATGCCTGAGTAATGTGTCGGGAGA
T1_115	CCACGGGATTTCATTTGATTTTTTTACCTTTTTTAATAACTAT
T1_116	ATTCTGCGTTTCGCAATTTAATTGAACCAGACCCTCGTTTCCAGAGGG
T1_117	CAAACCCTCTGAATAATGGAGACAACGTCAG
T1_118	CGGTGGTGCCATTAGTGATGAAGGGTAAAGTTAAAGATAGGTC
T1_119	GTATAAAGCCAACAGAATAAACACCGGAATCA
T1_120	AAAATAGCAAAGTCAGAGGAGAAAACTTTTTC
T1_121	GTAAAGTAATTCTGTCTTACCGCGCAATCCAACATTCCAA
T1_122	TCATAGCCCCCTTATCGGATAAGGCGGGGTTTTGCTCA
T1_123	CGATGCTGCTCCGGCCAGAGCACATCAGCAGCTGTTTGGAGCAATTCA

T1_124	AAACATTCAAAAACATGAACGGTCGAACTGACAGACCAG
T1_125	ATAGCTTAGATTAGAAGAGTCCAGTCACG
T1_126	GTCGGGAAACCTGTCGAATAGCCCGAGAAAGGCCACGCTG
T1_127	CGGGTAAAATACGTACGAAGGAATTGGGAATCTACGTTAATAAA
T1_128	ACCCCCAGCGATTATATTACCCAAATTCATCAACAGATGA
T1_129	TGTCAATCATATTTAAACGAGGCACAATTTTTTAGTTGC
T1_130	GCCGAACAAAGTTACCAGTAAGACTCAGAGAGATACTGAACA
T1_131	TCAGGCTGCGCAACTTTTTTTTGGGAAGGGCGATGGCAAAGCGC
T1_132	GTTTAGTAGTTAAATAGCTCAACAAGAATCCTTGAAAACATAGCG
T1_133	GCAGGGAAACCCACTAATGGAGGGTAGCAACGGCTACAGAGGC
T1_134	GCAAAATCCCTTATAAATCAAAAGTGCCAGCTGCA
T1_135	TTTGAGGACTAAACCGCTTTTGCGGGATCGTC
T1_136	GCGCGGGGAGAGGCGGGAATGCCAACGGCTGTCACTTTTTGCGCGCCTGGAG
T1_137	CAGTCCCGGAATTTGTTTTTGAGAGATAGACTTTACGTACAGCG
T1_138	TCCTCATTAGGAGTGTGGGAGGGAGAACGGGTGGCTGTCTTTCCTTAT
T1_139	CCATGTTTACATAGCTATCTTACAGGAAACAATG
T1_140	AGTAGAAGATATTACCGCCAGCCAGAAATGGAGTCACGCTTCGGAACC
T1_141	AATAAACAACATCGAAATTAAT
T1_142	ACTAATGCTAAAACGACGATATATTCGGTCGCAAAAGGCTGAGGCTTT
T1_143	ATAAGGCTCAACATCGCCATATGCGTTATACAAATTCTTACCA
T1_144	CAATGAAACCATCAAGGGTGAGAAGATCTACAAAGGCATTAGAGCCAG
T1_145	AGCCTTTATTTCAACGCAAATTAAGCTAAAGATT
T1_146	CACTAAAACATTTTTTCATCTTTGGACATTTTTCAACCATCGCCCA
T1_147	CACCTTGCCCTAGGGTTAGAACCACGGAACGCAAACGGC
T1_148	TTTCACGTTAAAGAAGAGTGAGTTTTGTCGTCTTTCCAGACGTT
T1_149	TGAGCAAAAGTAGCTCTCACTCCTCAAGAGAAAGTAAGCAGATA
T1_150	GAGAGCCAGCAGCTTTTTAATGTATCTGGTCTTTTTGTTGGGGCGG
T1_151	TTTAATGGAGCCAGTAATAAGAGAGTATTCTAAACAGCCATA
T1_152	AAGACTTCAAATTTTTATCGCGTTTTAAAAGAGGA
T1_153	AGTAACATGGAGCGGATTAGAGCCAGGAAGGTTATCTAAA
T1_154	ACATCGACATAAAAAAGCGGGGTCCAGGAACGACGTGCTT
T1_155	TTTGTATCATCGCCTGATGTACCGTAACAGAGGTGTTTTTATTTCTTAACGA
T1_156	AGTGTACCCCGGTTGCTGCTCCAATTTTCAGGGATAGCA
T1_157	CACCAGAATATCATTTAATTCGACAACTCGTATTAAATCCTAATAGA
T1_158	GCAGACGGTCAATCATTCCGCGACATAATCAGAAATATTTCTCAGAG
T1_159	ATCAAAATACATGAAAGTATATATCATTTCA
T1_160	AGAACGCGAGGCGTTTTAGCGAACGGCAGAGG
T1_161	TGTTACTTAGCCGCATTAGACGGAAGACACCAAAGAACT
T1_162	CGCTCACTGCTTTTTCGCTTTCCACGGGTTTTTCGTTTTCACGGTC
T1_163	TATTTTGCACCTTCATCAAGAGTAGCGCATAGGCTGGCTG

T1_164	TAGTCTTTATTAAAAAAATCGTCTTTGCAACACGCCGCTAAGAATCAG
T1_165	CAGTTCCTGATTATCTTTTTCTTCGTGGTGCTGGTCTGG
T1_166	AATAAAGACGTGGGAATGCCGGACTTGATGTTTAGACTG
T1_167	TCACCCAAGAGAACAAGCAAGCCGAAGTACCGCACTCATC
T1_168	AAAAACCGTCTATCAGGGCGATGGCCGAGCTCCCACCACACCCGATC
T1_169	ATACCGGGCACATTAATTGCGTTGTCCACTATGGAAAGCCGG
T1_170	TCAAGTTTGGCATGATAAGGAAACAGAGCAAGTTTAGTACACGTTGT
T1_171	GCTAATGCAGAACGCGCATCAAGAAAACACAAGACTTTTTAAGAACGCGGTA
T1_172	AGGATTAGACATTATTAATTTTAAAAGTTTGGATTTTAA
T1_173	GTTAACGGCGCGCTCTCTTTTTTCGCACTCAATCCGCCGGGCGC
T1_174	TACATTTAACAAACGGATAACCTCACCGGAAACAAAGCGGATC
T1_175	TAAGTTTTGATGATACAAAGCCAGAATGGAAAGCGCAGTTTGACAGG
T1_176	CGCCACCCTCAGAAGCAGTTGGGTAACGCCAGGGTTTTCCAATAGTGAATT
T1_177	ATTGCAGGCATCAGAGCCGGGTCTTCCACACAACATACGAGCCG
T1_178	AGATACATAACCAAAATAGACAGCTTGATACC
T1_179	GAAATTGTTATCCAGCCTCCTCACAGTTGAGG
T1_180	GAATTCGTCGTCCGTGGCTCACAAACTGTTGCCCTGCGGCTGGTA
T1_181	AATCGTAACAAACAAGGGAGAGGGTAGCTATTTTTGAGAAGGCCGGA
T1_182	GCCTTGCTGGTTTTTTATATCCAGAACAAACTCAA
T1_183	CAGAAAACGAGAATGACCTTGCATCAAAGTTTTGACCAGACG
T1_184	ATTCTGGCCAACTTTTTGAGATAGAACCCTATTTAGAG
T1_185	GAAGCATAAAGTGGGGTGCCTAAGGGCGCTAAAGGGAGCCCCCG
T1_186	AGCATCGGGTTAAAGGGACTTTTTGGATTTTGCTAAACAACTTTC
T1_187	CATTCGCCATGTCATAAATATTCAGCCCAATACT
T1_188	CAAAATCAAACGTCACAGAAAATTGAAACGCAGAGAATTAAACCCACA
T1_189	GGAATTGGTCAATAGATAATACATTTTTTTGAGGGCCCTAAAACATCGCC
T1_190	CGCATAACAAGAGGCAAAAGAATATGCCCTGAACATTATTAC
T1_191	TGCAACTAGTCATTTTTGCGGATGGTTTTTTTAGAGCTTAATTGCT
T1_192	AGGTAGAAAGATCAACGTAACAAAGCTGCTCATTTTTTCAGTGAATAAGGCT
T1_193	GCTGATTGCCCTTCACCAGGGTGGAGATGATGTTATACTT
T1_194	TTCTTCGAATCCTGTAAAGCATGGTCATAGCTGTTTCCTGTGT
T1_195	TGATTGCTTTGAATAGTACCTTTCCGTTGTAGCCACAGTGCCACGCT
T1_196	GGTTGCGGTATGATGCCGGGTCGTGCCTGGTACTATGGTGTAGCG
T1_197	TGGGTTATATGGAAACAGTACATACCAAGCTTTCAGAGGTGGAGCCG

► For the Tb 3D origamis (“Toblerone”-like structures, Fig. 3D, Supplementary Fig. 18), we created the design using CaDNAno software (v.2.0) using a 7560-nt M13 scaffold named p7560, and the following staple strands:

Tb_01	AGAAAGTCAGCTTGCTTTGTAAGCAACGACCTCCTGGAGAAT
Tb_02	TGCTTTTCAACAACTAACGGAACTCATAAATAAAA
Tb_03	AGCACTACTAAAGGAATTTATAAATCGTTGTGAGT
Tb_04	GACGTTTGCAAGCTGTTG
Tb_05	ATCTTTTGCCGCCAGCCATTTGAAATTAGTTTACCCGGAATA
Tb_06	GAACTACATACGACACCAAGCGCCAAAGACAA
Tb_07	AGCTAGTAGTTCATTTGTTTC
Tb_08	AAGAATTTGAAATAGAACAAAGTTACCAATACCCACAG
Tb_09	GGGATTTTATCGATTTACCGACAGGAACATTAACC
Tb_10	GTTTAGCTTAGGTTTTATTTCTTCAAAAGGGTGTCAAAAATCAGCGGGAG
Tb_11	AACCCCCAGCGATTGAACGAGGCGGAGAAGACGGCAGA
Tb_12	GCGCGAAGAATACAACTTTTT
Tb_13	GCGGAATCGAAATAAAGAATCTAAGAACGCACC
Tb_14	CAGAGCCACAAACTATAGTTAGCGTAAGGGATTAAGTGCTAA
Tb_15	GGATACTTCAATACCTTTGGATGGCATCAA
Tb_16	GATGTGCGTAAAACCAGGGTG
Tb_17	GATATAGGTTTTAGGATAAGTTGTCCAGATTTACCTTGGCCT
Tb_18	TAACGCCAATAGCGGGGTAATAGTAAAAAACAGTTGGAA
Tb_19	AGTTGCGAGGCTAAGTTTTTAA
Tb_20	CGAGTAAACGCCATTACCAAAGGATAAA
Tb_21	CCGTGCAAAGCCTCAAAGAAAACAAAACCTCAAATATCGTAA
Tb_22	CCAACGCTTAGTTGTTAACAATATAAAGGTTAATTCCACCACCCT
Tb_23	ACATGAGAGAGAGCTGATCAATTCCGCT
Tb_24	CGGTGTAATCTTGACACCGAGGTCACCAACACCGGAATCTGGC
Tb_25	ATGATATAACCCTACTAAAAAATTCGGTTGCAA
Tb_26	CCAAGAATTTCATCCAAATCA
Tb_27	AAACCAAAAAAGGAGAGATTT
Tb_28	AAGGGCGAAAAGAAACGCAAAATAAAGGATAATTAGGGCT
Tb_29	CGCTGCCGACAGGCTCCAAAAGGAATAATAAGTTTGCCA
Tb_30	GGAAGGGGGCACCGGTCCTTAGCCAAAACCGA
Tb_31	CCAAATCGACCTTCTTAGAGCCATTCCATAGATTTATTT
Tb_32	GAAACGACTTGACCCTCAGCCGCCCA
Tb_33	CATATTCAATCCTGAAATTATTTGC
Tb_34	TTTGCAAAATACAAAGATCGAAGTTTTGGACCA
Tb_35	GGGCGTAGCGGGGTTTTCAGCGGAGTGTTGGTGTAGCGTATT
Tb_36	GTTTGAGTAACAGTGATTAGGATAACTAATGGTCAATAACCT
Tb_37	AGATTCAACGTTAACTCAAATGAAGCAACAGGAGTCCTATTT
Tb_38	CCAGCCAGTATCGGCGGATTGTCGGATT
Tb_39	TGGCTTTAGGAGCACTAACAACAGAAACCCTCAATCAATGCT

Tb_40	CATAATTAATAAGAGTCATACCAATTTT
Tb_41	GAAATTGCGTTGCGGCTTTCCTCGGCAACAAAAGA
Tb_42	GAGGGAAGGTAAATAAGGCGTTATTTTATACCGACAAAAGAGATG
Tb_43	CCATCTCGAGGTGAATTTCTTAAACGGCCGCTCAACTAATGTA
Tb_44	TCAATATCTGATTAAACGTTA
Tb_45	TATCTATACCTCATAGCTCATTTGTAATAC
Tb_46	GGATTATAGCGGAATTGAGTA
Tb_47	GGAAGCAAGCCTGGGGCGCTGGCAAGTGGAAAGGA
Tb_48	CAGCTACAACCTTTTGAGGAAGCAACTTTTTCAAATATAAATA
Tb_49	TACGCCAAGCCACTTAATGCTTTCTCAGATTGT
Tb_50	ATTAGAAGATGATGCTTTGAAATA
Tb_51	ATACGTTAATTAATTCGCATTAAAGTTGGTG
Tb_52	CATGAGAGGGTACCGAACCTGGCTAACGTACGAGTA
Tb_53	CGTTTACGTAAGAGGGTAGAA
Tb_54	ATAGGTCGCTGATTGCGAACCAATCGCCCGAAAAA
Tb_55	GCTCGGAAGCGAACTGGCAGTCAGCCACAT
Tb_56	CATGGTCATAGCTCCACAGACTCAACAGTTTGCTCAGAGAAGGCCC
Tb_57	CCCAAAATTAAAATAAATCACTGTGTAGAAGAACGTGGACT
Tb_58	CGGAACCAAACATGAAAGAACGAA
Tb_59	CTCTTCGCTGATAAATTGTGTCGAAATCCTTTGAAACAGCATAGTT
Tb_60	TTAGGCAAAGGACGACAGCTT
Tb_61	GGATATAGATGGTTTAATTTTATCCTGTATCCCAATAGAACC
Tb_62	GGACAACCCGACCGTAATGGGATAAGGGGACAATT
Tb_63	ACTCATCTTTGATAACCGAGGAATGAGGGGCTTACGCAATAA
Tb_64	AGAATCATAGCGTCAAAATCA
Tb_65	TGACCCTGACGAGAAACATTTTAAAAACTCCCTTCAAAATGCTGACTCC
Tb_66	CTCATATATATTCGCCTGATTGAAACAAACATCGACCTTCCTGTTAATAG
Tb_67	GATGTTTTAAGTCTTCTGAGTTTGGGTCACTGTGAG
Tb_68	AATACGAACCTATCAAGATAACGAGAAAATAA
Tb_69	GAATTAATTACAATATACGCTATTCACGCAAGGTACGCGATTAAA
Tb_70	AAATCCCAGCTGCCGGCCAACTACGTGTTCACATAGGGGCCTG ACTCT
Tb_71	AGGAGAACAATTGATTAGTAATAATGTC
Tb_72	GGACATTTATTTACTTAACACATCACCTTATC
Tb_73	AGTCTTTCAGACAAATAAAAG
Tb_74	CAGGTCAAACTAGCGAAAAGC
Tb_75	CCAACGTAATGCCTTAGAACC
Tb_76	GTACGGGGTCAGTGCCTTGGAACAAGAGTCC
Tb_77	AGATCATACATAGGTTGAGGCAGGTGCCGCCA
Tb_78	TTTTTATCGGGTATATCCTAATTTACGAAAC

Tb_79	GAGATTTCTAAAACCCATTAA
Tb_80	GTTTATGAGTAGAACTCTTCGTCGGTGCGGCCCTG
Tb_81	TAGATGGGCGCATCCCAGCTTTCATCAACGTCTGGGCATAAA
Tb_82	GAGCCCCTGGTACTGGTAATAAACTATTAATTCGAGAACA
Tb_83	ATAGCCCTGAGTGTTTCTTTTCTCCGGCTATA
Tb_84	TTTTGACCAACATGAAAACGGAGATGGATCGCACT
Tb_85	ACAGCCATTTTTGTAACAGTACTAGAAAGATAAATATT
Tb_86	CCGTCTAAAACAATAACGGTTTAAATGCCAAAGGGTTCACCGCACGCTGGCAG GCGA
Tb_87	ACCAGTACCACCGTACTC
Tb_88	CTCCGTGGGGCTTGATAAGAACAAACGGCCTCAGGTTTCATACAGGC
Tb_89	AGGAGGTGGCGGATTTGCTAAATTGCGAGCCTTTATACCGAT
Tb_90	GCATAATGCAGTACGGGATACATTAAA
Tb_91	GTCGTCTTAGTAAAATATAAGTACCACCCAGAGTAGCCCGGA
Tb_92	CAAAGCGCAGTCTCTGAACGACGAAAGAGAACGC
Tb_93	AAAACAGGTCAGTAATTGGCACAAC
Tb_94	TCAGGGACACTGAGAAGTTTT
Tb_95	TTAATGCGCTGAGATGCAAATACCATTATGT
Tb_96	ATCGTAATTGCCTGGAGAGGGTAGCTATGACAGTCTCGCATTAATA
Tb_97	ATAGGTGAGGGTTGTGAATTTCACGTTGAAAAAAAATGACAA
Tb_98	AAAAGCTTGAATTGTATCGGTTTAGAACAACTTCGCAATATG
Tb_99	GCATTGACATTCTGTATTAGGGGCTTTTGATGATAAGC
Tb_100	TCAACTCCCCGGTTGTGTTAAAAAAGA
Tb_101	TTCCACGAGCTATCGTGCTGTTTGATGGTGGTTCCGAAAAGTCGGGGCG
Tb_102	ACTTCTTATTACCGCCTCGCTTACGCAGCCATTGGATTCAC
Tb_103	CAGTCAAGCAAAGCGCAGGCGAGGTGCATTCGCCA
Tb_104	AACATACGCACGCCCATACATTCAACCTCCAAAAATCGATTGAGG
Tb_105	GAAACAAGAGTTAAGAACACCCTGAACAATAAAAAAAA
Tb_106	GACGAGCACAGGGCCACGCTGCGCGTAAGAGAAAGCTGAGAAAAGA
Tb_107	GAATAACCTTGGCAAAAGGTGCTTCTGTCAAAATC
Tb_108	GCGGAGACGCTGAGAGAAGGGTGAGATGTTGCAGCAAGCGGTCCCTGGCCGG CA
Tb_109	GCAAAAACAAAGTGCTCCATGTTACAAGGGAAGCAACGGGGT
Tb_110	AGCGCGTTAATCAGAAGGCCG
Tb_111	AGTGCCAGCTGGCGTGCGGGC
Tb_112	ACTATTAGTAAAGAAACGCAAAACA
Tb_113	AGGCATACAGACGAGACTGGA
Tb_114	TGATATTACCAGAATCATCTTACCGTGTAAGCCTGTAG
Tb_115	CCCAAAATTAAAATAAATCACTGTGTAGAAGAACGTGGACT
Tb_116	TTAATTTTATTAAAAATTGCGTCGGGAGTCAGGGCGCCGTAA
Tb_117	ACGGGTAGCGAAAGAGAGGACTGTACAGTCAG

Tb_118	GTAAATTATTGTGATAGAGAGATATCGCGCAAGACAAAGTATCGTC
Tb_119	CCGGAACACCGGAAGCAAAATCAGACGTTTCCACCAGTAGCA
Tb_120	AGGCCCAGAATCGAAGGGACGATTTATCGAGGTGATGGCC
Tb_121	AAGCCTGGGTTATATCCAGGGTGGTTTTGTTCCA
Tb_122	GAATTATGCGACCAGAAACAAAGCTGCTCATACCAG
Tb_123	GGAAAAACGATTATTATTAATCTTA
Tb_124	TTAATTGTTATCCGCTCGAATTCGTAAT
Tb_125	AGTTTACAAACGTTTAGTAAAGCCATG
Tb_126	AGGAATAGACGTTGCAGAAAATACCCTGGTTTTAATAAACAG
Tb_127	CAGTGGGCACGAATATAAATCAGGGCGCCTACCT
Tb_128	CTGTAGCAGAACCGCGCCACC
Tb_129	AGAATACAATTCTGCGCGAGAACCGAAAGTGCATCATTCATTGCAATACT
Tb_130	GTCCATCACTTGCTGGTAATATCCAAAAACGCATAAATCATT
Tb_131	CCATTACAGAGCCACCGCCTCCAGAGCCCAGACGAGTTCCAG
Tb_132	CGTGGCAAATGCGCAGAAGAT
Tb_133	TTCAGGCAAACCAGTTCATGCAATCTATCCTGGAGTTGAATCGGAGCAAT
Tb_134	AATAAATTAAATATGTTTGCGGGATCGCTTATGACAACATCC
Tb_135	TAGCGTCAATCCCCTAAAACGTTTAATCGGGCTTGTCATTAC
Tb_136	ACGTAAAATCTAGCTGATAAATTATATG
Tb_137	GTAAATTCAACCGTCAGAAATAAAGATCCTTTGCCTTAAATT
Tb_138	CAACCATTAACCGAGTTGATTTTTTTGCAAT
Tb_139	AATGAGTACAACATGAGGATCCCCGGGCTTAAGCGC
Tb_140	TAATTGAAAAAATAAGGCGCATTATTAACTGCCCAAT
Tb_141	ATATCGACACTCCATAACCCT
Tb_142	AAAATTTTGACAGGAACGGTA
Tb_143	GGAACAACTTAGCCCTCACAACGC
Tb_144	CTCAGAACCTGCCCCTCACGC
Tb_145	AATGACCCTTTTAACCAAATTTTATAATCAATGTCAA
Tb_146	TCAAGTACCATTAGTACCCACCCT
Tb_147	TCATATGTAAATGCAGATACA
Tb_148	CAGACATAATCAGACTGT
Tb_149	GTTGTCTGACGCATGTGCTTGAAAGACGACGAGCC
Tb_150	TAAGCGTTAGTAAAGTAATTCCCTGAACATATCCCTAAACCA
Tb_151	ACATAGATTTCATATCAATTGTTT
Tb_152	AGTTGGGGTTTTCCGTGGTTGTGACACGACCAGTATATTTTT
Tb_153	GACAATCATTTAGCCGATACCAA
Tb_154	GAGTATCACCCTCATTT
Tb_155	TAAGCGAACCAGGTCTTCGAGAATCCAGAGGAGAGGCT
Tb_156	CTGGTGCAGGGCGGAACGGAAGTTTGAAAGAG
Tb_157	ATTGTAATAAAGGGTTGCTTT

Tb_158	GACCAGGTAACGATGGCTATT
Tb_159	CACCCTCATTGTTTCCTGTGT
Tb_160	GGTGCTGAATGCATAGGTGACCAACGCGACCTACAACG
Tb_161	AAGGCCGGATTTTGAGAGGCTAT
Tb_162	TTTAGCATTAATGTCCCGTGCTGAGAGAAGCGACGGCC
Tb_163	CCTAACAGTACTCATGGAATGGATCTGGCCAAAGAATA
Tb_164	GCTAAATAAGCAATATCTGCCGTGCCGGTGCGCAAGCGATTA
Tb_165	AGTACCGGAACAAGATCCGGTATAACATTATTACACAACACT
Tb_166	TCCCGATCGGAAAGGGG
Tb_167	AATTTTGAGTAACATCAAATGCCGAGAGTC
Tb_168	TTTTCTGTATCGATCTATTTCGTC
Tb_169	GTGGAGCCCCAGAAAGCTAGCGGTGCGTACT
Tb_170	CAACAACGCTCTTAACGTGAATAACAAGTCAGAACCCAC
Tb_171	CAACTCACCCTCAGCAAAATACGACGAAGG
Tb_172	AAAATGGCATGGATAGCCGCAATAG
Tb_173	CGAGTATAACATATATTCCTACAGAACTAAAGCTAAAAC
Tb_174	GACGGGGAATTCATTAGCTAGCGAC
Tb_175	ACCGTAATAGCAAGCCCAATA
Tb_176	TAACGGAGAAGGAAAGAGCAA
Tb_177	TGGAGCATCGATGAAGATTGTATAAGCAAATATCGTCAGATG
Tb_178	TCTGCACGACCTTCTAACAGTCAC
Tb_179	TTTTAACCACCAGTGGGAGAGAAACCTGACTCACA
Tb_180	ATGAGAAAACAAGGCAATTCA
Tb_181	GGGCCTTAGTTTAACCCATGT
Tb_182	TTCAGGATATTACCAAATCTTCAGTTATTACG
Tb_183	ATCGTTTTATAGTCAGCTTTATGTTTACGATAA
Tb_184	TAACAAAAGAGCCGTCACAACTCGTAAAAGTTTATCAT
Tb_185	TCCAACAGGGTACCGAGCTCACAA
Tb_186	CACCAACGTATCATCGCCTAT
Tb_187	GCGGGCGCTTGAATATTAAAGGGTGCCT
Tb_188	AATCGCCATATCTATTTTGCGAGGCAAGGCTTCAAGCCG

► For the multiple folding pathway characterization (Fig. 4, Supplementary movies 1–4), the Λ DNA origami design corresponded to the triangle where only two sides were formed. In our case, we arbitrarily chose to fold the B and C sides (see Supplementary Fig. 19). The subsequent folding of the A side produced a fully formed triangle (or Δ origami). Additionally, we replaced four staples with staples containing the same sequence followed by a TT spacer and an additional single strand part to hang a complementary strand modified with cholesterol at the 5' position. This results in

cholesterol-modified staple-strands exposing a cholesterol moiety in the B and C sides to facilitate the nanostructure's adsorption on mica-supported lipid bilayer. The positions of the modified staples are shown in Supplementary Fig. 19. The sequences of the modified staples are (red: in-origami, blue: TT spacer; green: protruding part hanging the cholesterol-modified strand):

B-side:

- B25 TAAAGCTATATAACAGTTGATTCCCATTTTTG-TT-CTTCGTGCTACGCATCTCTGTA

- B51 TAGTTGCGAATTTTTTCACGTTGATCATAGTT-TT- CTTCGTGCTACGCATCTCTGTA

C-side:

- C25 TTGAGGATGGTCAGTATTAACACCTTGAATGG-TT- CTTCGTGCTACGCATCTCTGTA

- C51 AGTTGGGTCAAAGCGCCATTCGCCCCGTAATG-TT- CTTCGTGCTACGCATCTCTGTA

Cholesterol-modified strand binding to the modified origami staples:

Chol-TACAGAGATGCGTAGCACGAAG

2) Supplementary Figures and Texts

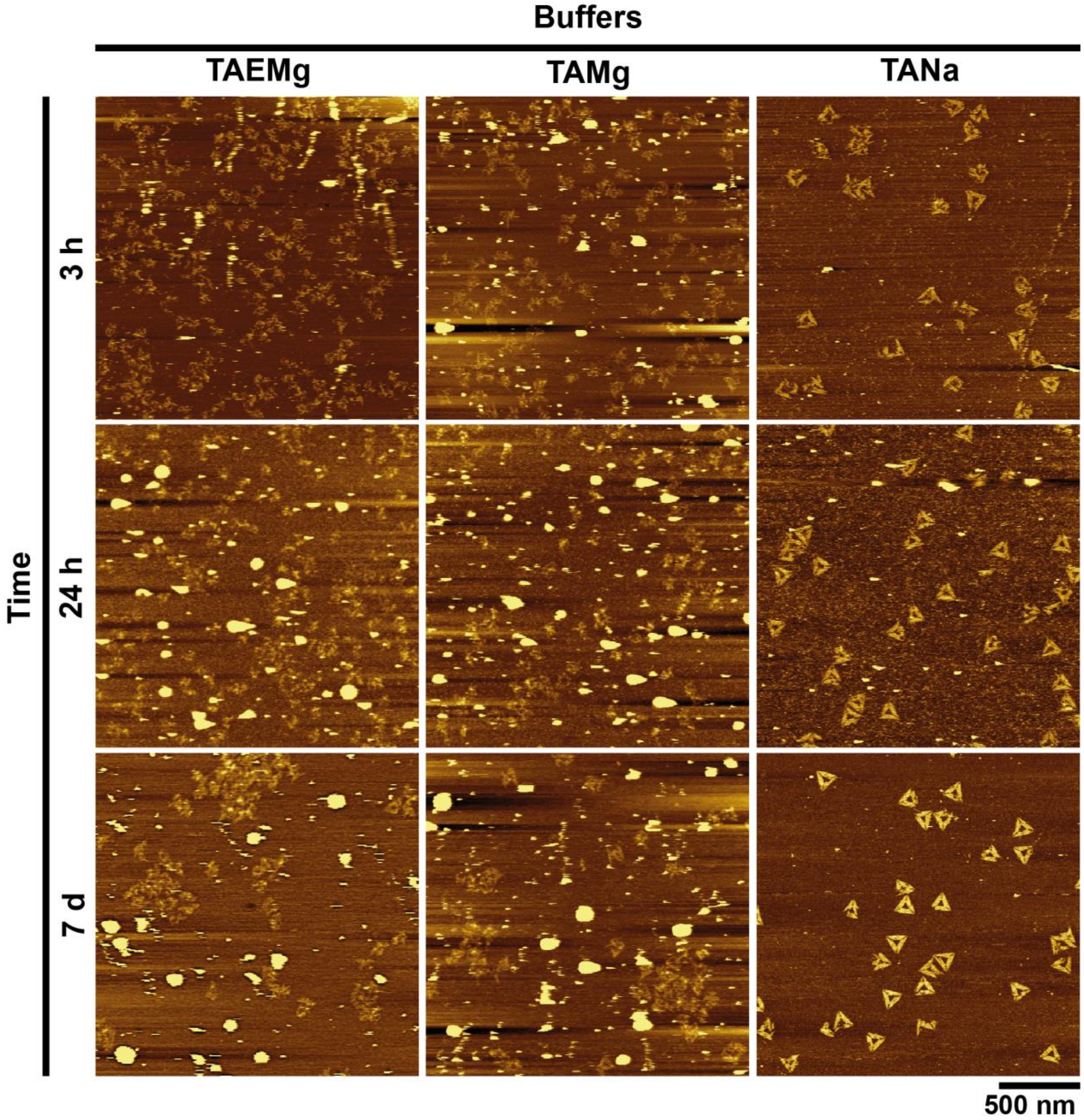

Supplementary Fig. 1. AFM images of structures obtained by isothermal self-assembly at 25 °C of an origami mix coding for sharp triangles (40× excess of staples), as a function of incubation time (vertical axis) and for various buffers (horizontal axis): TAEMg (Trizma base 40 mM, acetic acid 20 mM, EDTA 1 mM, $MgCl_2$ 12.5 mM), TAMg (Trizma base 40 mM, acetic acid 20 mM, $MgCl_2$ 12.5 mM), TANa (Trizma base 40 mM, acetic acid 20 mM) supplemented with 100 mM NaCl. [M13] = 1 nM; each staple concentration is 40 nM; no staple purification before AFM imaging.

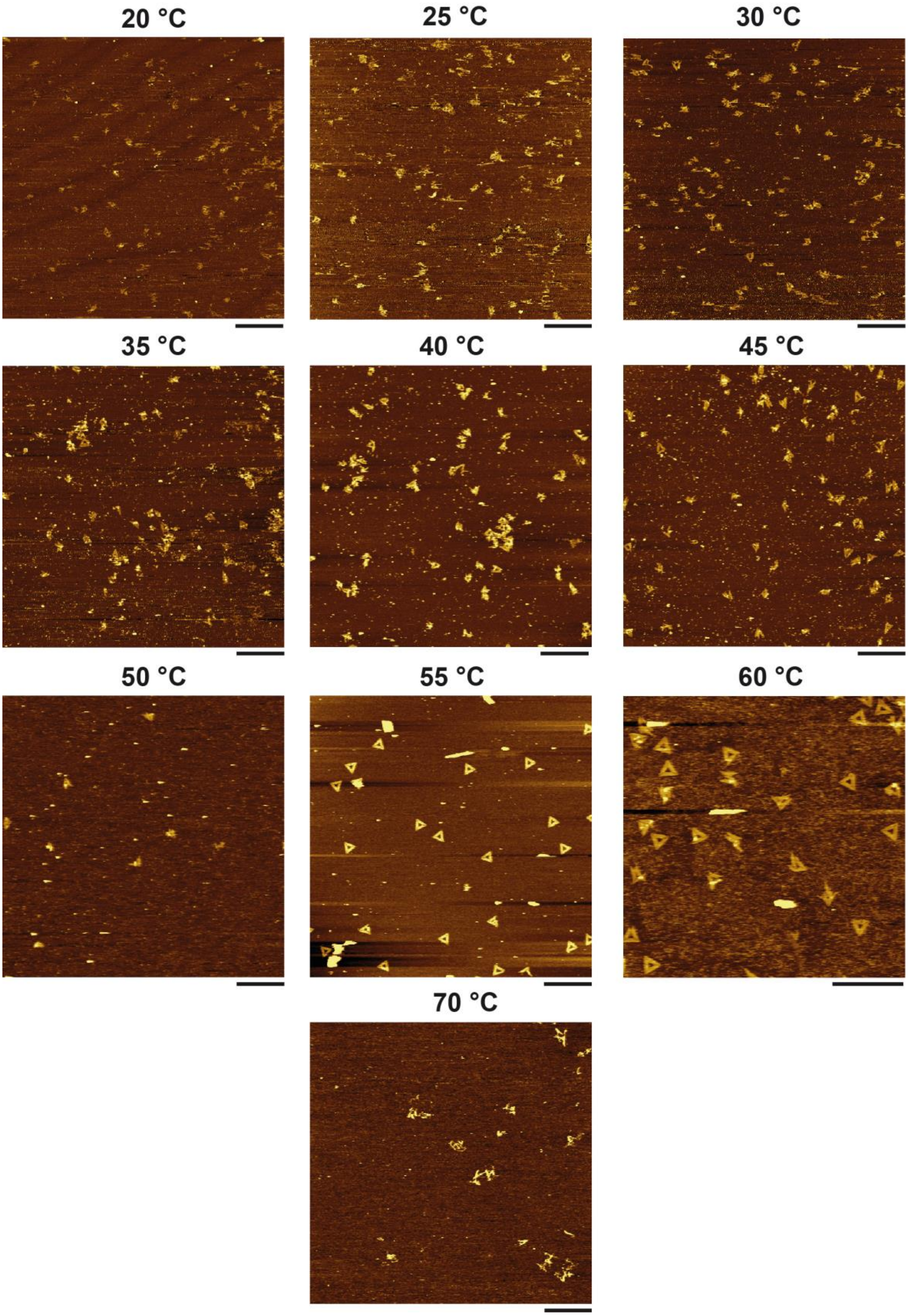

Supplementary Fig. 2. AFM images of the structures obtained by isothermal self-assembly of an origami mix coding for sharp triangles (40× excess of staples) in TAECa buffer (Trizma base 40 mM, acetic acid 20 mM, EDTA 1 mM, $CaCl_2$ 12.5 mM) after 24 h incubation at various fixed temperatures. [M13] = 1 nM; each staple concentration is 40 nM; no staple purification before AFM imaging. All scale bars are 500 nm.

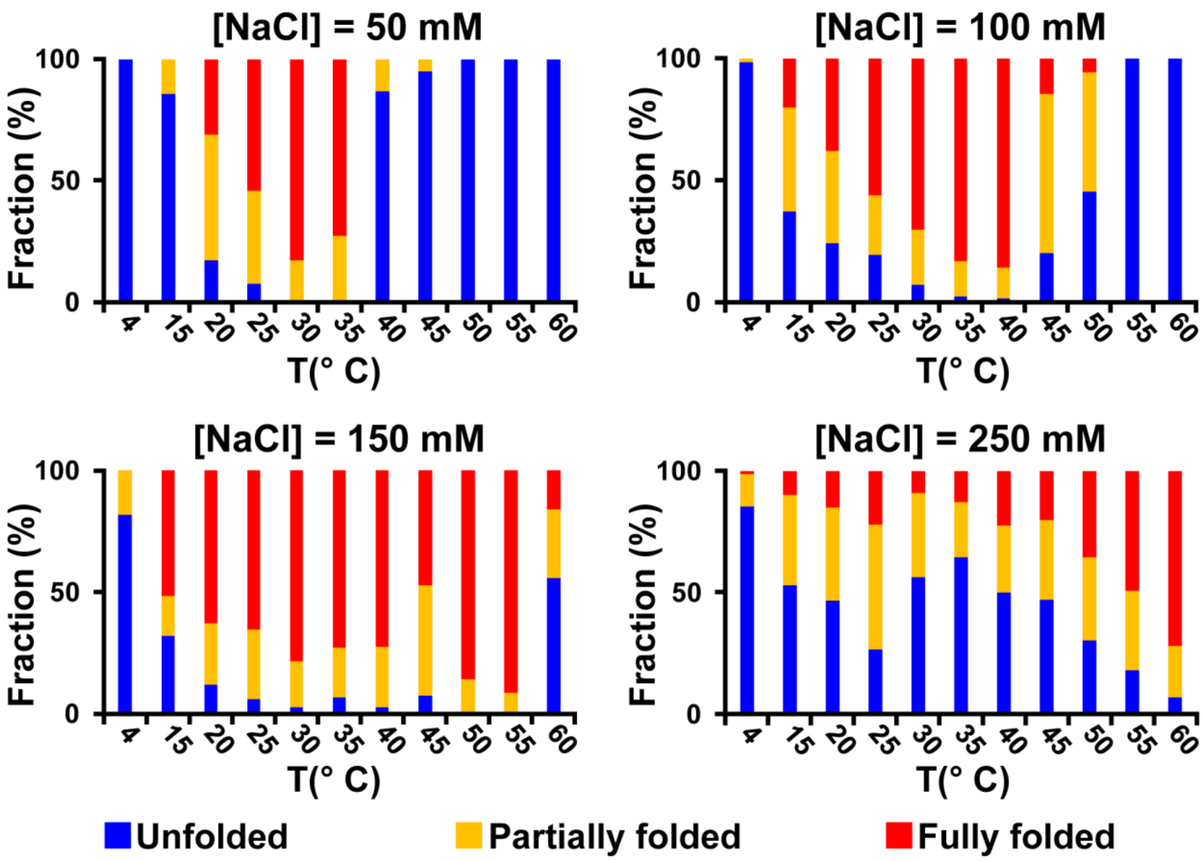

Supplementary Fig. 3. Fraction of unfolded (blue), partially (yellow) and fully (red) folded origamis after 24 hours of isothermal self-assembly in TANa buffer with a set of staples coding for sharp triangles (40× excess of staples), for various incubation temperatures and NaCl concentrations. [M13] = 1 nM; each staple concentration is 40 nM; no staple purification before AFM imaging. The number *n* of analyzed objects for each condition is provided in Supplementary Table 1.

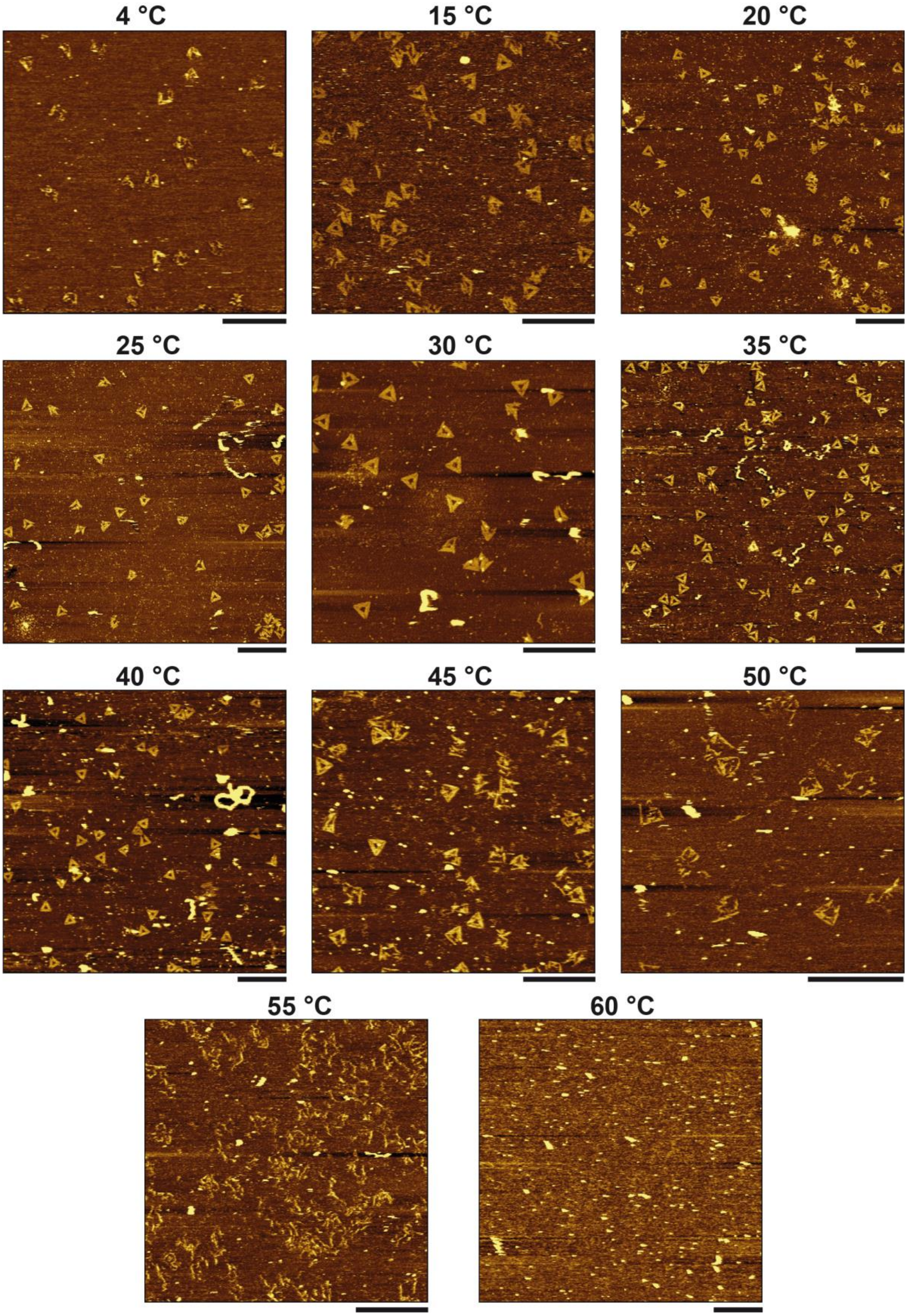

Supplementary Fig. 4. AFM images of the structures obtained by isothermal self-assembly of an origami mix coding for sharp triangles (40× excess of staples) in TANa buffer ([NaCl] = 100 mM) after 24 h incubation at various fixed temperatures. [M13] = 1 nM; each staple concentration is 40 nM; no staple purification before AFM imaging. All scale bars are 500 nm.

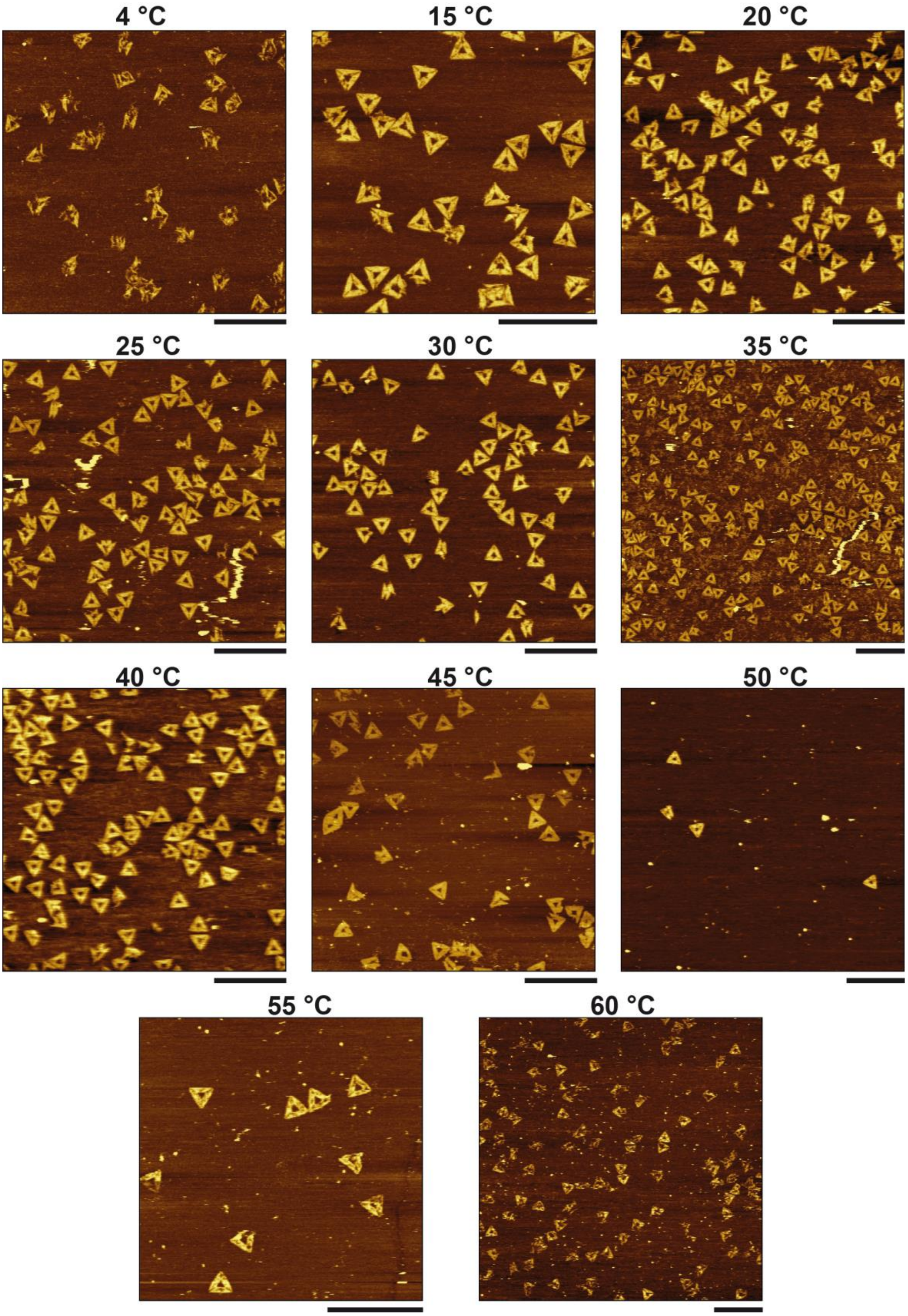

Supplementary Fig. 5. AFM images of the structures obtained by isothermal self-assembly of an origami mix coding for sharp triangles (40× excess of staples) in TANa buffer ([NaCl] = 150 mM) after 24 h incubation at various fixed temperatures. [M13] = 1 nM; each staple concentration is 40 nM; no staple purification before AFM imaging. All scale bars are 500 nm.

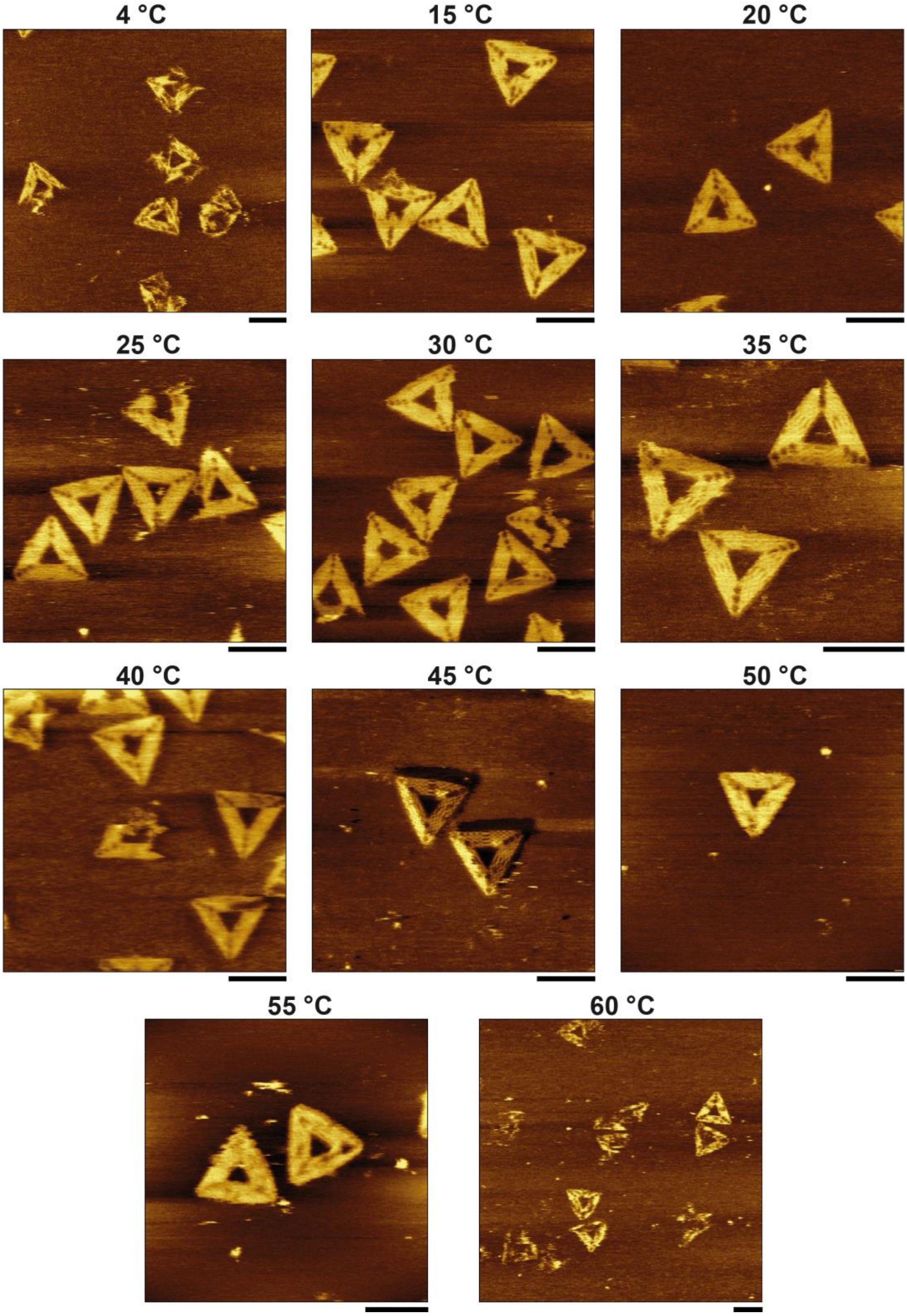

Supplementary Fig. 6. AFM images (close-up) of the structures obtained by isothermal self-assembly of an origami mix coding for sharp triangles (40× excess of staples) in TANa buffer ([NaCl] = 150 mM) after 24 h incubation at various fixed temperatures. [M13] = 1 nM; each staple concentration is 40 nM; no staple purification before AFM imaging. All scale bars are 100 nm.

Supplementary Text 1. Isothermal self-assembly with other ionic compositions

We found that successful isothermal assembly of origamis at room temperature was hindered by the presence of divalent cations such as Mg^{2+} and Ca^{2+} (Supplementary Figs. 1-2) but was rendered possible in TANa, a tris-acetate buffer supplemented with the monovalent salt NaCl (Fig. 1, Supplementary Figs 3-6). Replacing NaCl with LiCl (100 mM) resulted in the isothermal self-assembly of properly formed origamis in a similar range of temperature (15 – 50 °C, Supplementary Fig. 7). Similarly, keeping $[Na^+]$ = 100 mM but replacing Cl^- with an acetate counter-ion led to successful isothermal assembly at 25 °C (Supplementary Fig. 8). This shows that the main role of NaCl is to adjust the ionic strength rather than a specific chemical interaction with DNA and opens the possibility to generalize the isothermal self-assembly principle in a potentially broad variety of monovalent saline solutions.

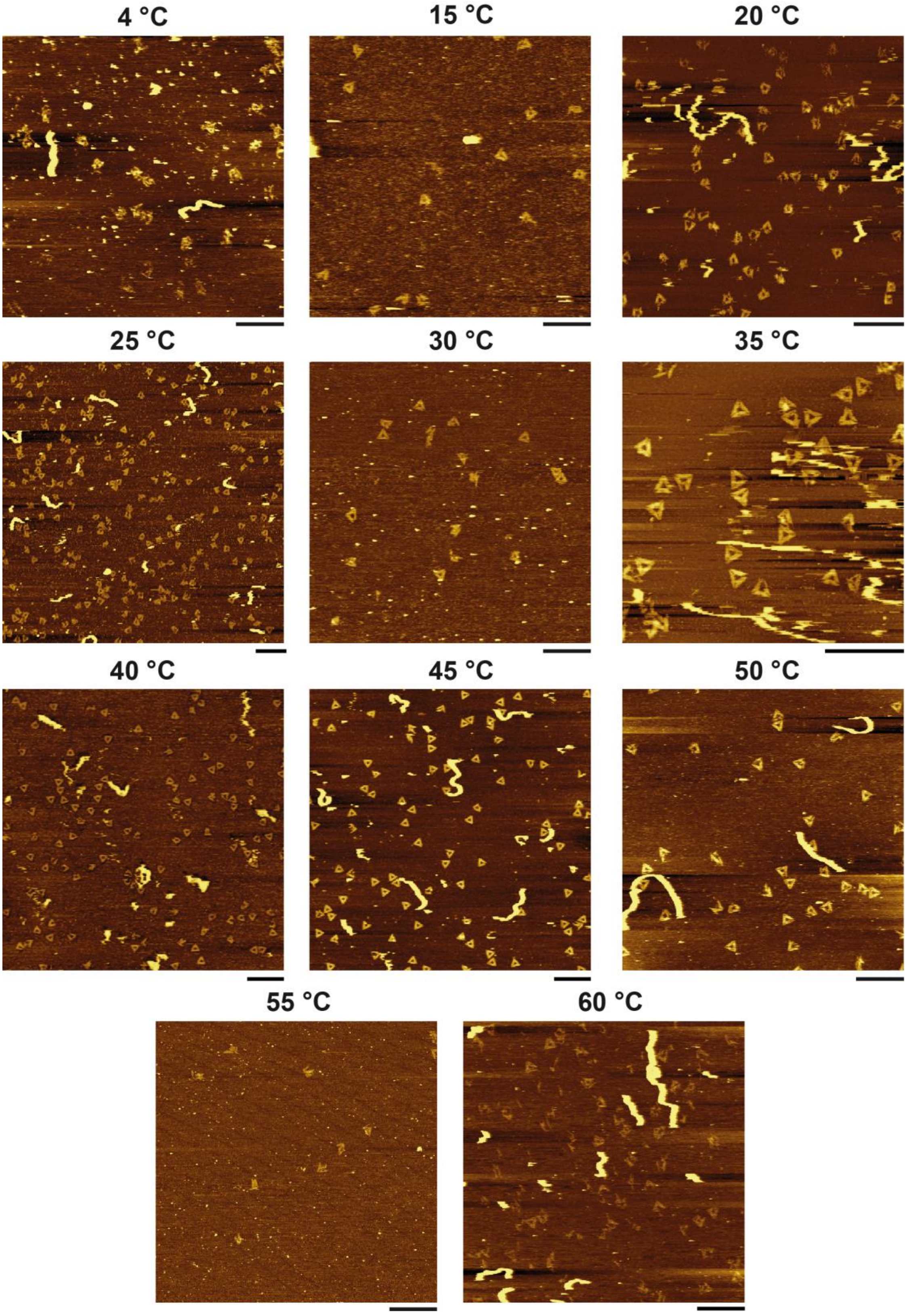

Supplementary Fig. 7. AFM images of the structures obtained by isothermal self-assembly of an origami mix coding for sharp triangles (40× excess of staples) in TALi buffer (Trizma base 40 mM, acetic acid 20 mM, LiCl 100 mM)) after 24 h incubation at various fixed temperatures. [M13] = 1 nM; each staple concentration is 40 nM; no staple purification before AFM imaging. All scale bars are 500 nm.

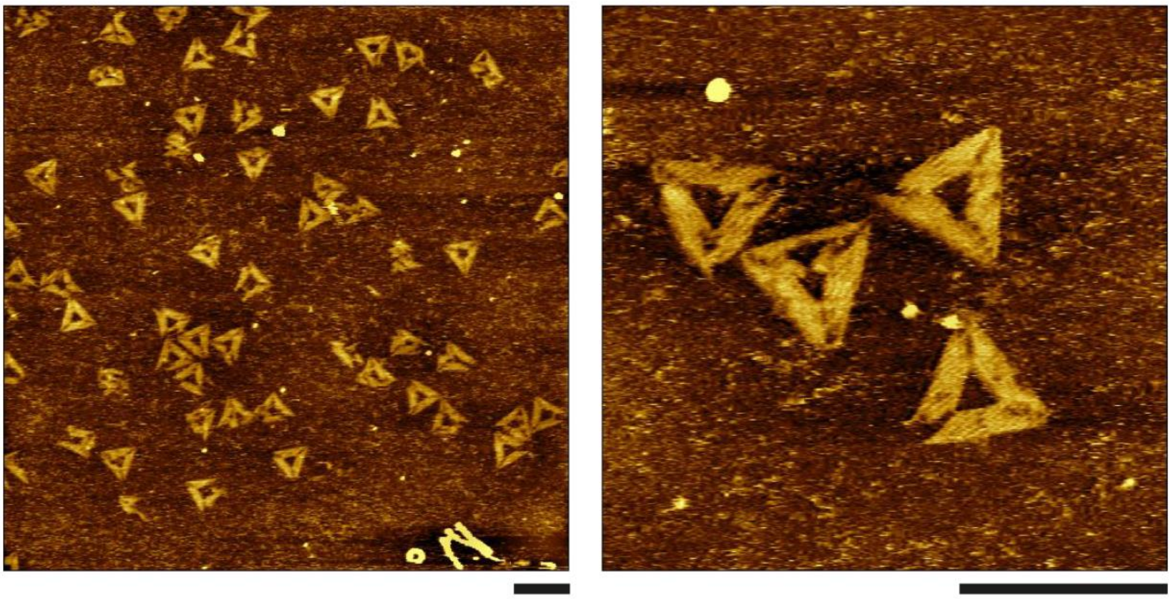

Supplementary Fig. 8. AFM images of the structures obtained after 24 h of isothermal self-assembly at 25 °C of an origami mix coding for sharp triangles (40× excess of staples) in TA buffer (Trizma base 40 mM, acetic acid 20 mM) supplemented with 100 mM of sodium acetate. [M13] = 1 nM; each staple concentration is 40 nM; no staple purification before AFM imaging. All scale bars are 200 nm.

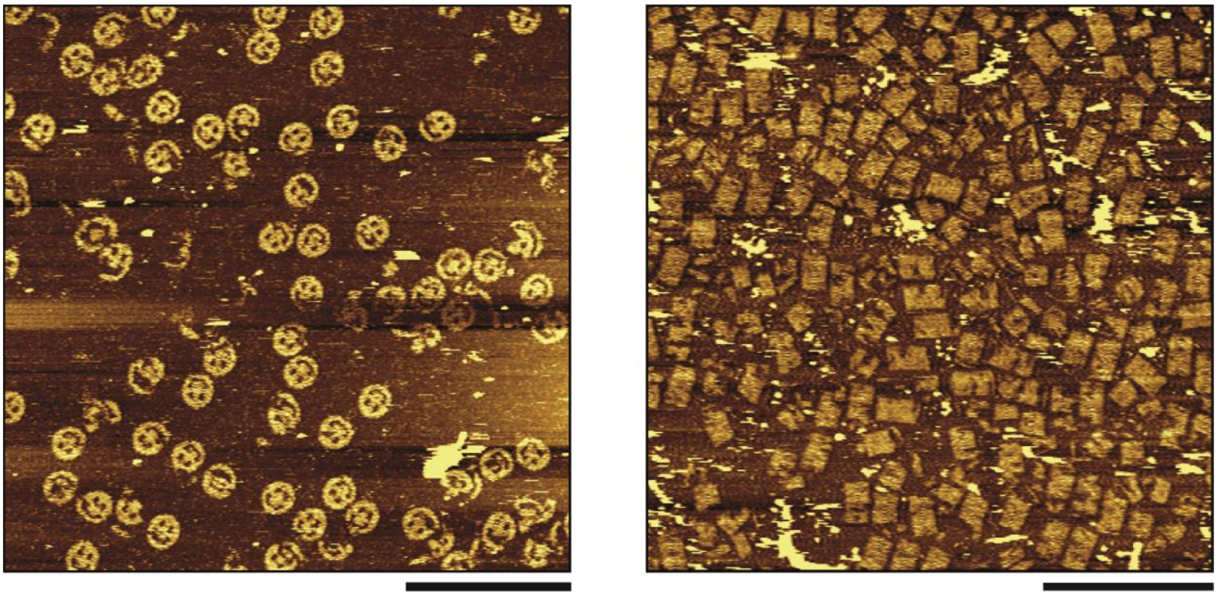

Supplementary Fig. 9. AFM images of the origamis obtained by the isothermal self-assembly of M13 scaffold and staples coding for smileys (left) and rectangles (right) after 24 h incubation at 25 °C in TANa ([NaCl] = 100 mM). [M13] = 1 nM; each staple concentration is 40 nM; no staple purification before AFM imaging. All scale bars are 500 nm.

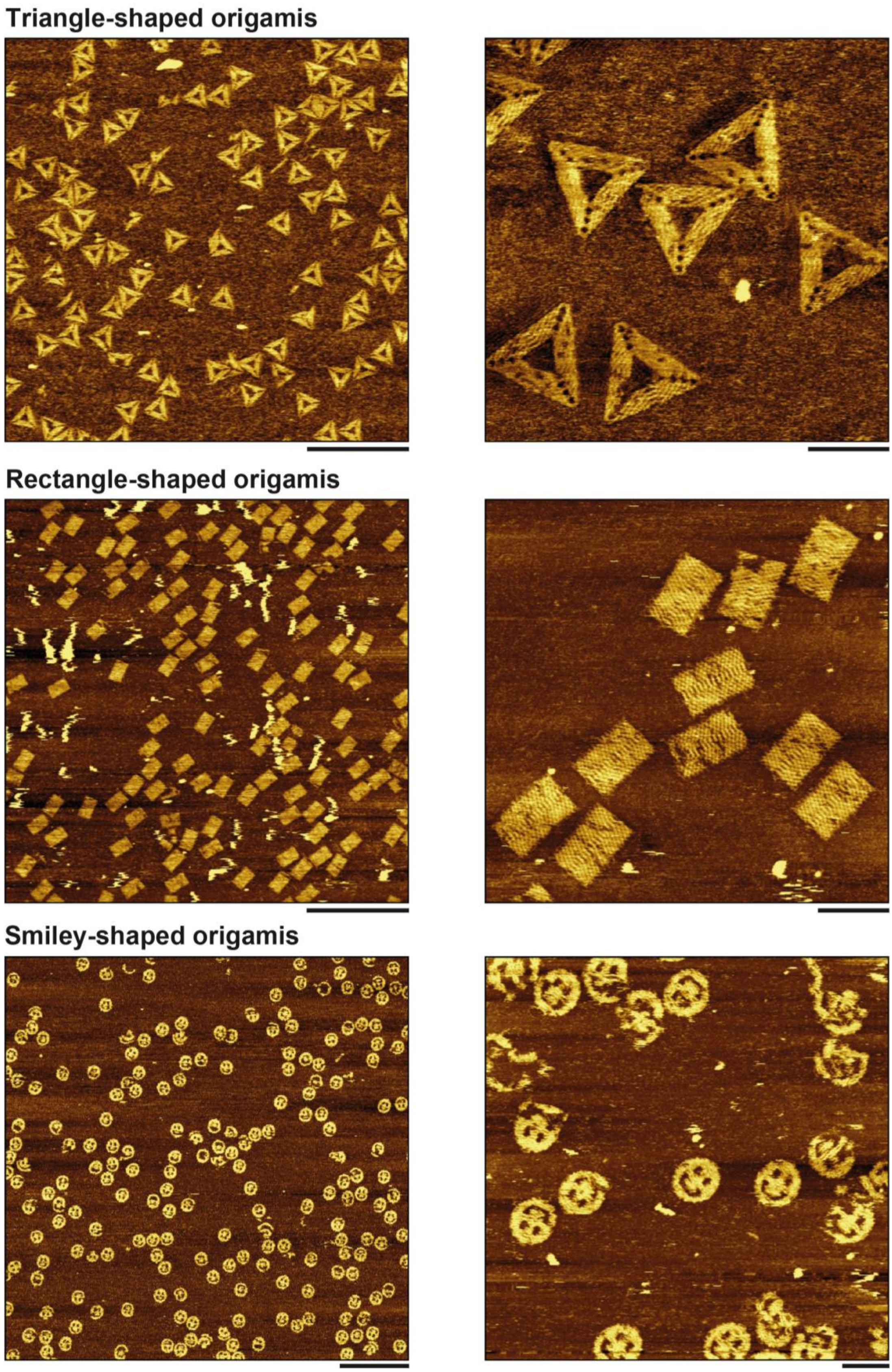

Supplementary Fig. 10. AFM images of triangle (top), rectangle (middle) and smiley (bottom) origamis obtained by thermal annealing in TANa ([NaCl] = 100 mM) with a 40× excess of staples. Scale bars are 500 nm (left column) and 100 nm (right column). [M13] = 1 nM; each staple concentration is 40 nM; no staple purification before AFM imaging.

Supplementary Text 2. Yield of isothermal self-assembly: comparison with thermal annealing and role of staple excess

To quantify the isothermal self-assembly yield, we systematically established the fraction of fully folded (defined as triangles with three well-formed corners), partially folded (defined as incomplete triangular shapes with at least one well-formed corner) and mis-/unfolded (no triangular shape) origamis among a number (*n*) of structures imaged by AFM. After 24 h, the majority of origamis (55% of the detected structures, $n = 209$) were perfectly folded, a high yet lower fraction than with thermal annealing in the same buffer (89%, $n = 167$, Supplementary Fig. 10, *top*). Using a staple excess of 100× led to similar results (Supplementary Fig. 11). Interestingly, partially and fully folded origamis could also be obtained by isothermal assembly at a much lower staple excess (10× down to 2×), but with a fraction of fully folded origamis decreasing with a decrease in staple excess, even with extended incubation times (Supplementary Fig. 12). Ultimately, no proper assembly was obtained with a M13:staples equimolar ratio (Supplementary Fig. 11).

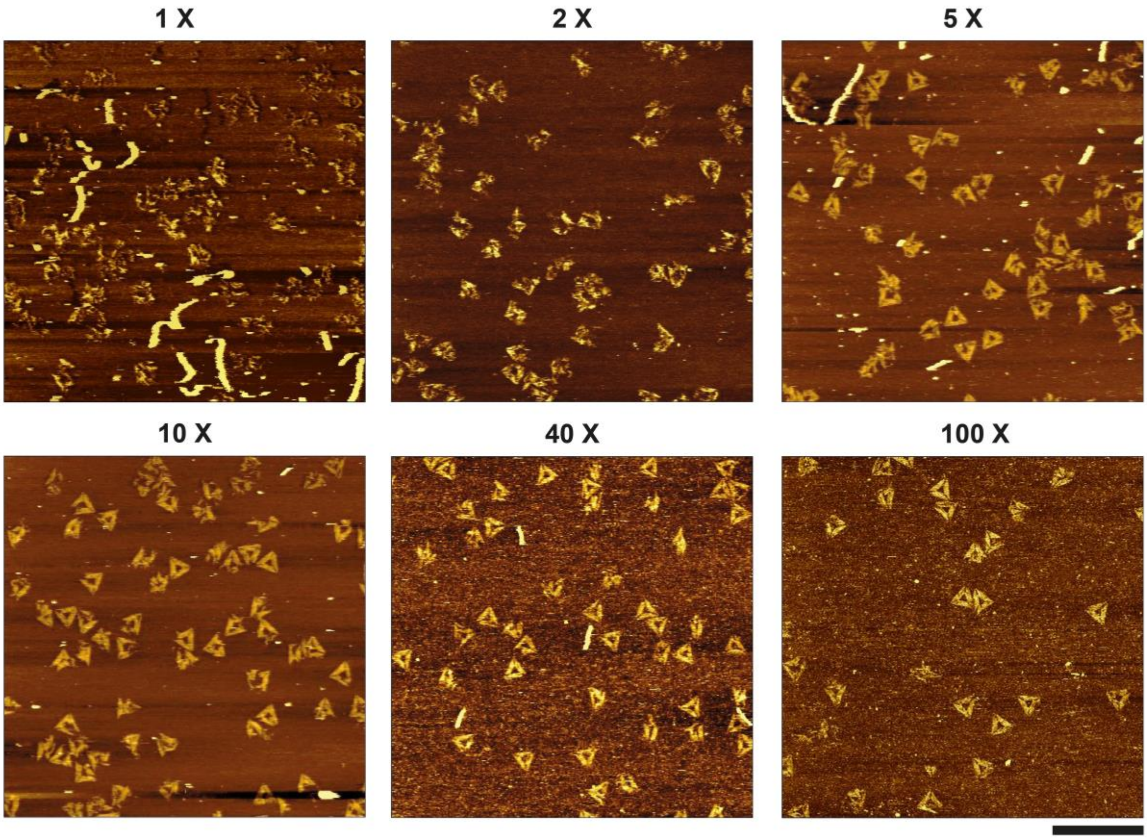

Supplementary Fig. 11. AFM images of structures obtained by isothermal self-assembly at 25 °C of an origami mix coding for sharp triangles in TANa buffer ([NaCl] = 100 mM) after 24 h incubation and for different staple excesses. [M13] = 1 nM; no staple purification before AFM imaging. Scale bar is 500 nm.

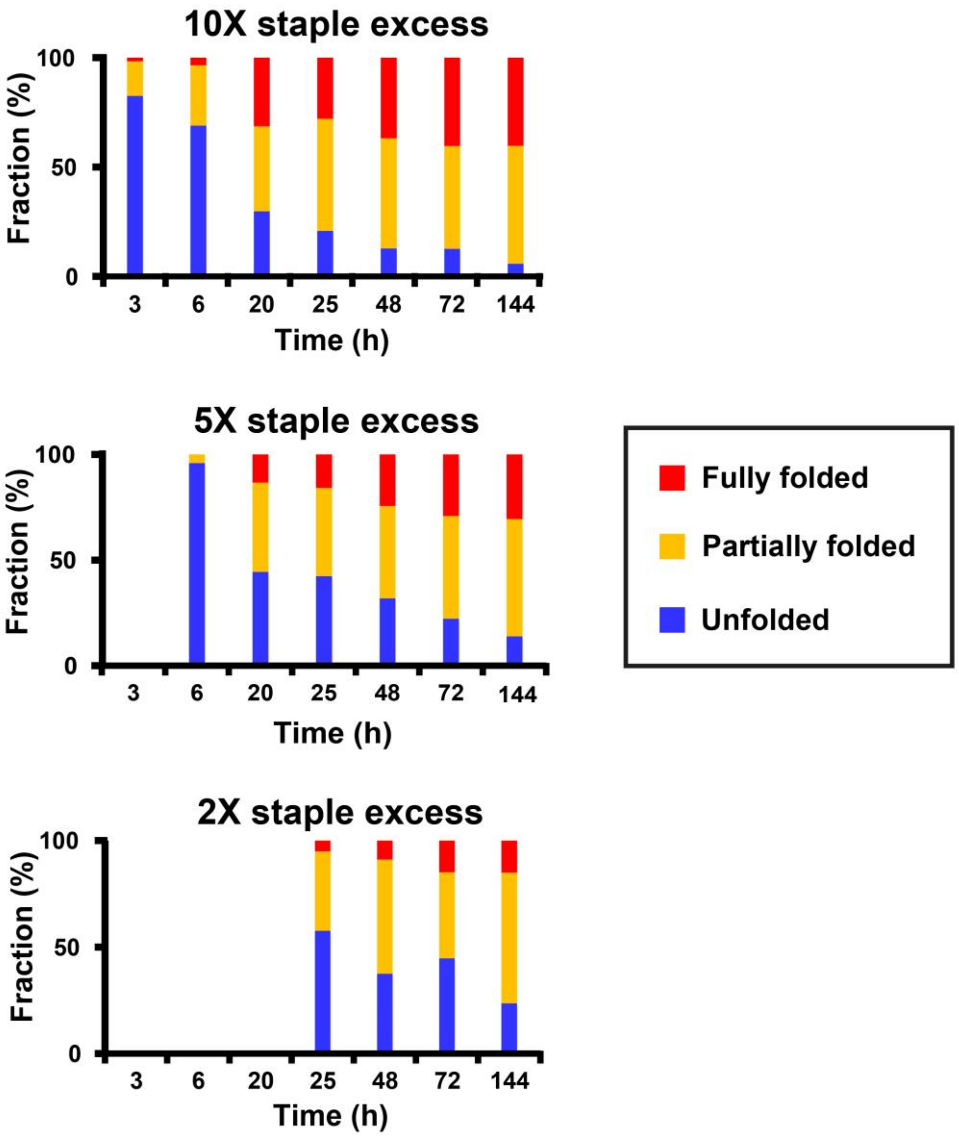

Supplementary Fig. 12. Fraction of unfolded (blue), partially (yellow) and fully (red) folded origamis obtained by isothermal self-assembly at 25 °C of an origami mix coding for sharp triangles in TANa buffer ([NaCl] = 100 mM) for different incubation time (horizontal axes) and for various staple excesses. [M13] = 1 nM; no staple purification before the AFM imaging used to establish these data. The number *n* of analyzed objects for each condition is provided in Supplementary Table 2.

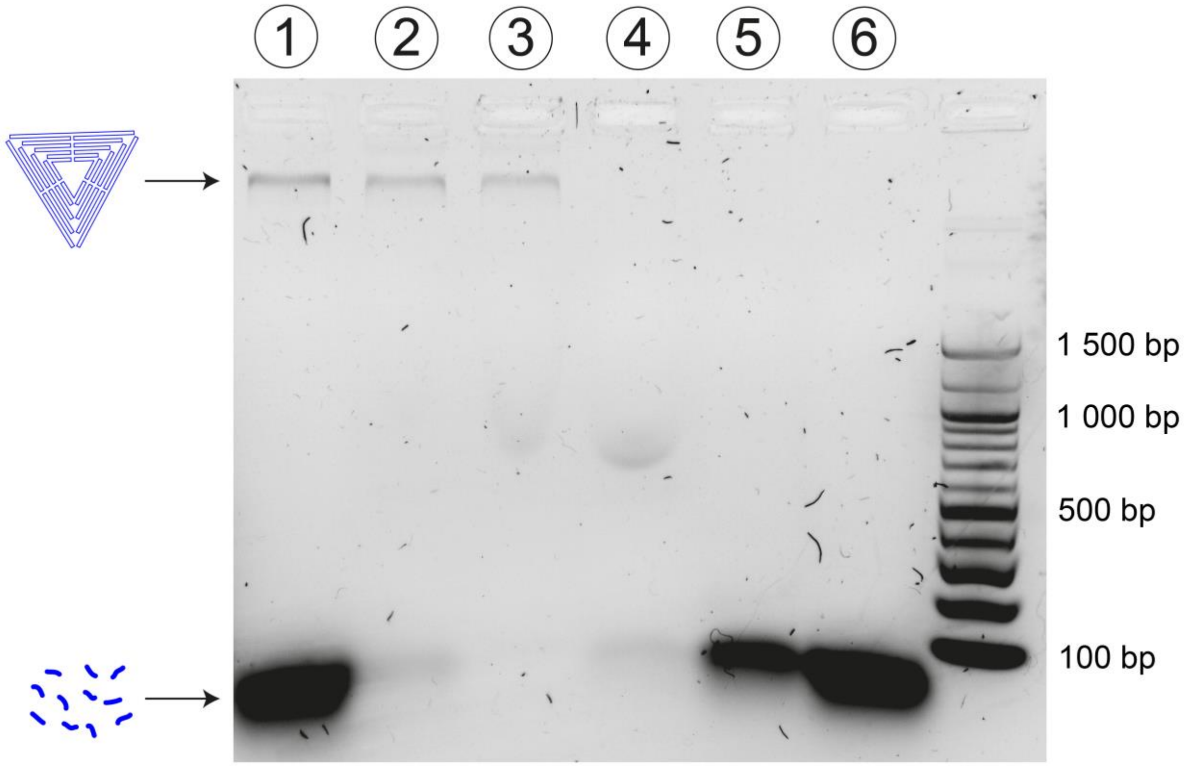

Supplementary Fig. 13. Electrophoresis gel of self-assembly mixtures coding for the sharp-triangle (scheme on the top left), with or without purification by PEG precipitation, and the corresponding staples without the scaffold (schemes on the bottom left). Lane 1: Sample obtained after isothermal self-assembly at 25 °C for 72 h in TANa buffer ([NaCl] = 100 mM), with [M13] = 1 nM and a staple excess of 40×. Lanes 2–3: Same sample as lane 1 but after one (lane 2) or two (lane 3) successive purifications by PEG precipitation. Lanes 4–6: staples only at concentrations corresponding to excess of 1× (lane 4), 10× (lane 5), or 40× (lane 6). Last lane: 100 bp ladder.

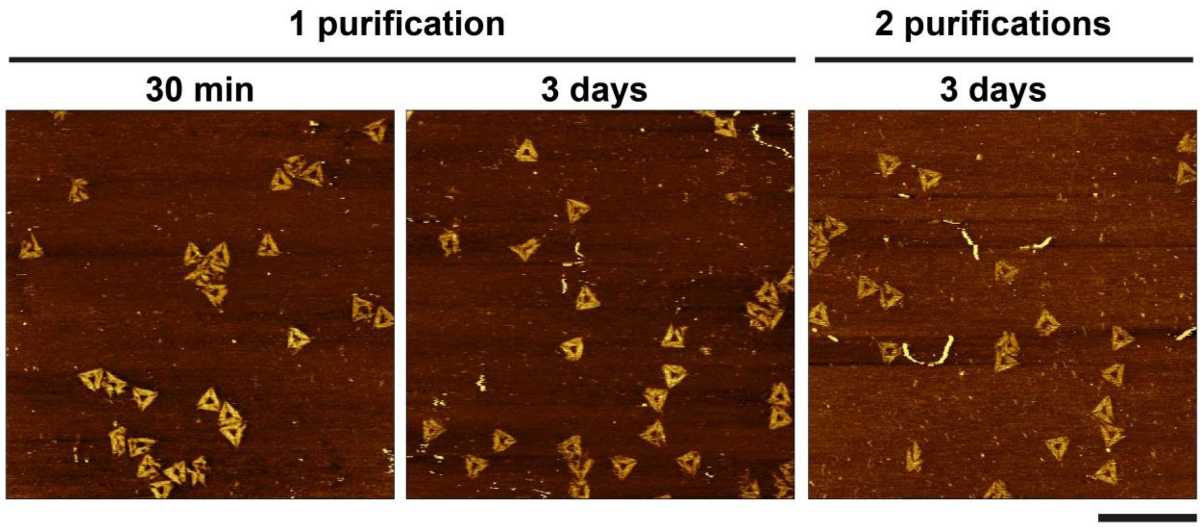

Supplementary Fig. 14. AFM images of sharp triangles ([M13] = 1 nM; 40× excess of staples) obtained after isothermal self-assembly in TANa ([NaCl] = 100 mM) at 25°C for 72 h followed by one (*left*, *middle*) or two (*right*) purifications by PEG precipitation to remove the excess staples, redispersion in TANa ([NaCl] = 100 mM) and incubation at 25 °C for 30 min (*left*) or 3 days (*middle* and *right*). The scale bar is 500 nm.

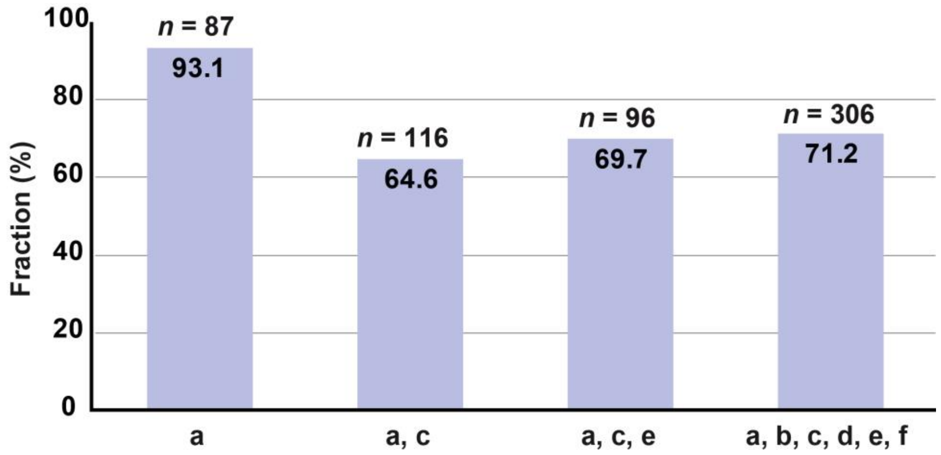

Supplementary Fig. 15. Fraction of available biotinylated sites actually occupied by a bound streptavidin for different compositions in biotinylated staples (namely a to f) after isothermal self-assembly in the conditions of Fig. 2A. *n* indicates the number of analyzed sites.

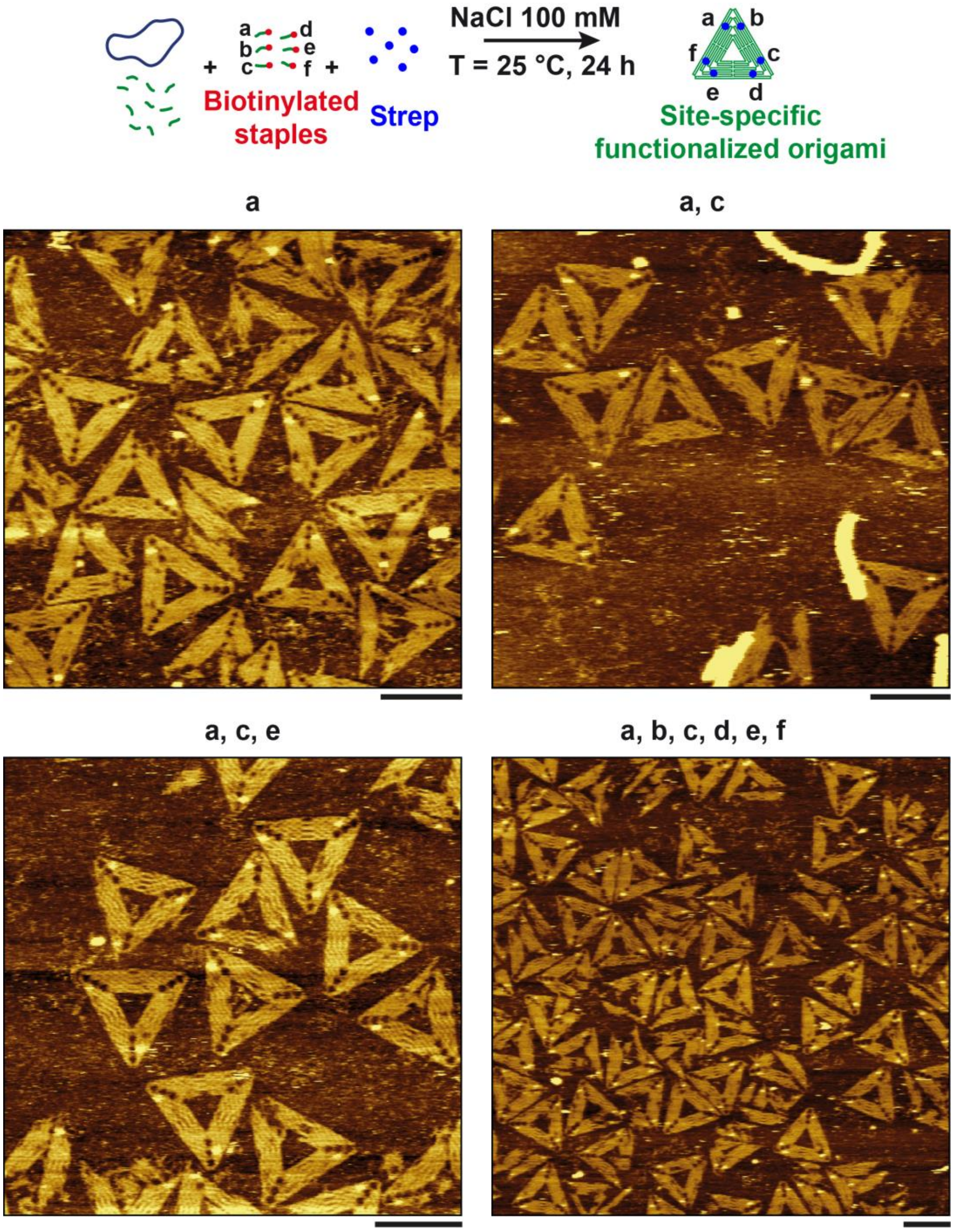

Supplementary Fig. 16. AFM images of site-specific streptavidin-functionalized triangle origamis obtained by the one-step isothermal assembly of M13 scaffold, staples coding for sharp triangles, including biotinylated staples at specific position (named a to e), and 2 µM streptavidin (Strep), after 24 h incubation at 25 °C in TANa ([NaCl] = 100 mM), as a function of the biotinylated staples used to direct the streptavidin functionalization. Same conditions as in Fig. 2A but with larger fields of view. All scale bars are 100 nm.

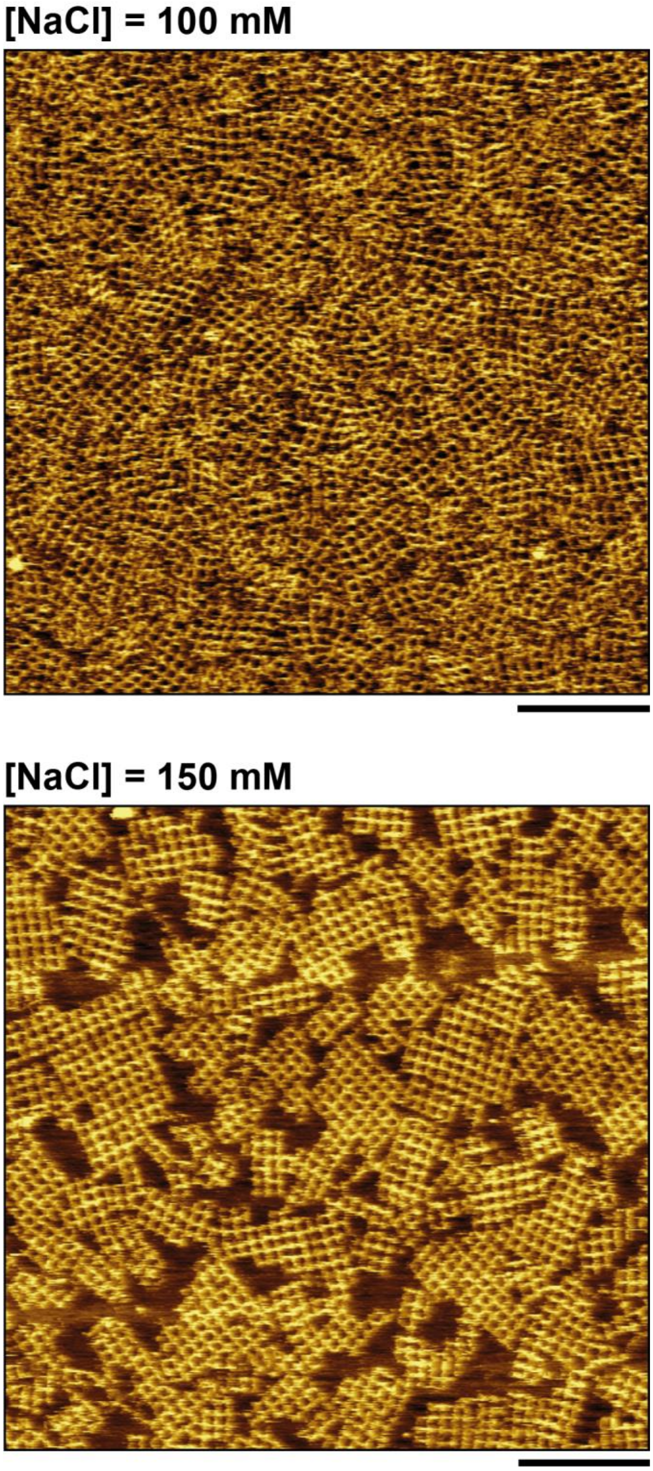

Supplementary Fig. 17. AFM images of nanogrids obtained by the isothermal self-assembly of 9 oligonucleotides at T = 25 °C for 24 h, in TANa buffer with [NaCl] = 100 mM (top) or 150 mM (bottom). Same conditions as in Fig. 2C but with larger fields of view. Scale bars are 200 nm.

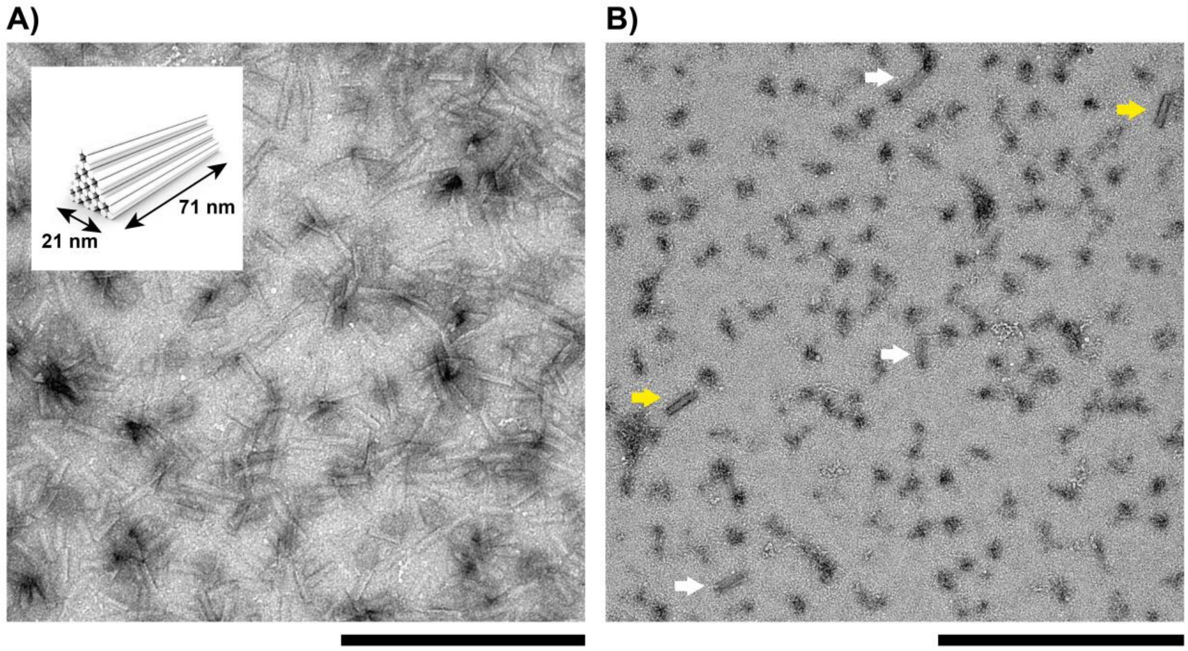

Supplementary Fig. 18. Negative-stain transmission electron microscopy (TEM) images of the structures obtained by thermal annealing (A) or isothermal assembly (B) of Tb “Toblerone”-like structures (scheme in inset) after removal of the excess staples by gel electrophoresis. A) Structures obtained after 41 h of thermal annealing in an optimized Mg buffer (Tris-HCl 5 mM, pH = 8.0, EDTA 1 mM, $MgCl_2$ 18 mM). B) Structures obtained by isothermal self-assembly (no thermal pretreatment) in TANa buffer with [NaCl] = 100 mM at 25 °C for 48 h. Arrows indicate well-formed Tb (yellow) and partially folded structures with similarities in shape and sizes with the target (white). All scale bars are 500 nm.

Supplementary Text 3. Real time AFM imaging of origami folding pathway

To keep most of the dynamic features possible upon surface adsorption for AFM imaging, we used a mica substrate coated by a supported lipid bilayer. It was shown in previous works that origamis could adsorb on such substrates through electrostatic interactions mediated by magnesium ions and keep their mobility once adsorbed.[5] To avoid the presence of magnesium ions, we chose another adsorption method by using a few cholesterol-modified staples to anchor parts of the origamis to the bilayer-coated surface. We prepared Λ-shaped origamis by thermal annealing in the TANa buffer by using an origami mix for a triangular shape ([M13] = 1 nM, 40× staple excess) with cholesterol modifications on two of the sides but depleted from the staples of the third side (Supplementary Fig. 19), and absorbed them on the lipid bilayer. Each adsorbed object was therefore composed of two anchored folded sides and an unfolded, unmodified single-stranded M13 fragment with no prescribed folding constrain and minimal interactions with the surface. This system was then maintained at 25 °C in the TANa buffer containing unadsorbed Λ structures and their excess of staples. We added to this solution the staples of the sharp triangle missing side (same concentration as for the other sides to keep a 40× staple excess) and followed *in situ* and in real time the folding of the M13 fragment (Supplementary Movies 1-4). Contrary to experiments performed so far allowing to make snapshots at a given time of a population of origamis,[6] this method provided the whole temporal evolution of individual structures from an unfolded M13 fragment to the folded state at equilibrium.

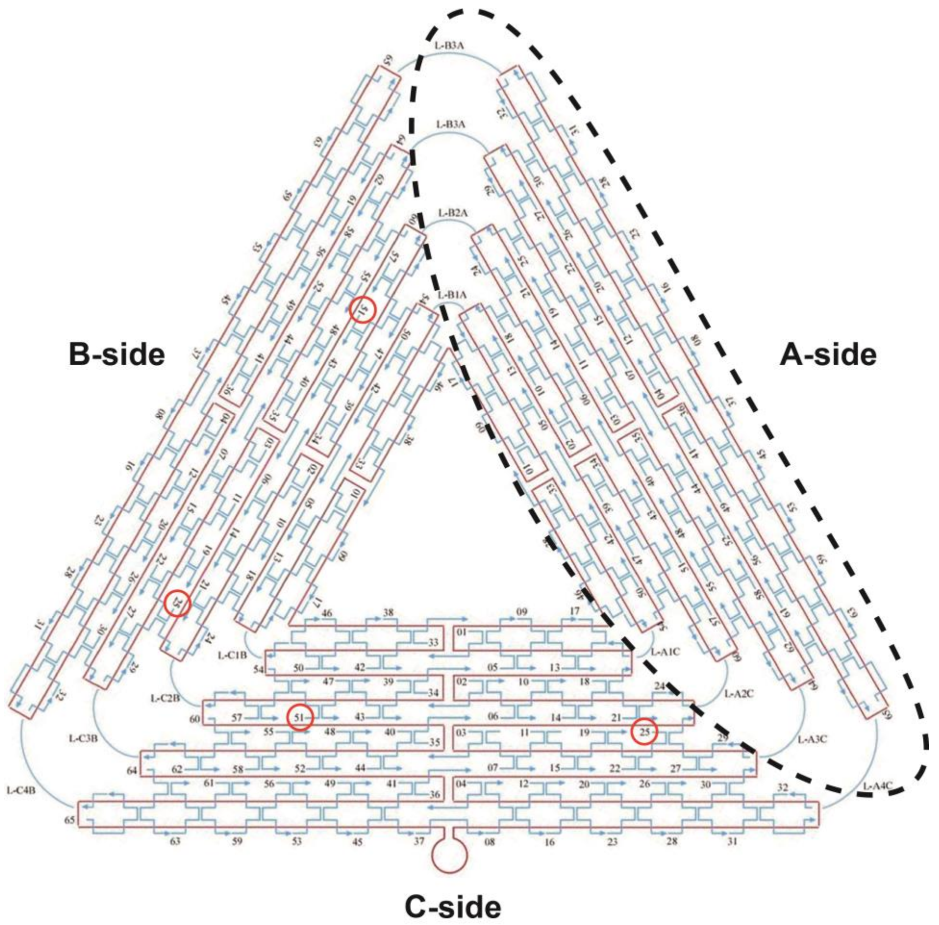

Supplementary Fig. 19. Map of the sharp-triangle origami with cholesterol-modified staples. The dotted black circle surrounds the side of the triangle which was removed to form the Λ origamis. The red circles point out the position of the cholesterol-modified staples integrated to the Λ origamis.

Supplementary Text 4. Optimal shape selection in a competitive staple mixture

The shape competition experiments was performed by mixing M13 with two equimolar sets of staples coding for sharp triangles and tall rectangles without staple edges. Properly folding rectangle would lead to 1105 unpaired bases for 64 free ends, *i.e.*, double stranded helices that have no neighboring base pair and thus no stacking partner, while the sharp triangle structures involved only 97 unpaired bases and 54 free ends. We could thus expect the sharp triangle to be on a lower energetic level and therefore thermodynamically more stable since more bases of the scaffold are paired in the fully assembled state. We let the system evolve at 25 °C in TANa buffer ([NaCl] = 100 mM). Regardless of the incubation time, from a few minutes to several days, we did not detect any rectangular shapes, neither partial or complete, nor any triangle-rectangle chimera (Supplementary Fig. 20). During the first hour of the competition, although a small number of staples were probably properly binding to the M13 scaffold, the structures were not folded enough to distinguish any preferential shape. In contrast, after 4 h of incubation, we observed the progressive self-assembly of sharp triangle origamis only, showing that the system spontaneously evolved toward the lower free energy minimum. We expect that chimera could appear but only when they correspond to a free energy minimum.[7]

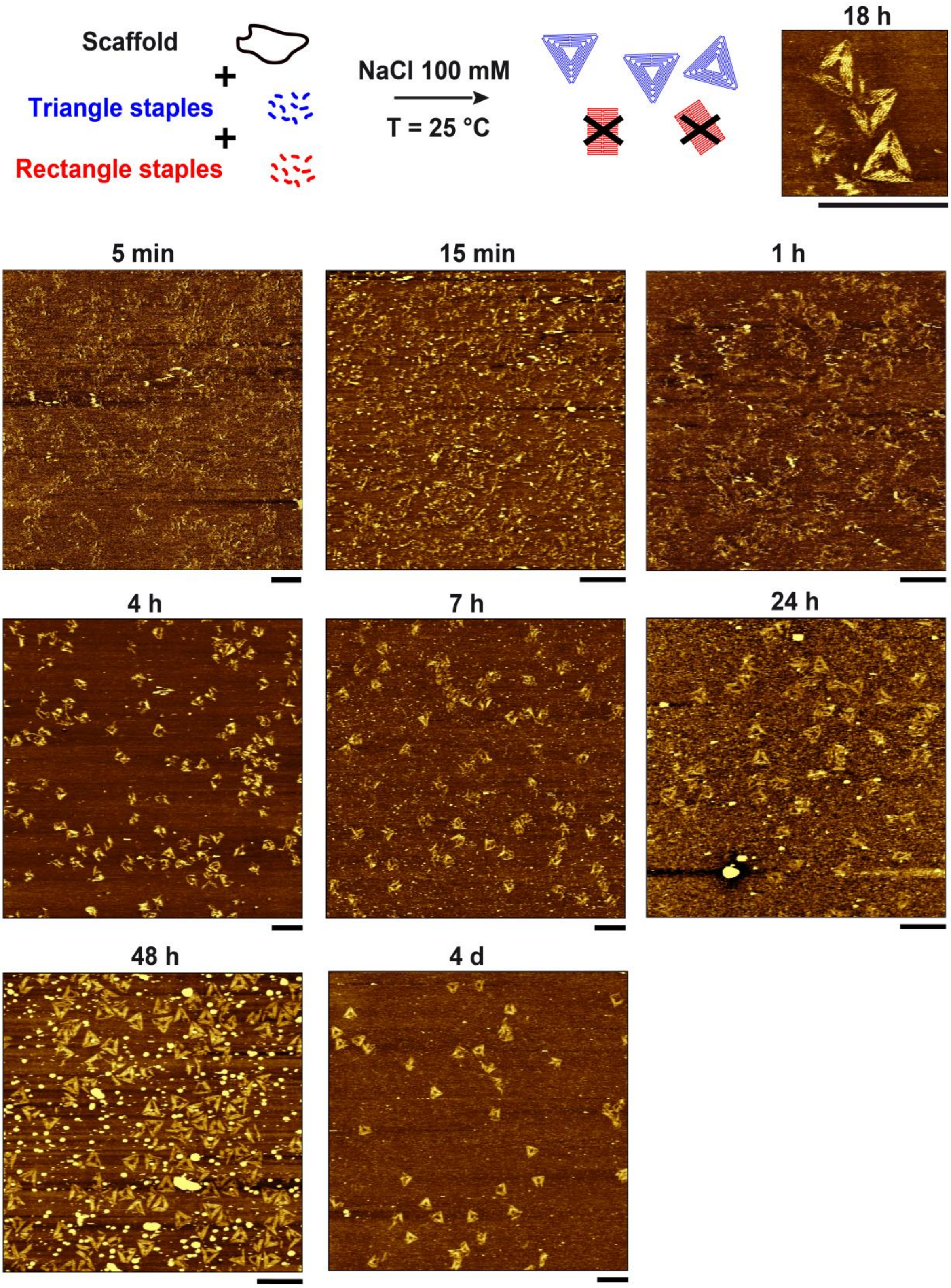

Supplementary Fig. 20. AFM images of the evolution of the structures obtained along incubation time by the isothermal self-assembly of M13 scaffold and a mixture of two staple sets coding for triangles and rectangles at 25 °C in TANa buffer ([NaCl] = 100 mM). Triangles progressively self-assemble while no partially/fully rectangles nor chimeric structures are obtained. [M13] = 1 nM; each staple concentration is 40 nM. All scale bars are 300 nm.

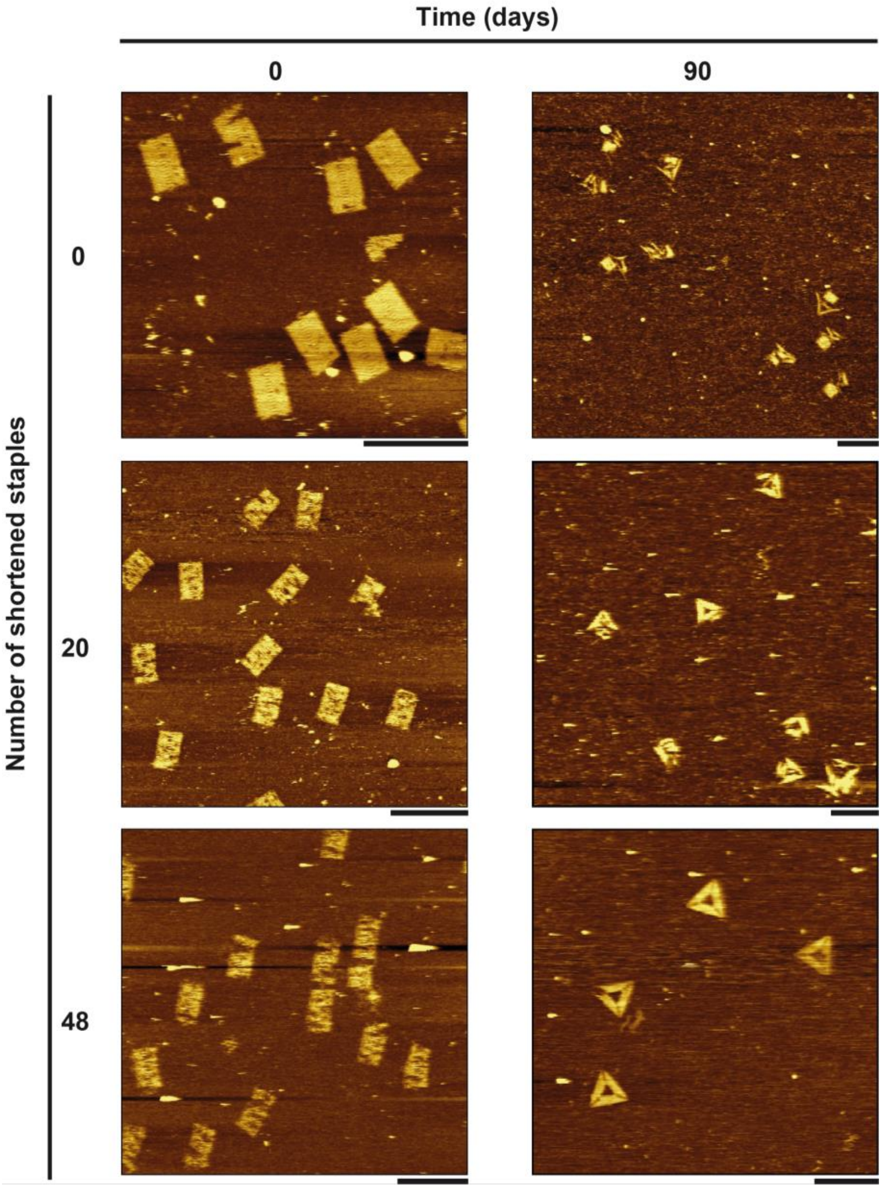

Supplementary Fig. 21. AFM images of a transformation experiment performed in TANa buffer at 30 °C as in Figure 5 but with [NaCl] = 150 mM (instead of 100 mM). All scale bars are 200 nm.

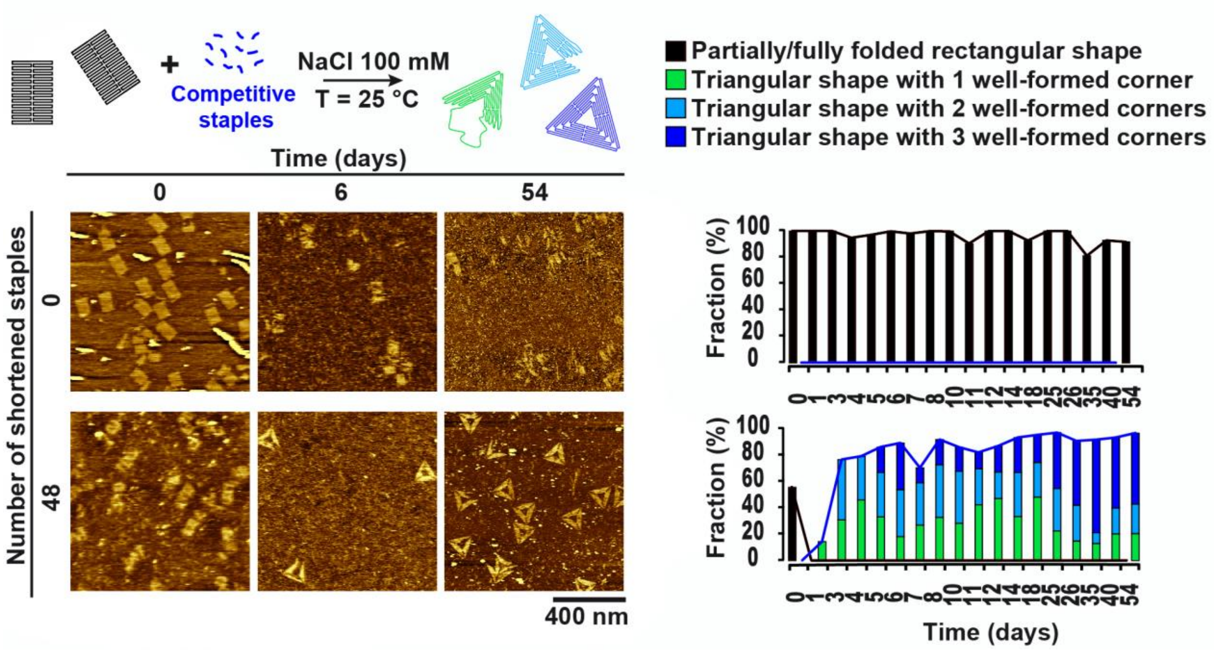

Supplementary Fig. 22. AFM images (left) and fraction of detected objects over time in a transformation experiment perfomed in TANa buffer with [NaCl] = 100 mM as in Figure 5 but at a fixed temperature of 25 °C (instead of 30 °C). The number of analyzed objects for each condition is given in Supplementary Table 4.

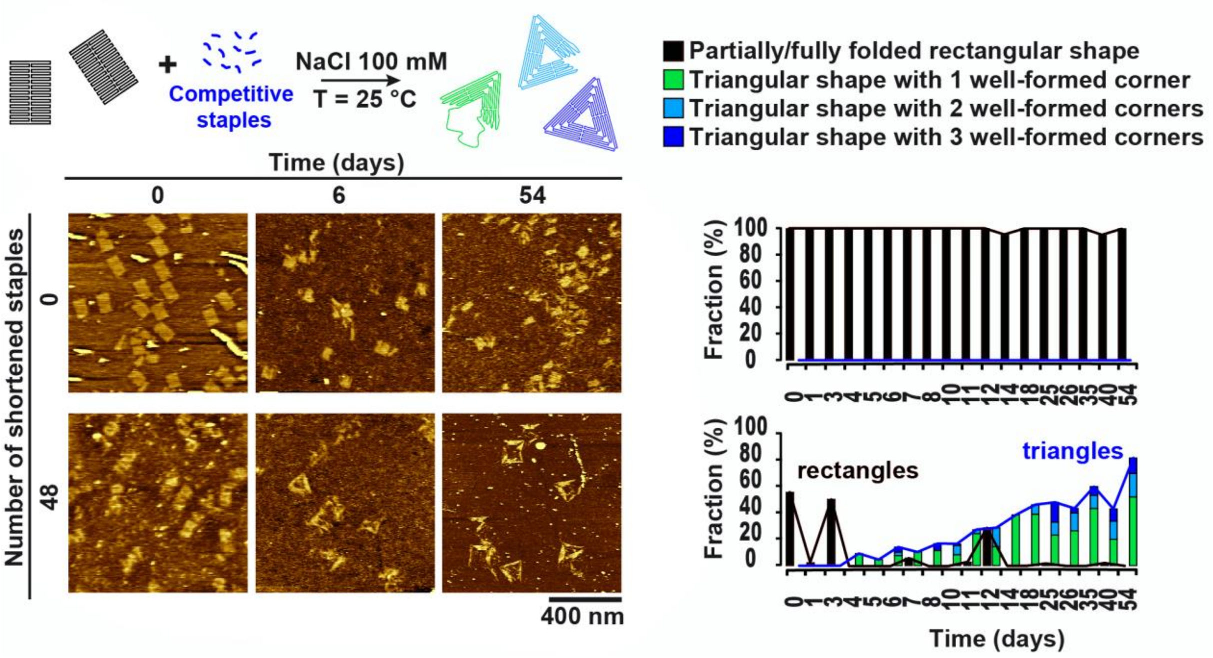

Supplementary Fig. 23. AFM images (left) and fraction of detected objects over time in a transformation experiment performed as in Supplementary Fig. 22 ([NaCl] = 100 mM ; T = 25 °C) but with a rectangle over triangle staple ratio of 1. [M13] = 0.25 nM; each staple concentration is 10 nM for both rectangles and triangles. The number of analyzed objects for each condition is given in Supplementary Table 5.

Supplementary Text 5. Spatially resolved stepwise assembly

We achieved spatial control over the assembly progress by successfully programming the stepwise assembly of origamis by successive addition of different fractions of the staple set in the self-assembling mixture. This allowed us, for instance, to program the construction of sharp triangles either from a corner to the opposite edge (Supplementary Fig. 24A), or from an edge to the opposite corner (Supplementary Fig. 24B).

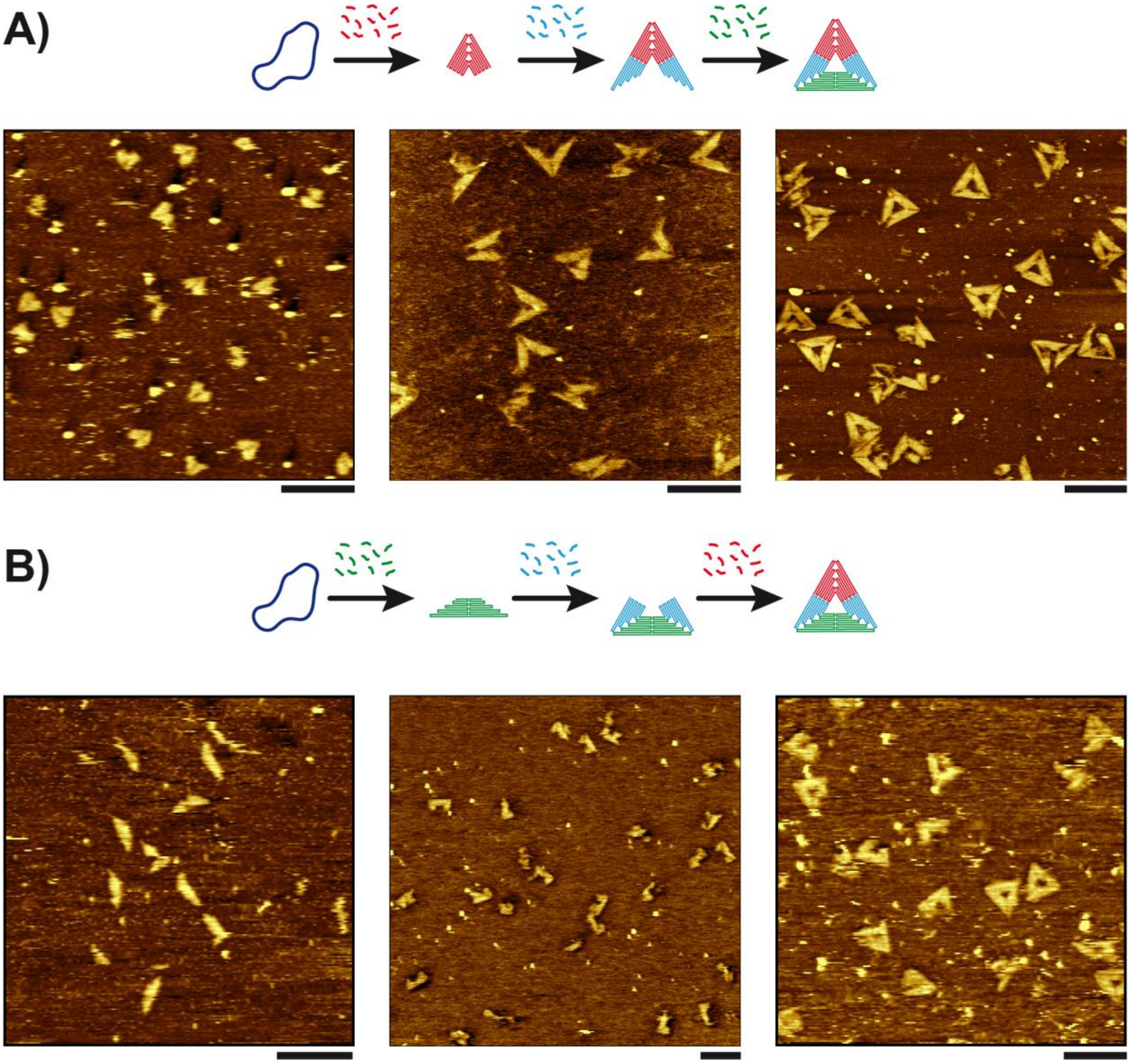

Supplementary Fig. 24. AFM images of the structures obtained by stepwise isothermal assembly of an origami mix coding for sharp triangles in TANa buffer ([NaCl] =100 mM) at 25°C, from one corner to the opposite side (top row) and from one side to the opposite corner (bottom row). At each step, a fraction of staples coding for a specific triangle part is added to the mixture and the image shows the resulting structure after 24 h of incubation. Scale bars are 200 nm.

3) Supplementary Tables

Supplementary Table 1. Number *n* of analyzed objects for each condition displayed in Supplementary Fig. 3.

T°C / [NaCl]	4 °C	15 °C	20 °C	25 °C	30 °C	35 °C	40 °C	45 °C	50 °C	55 °C	60 °C
**50 mM**	*n*=28	*n*=35	*n*=126	*n*=102	*n*=40	*n*=171	*n*=54	*n*=40	*n*=52	*n*=72	*n*=149
**100 mM**	*n*=118	*n*=462	*n*=413	*n*=209	*n*=639	*n*=243	*n*=112	*n*=230	*n*=92	*n*=115	*n*=10
**150 mM**	*n*=179	*n*=229	*n*=251	*n*=241	*n*=295	*n*=297	*n*=263	*n*=160	*n*=7	*n*=137	*n*=190
**250 mM**	*n*=170	*n*=188	*n*=387	*n*=220	*n*=308	*n*=287	*n*=218	*n*=170	*n*=284	*n*=194	*n*=314

Supplementary Table 2. Number *n* of analyzed objects for each condition displayed in Supplementary Fig. 12.

Time (h) / Excess of staples per scaffold	3	6	20	25	48	72	144
**10 X**	*n*=63	*n*=29	*n*=185	*n*=447	*n*=133	*n*=119	*n*=154
**5 X**	--	*n*=122	*n*=378	*n*=378	*n*=119	*n*=247	*n*=216
**2 X**	--	--	--	*n*=236	*n*=112	*n*=201	*n*=233

Supplementary Table 3. Number *n* of analyzed objects for each condition displayed in Figure 5.

Time (days) / Number of shortened staples	0	1	4	6	7	8	11	18	42
**0**	*n*=111	*n*=22	*n*=30	*n*=49	*n*=33	*n*=85	*n*=47	*n*=32	*n*=561
**20**	*n*=134	*n*=22	*n*=47	*n*=45	*n*=38	*n*=30	*n*=45	*n*=18	*n*=192
**48**	*n*=165	*n*=57	*n*=42	*n*=50	*n*=53	*n*=101	*n*=20	*n*=47	*n*=366

Supplementary Table 4. Number *n* of analyzed objects for each condition displayed in Supplementary Fig. 22.

Time (days) / Num of shortened staples	0	1	3	4	5	6	7	8	10	11	12	14	18	25	26	35	40	54
**0**	*n*=111	*n*=34	*n*=34	*n*=37	*n*=38	*n*=43	*n*=59	*n*=62	*n*=66	*n*=32	*n*=64	*n*=23	*n*=43	*n*=84	*n*=42	*n*=37	*n*=87	*n*=48
**48**	*n*=165	*n*=7	*n*=13	*n*=24	*n*=18	*n*=28	*n*=71	*n*=88	*n*=171	*n*=40	*n*=85	*n*=15	*n*=87	*n*=205	*n*=109	*n*=38	*n*=45	*n*=204

Supplementary Table 5. Number *n* of analyzed objects for each condition displayed in Supplementary Fig. 23.

Num of shortened staples \ Time (days)	0	1	3	4	5	6	7	8	10	11	12	14	18	25	26	35	40	54
**0**	*n*= 111	*n*= 57	*n*= 41	*n*= 73	*n*= 66	*n*= 76	*n*= 50	*n*= 77	*n*= 77	*n*= 57	*n*= 66	*n*= 21	*n*= 60	*n*= 123	*n*= 78	*n*= 52	*n*= 81	*n*= 90
**48**	*n*= 165	*n*= 49	*n*= 4	*n*= 22	*n*= 70	*n*= 80	*n*= 19	*n*= 61	*n*= 98	*n*= 37	*n*= 7	*n*= 13	*n*= 54	*n*= 73	*n*= 53	*n*= 30	*n*= 65	*n*= 60

4) Legends of the Supplementary Movies

Supplementary Movie 1. Direct observation of the Λ-to Δ-origami transition on a mica-supported lipid bilayer (DOPC) in the TAENa buffer at room-temperature (T = 26 °C). At t = 0 min, addition of the missing A-side staples (Supplementary Fig. 19), following the protocol given in Methods section, paragraph "Real-time imaging of the Λ→Δ isothermal evolution on a lipid bilayer surface". Observation by AFM at the same position over 223 min. Scale bar: 300 nm.

Supplementary Movie 2. Crop (375 nm x 375 nm) of Supplementary Movie 1 around the origami labelled B in Fig. 4.

Supplementary Movie 3. Crop (375 nm x 375 nm) of Supplementary Movie 1 around the origami labelled C in Fig. 4.

Supplementary Movie 4. Crop (375 nm x 375 nm) of Supplementary Movie 1 around the origami labelled D in Fig. 4.

5) Supplementary References

1. Rothemund, P. W. K. Folding DNA to create nanoscale shapes and patterns. *Nature* **440**, 297–302 (2006).

2. Wei, B., Dai, M. & Yin, P. Complex shapes self-assembled from single-stranded DNA tiles. *Nature* **485**, 623–626 (2012).

3. Yan, H., Park, S. H., Finkelstein, G., Reif, J. H. & LaBean, T. H. DNA-Templated Self-Assembly of Protein Arrays and Highly Conductive Nanowires. *Science (1979)* **301**, 1882–1884 (2003).

4. Sigl, C. *et al.* Programmable icosahedral shell system for virus trapping. *Nat Mater* **20**, 1281–1289 (2021).

5. Suzuki, Y., Endo, M. & Sugiyama, H. Lipid-bilayer-assisted two-dimensional self-assembly of DNA origami nanostructures. *Nat Commun* **6**, 8052 (2015).

6. Lee Tin Wah, J., David, C., Rudiuk, S., Baigl, D. & Estevez-Torres, A. Observing and Controlling the Folding Pathway of DNA Origami at the Nanoscale. *ACS Nano* **10**, 1978–1987 (2016).

7. Majikes, J. M., Nash, J. A. & LaBean, T. H. Competitive annealing of multiple DNA origami: formation of chimeric origami. *New J Phys* **18**, 115001 (2016).

6. Supplementary Source Data

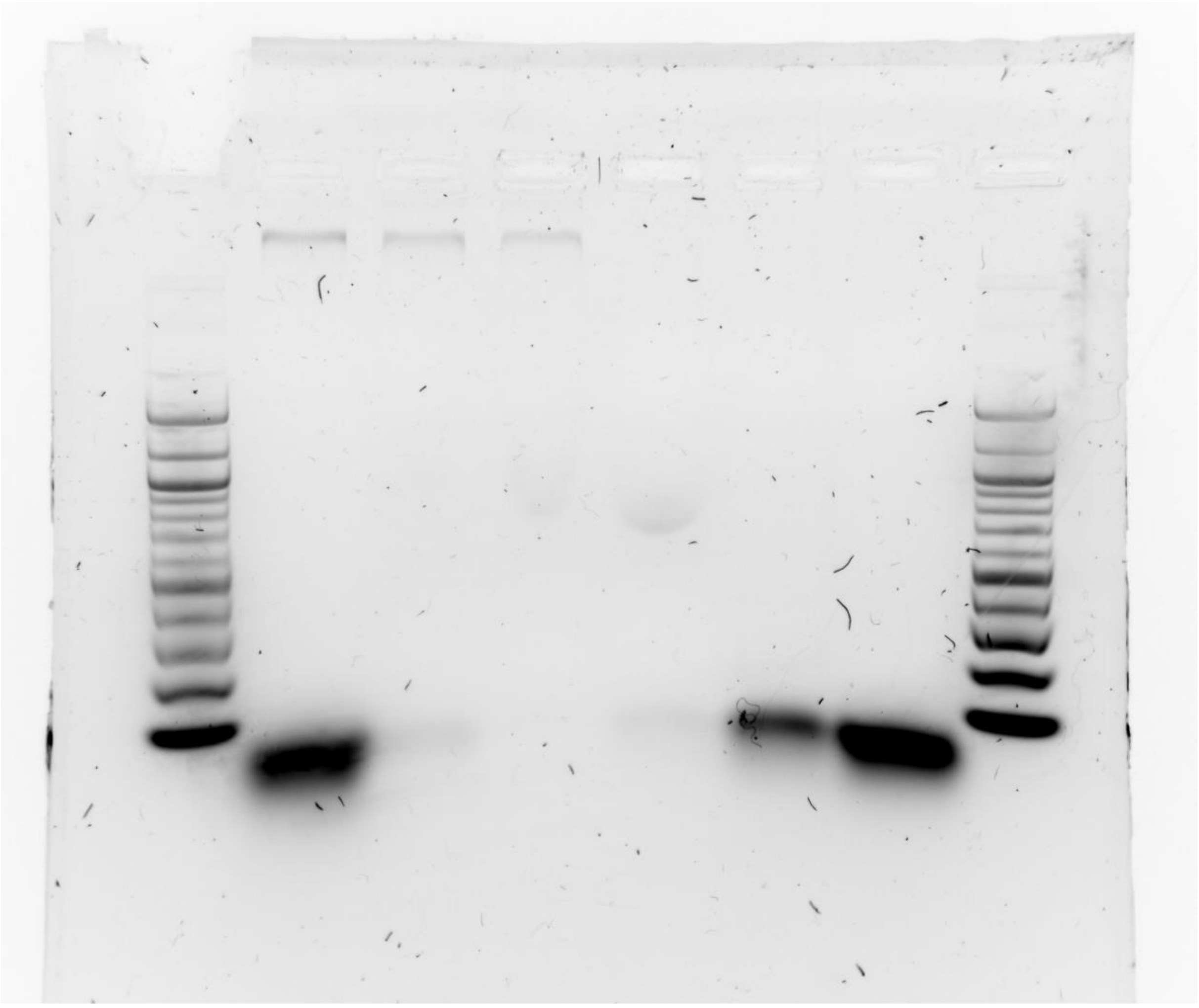

Uncropped gel displayed in Supplementary Fig. 13
