# Supplementary figures and images for: Isothermal self-assembly of multicomponent and evolutive DNA nanostructures
Source: Nat Nanotechnol. 2023 Jul 31;18(11):1311–8. doi: 10.1038/s41565-023-01468-2 (PMC10656289; doi:10.1038/s41565-023-01468-2)

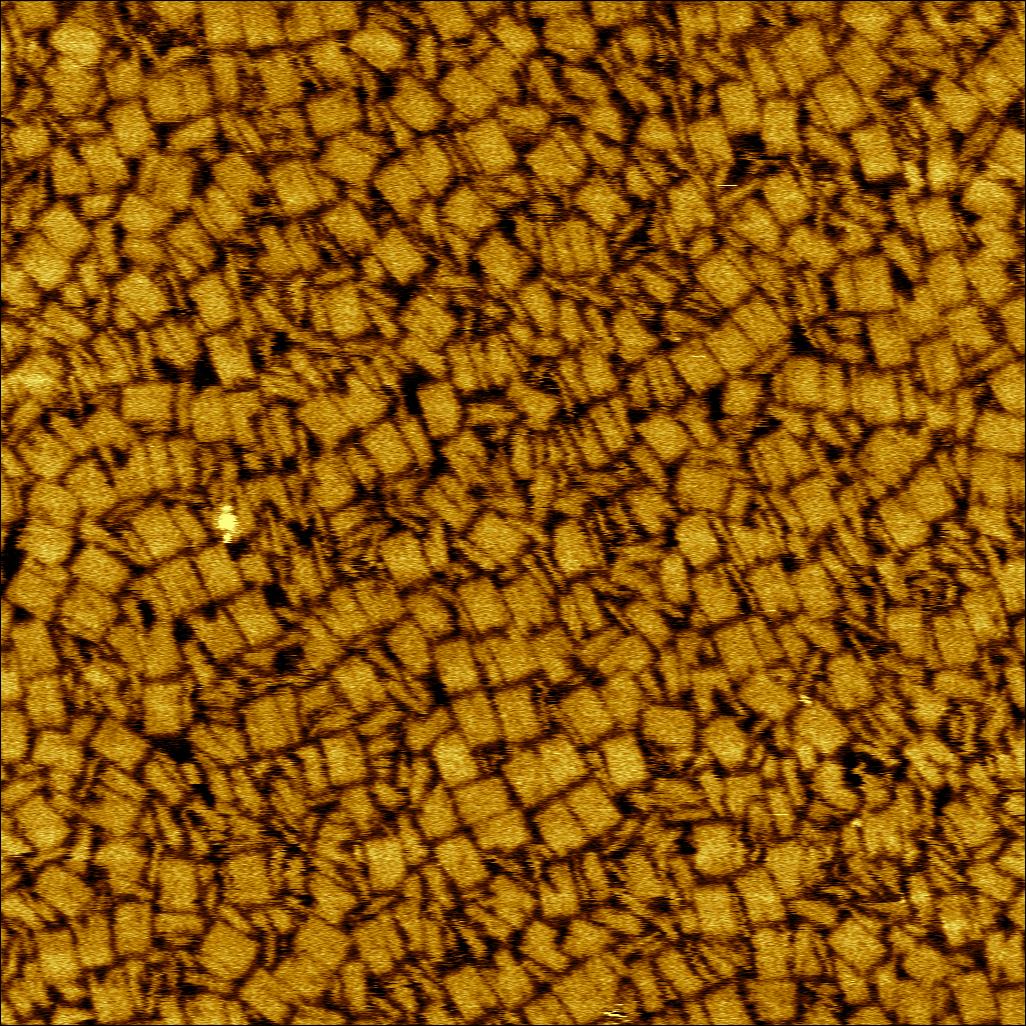

Supplement: Supplementary file 7 — Source data of Figure 2A, Uncropped AFM image of Figure 2B, Uncropped AFM image of Figure 2B, Uncropped AFM image of Figure 2B, Uncropped AFM image of Figure 2B, Uncropped AFM image of Figure 2B, Uncropped Gel image of Figure 2B [file 41565_2023_1468_MOESM7_ESM.zip › Source Data Figure 2/Figure 2B Uncropped AFM 168h.jpg]

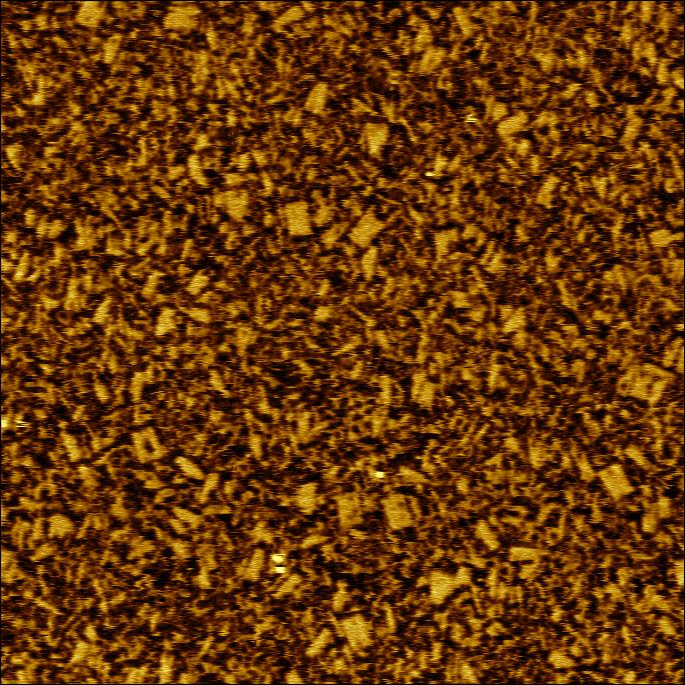

Supplement: Supplementary file 7 — Source data of Figure 2A, Uncropped AFM image of Figure 2B, Uncropped AFM image of Figure 2B, Uncropped AFM image of Figure 2B, Uncropped AFM image of Figure 2B, Uncropped AFM image of Figure 2B, Uncropped Gel image of Figure 2B [file 41565_2023_1468_MOESM7_ESM.zip › Source Data Figure 2/Figure 2B Uncropped AFM 16h.jpg]

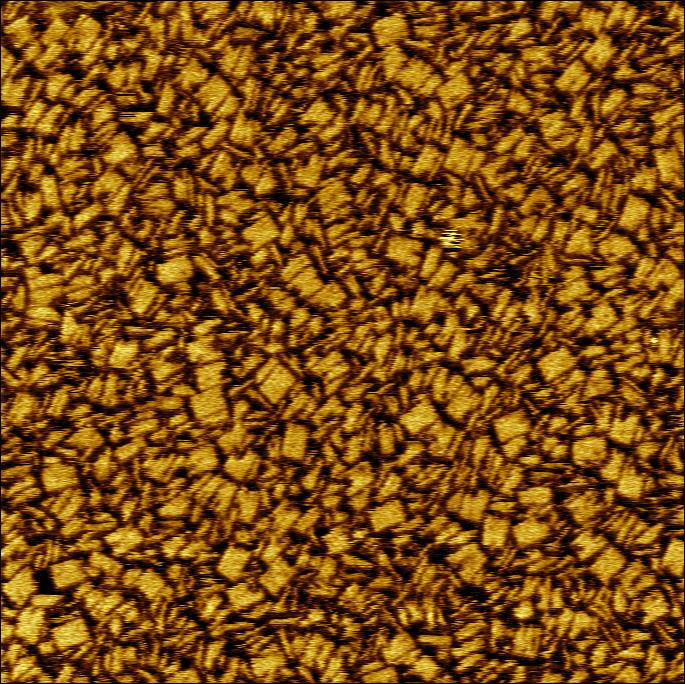

Supplement: Supplementary file 7 — Source data of Figure 2A, Uncropped AFM image of Figure 2B, Uncropped AFM image of Figure 2B, Uncropped AFM image of Figure 2B, Uncropped AFM image of Figure 2B, Uncropped AFM image of Figure 2B, Uncropped Gel image of Figure 2B [file 41565_2023_1468_MOESM7_ESM.zip › Source Data Figure 2/Figure 2B Uncropped AFM 25h.jpg]

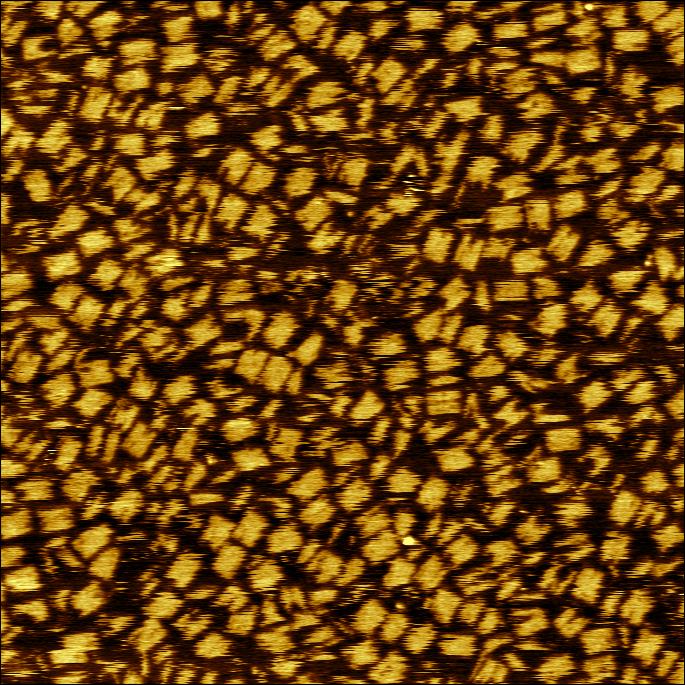

Supplement: Supplementary file 7 — Source data of Figure 2A, Uncropped AFM image of Figure 2B, Uncropped AFM image of Figure 2B, Uncropped AFM image of Figure 2B, Uncropped AFM image of Figure 2B, Uncropped AFM image of Figure 2B, Uncropped Gel image of Figure 2B [file 41565_2023_1468_MOESM7_ESM.zip › Source Data Figure 2/Figure 2B Uncropped AFM 72h.jpg]

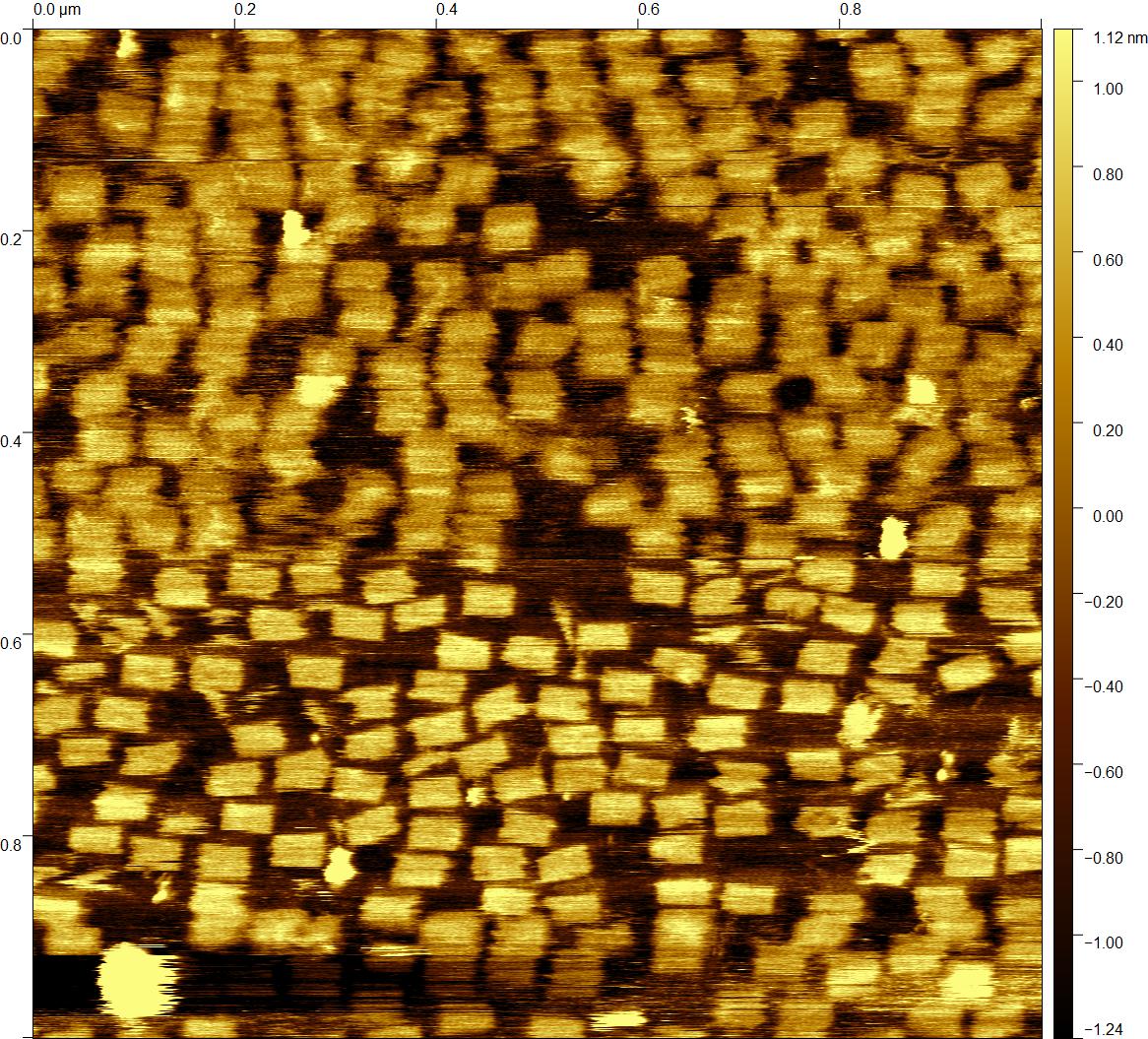

Supplement: Supplementary file 7 — Source data of Figure 2A, Uncropped AFM image of Figure 2B, Uncropped AFM image of Figure 2B, Uncropped AFM image of Figure 2B, Uncropped AFM image of Figure 2B, Uncropped AFM image of Figure 2B, Uncropped Gel image of Figure 2B [file 41565_2023_1468_MOESM7_ESM.zip › Source Data Figure 2/Figure 2B Uncropped AFM purified tiles.jpg]

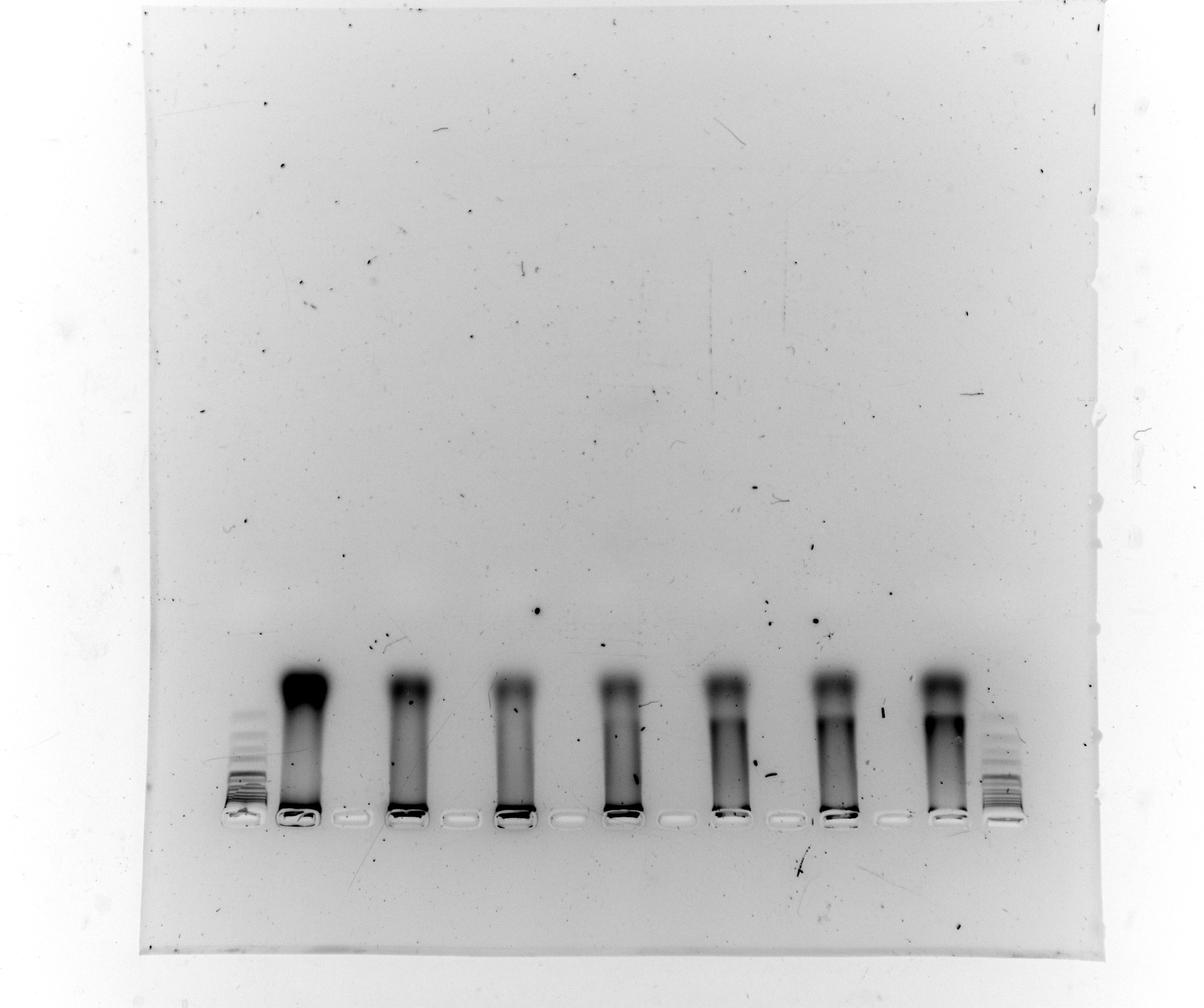

Supplement: Supplementary file 7 — Source data of Figure 2A, Uncropped AFM image of Figure 2B, Uncropped AFM image of Figure 2B, Uncropped AFM image of Figure 2B, Uncropped AFM image of Figure 2B, Uncropped AFM image of Figure 2B, Uncropped Gel image of Figure 2B [file 41565_2023_1468_MOESM7_ESM.zip › Source Data Figure 2/Figure 2B Uncropped Gel.jpg]

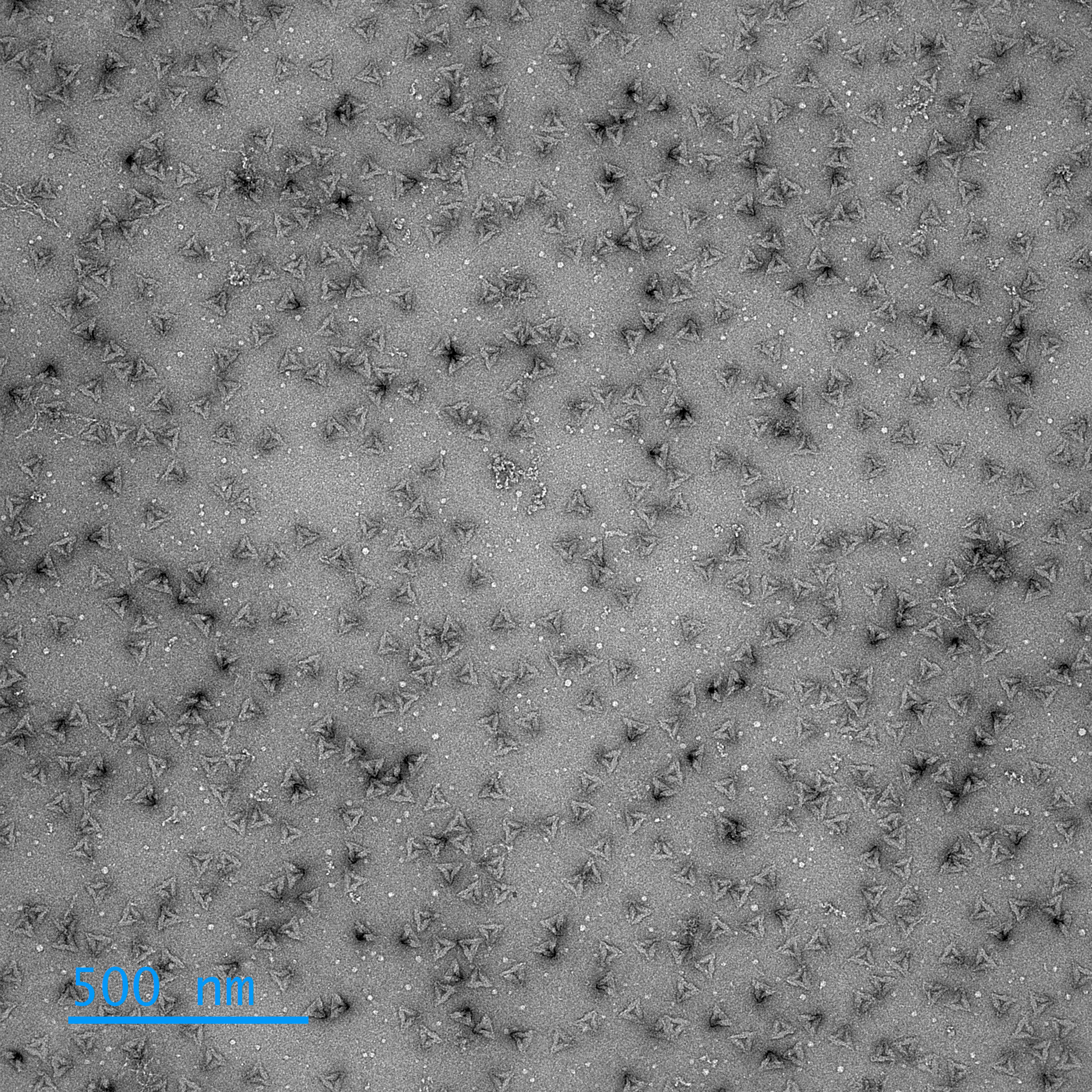

Supplement: Supplementary file 8 — Uncropped image of Figure 3A, Uncropped image of Figure 3B left, Uncropped image of Figure 3B right bottom, Uncropped image of Figure 3B right top, Uncropped image of Figure 3C left, Uncropped image of Figure 3C right, Uncropped image of Figure 3D left, Uncropped image of Figure 3D right [file 41565_2023_1468_MOESM8_ESM.zip › Source Data Figure 3/Uncropped image_Figure 3A.jpg]

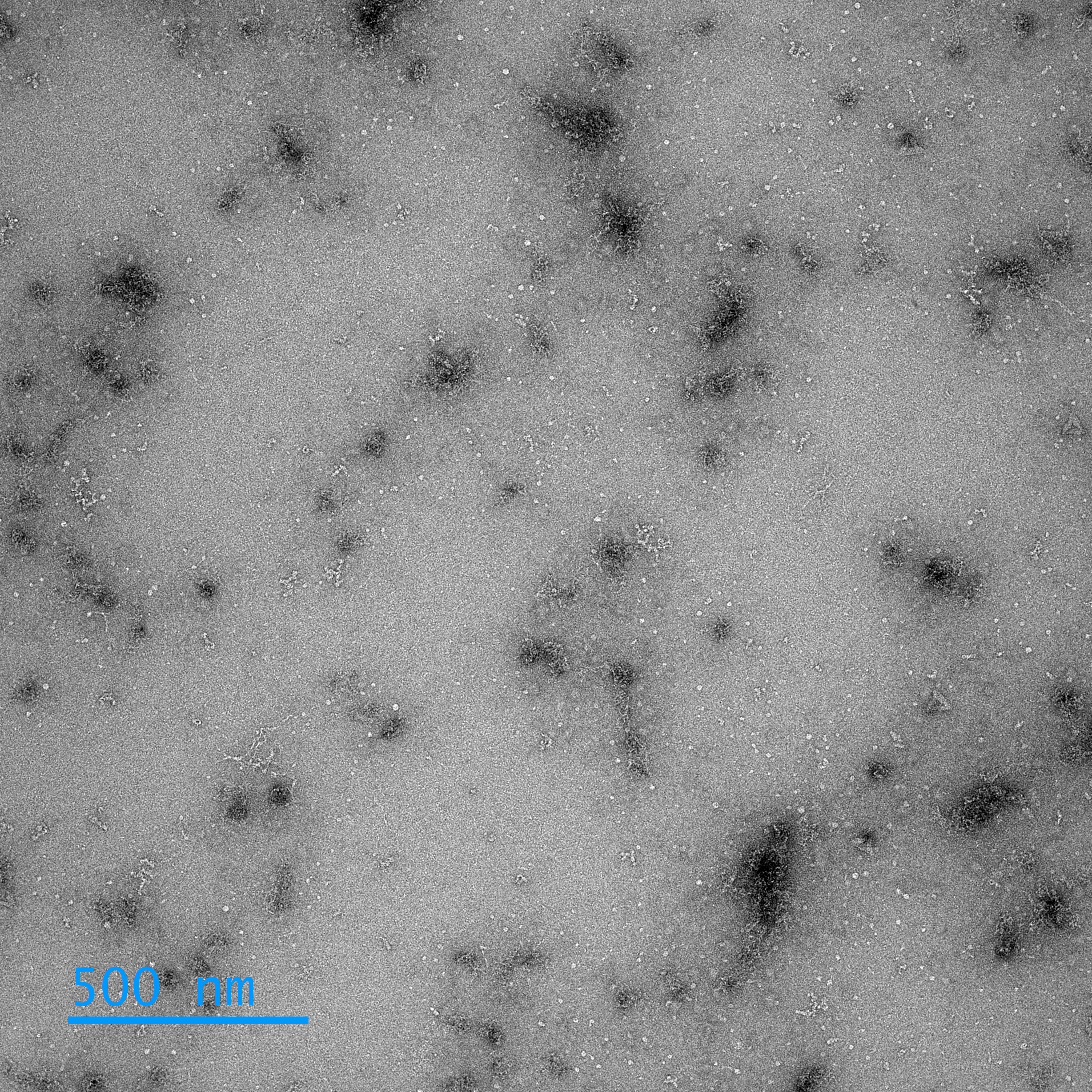

Supplement: Supplementary file 8 — Uncropped image of Figure 3A, Uncropped image of Figure 3B left, Uncropped image of Figure 3B right bottom, Uncropped image of Figure 3B right top, Uncropped image of Figure 3C left, Uncropped image of Figure 3C right, Uncropped image of Figure 3D left, Uncropped image of Figure 3D right [file 41565_2023_1468_MOESM8_ESM.zip › Source Data Figure 3/Uncropped image_Figure3B_left.jpg]

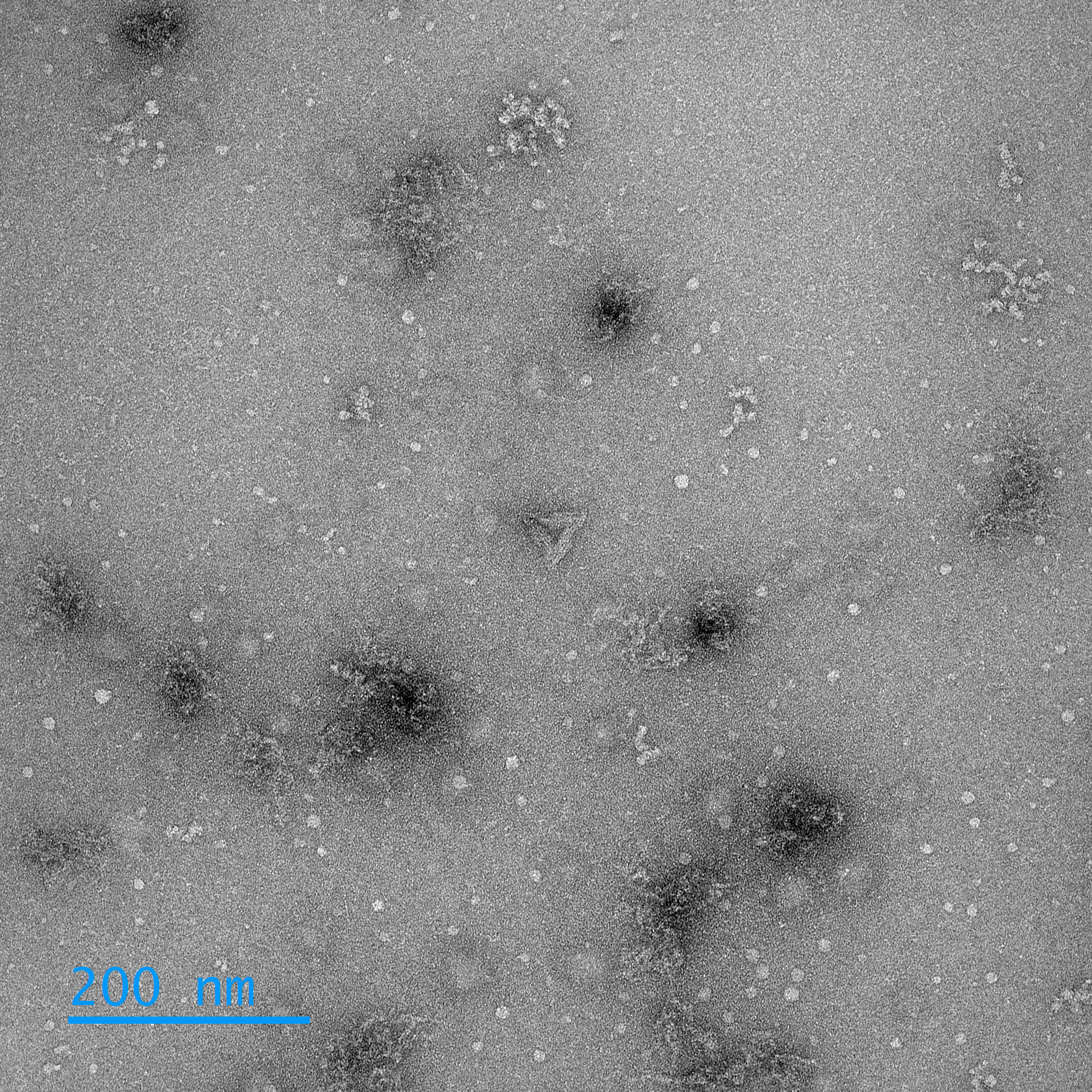

Supplement: Supplementary file 8 — Uncropped image of Figure 3A, Uncropped image of Figure 3B left, Uncropped image of Figure 3B right bottom, Uncropped image of Figure 3B right top, Uncropped image of Figure 3C left, Uncropped image of Figure 3C right, Uncropped image of Figure 3D left, Uncropped image of Figure 3D right [file 41565_2023_1468_MOESM8_ESM.zip › Source Data Figure 3/Uncropped image_Figure3B_right_bottom.jpg]

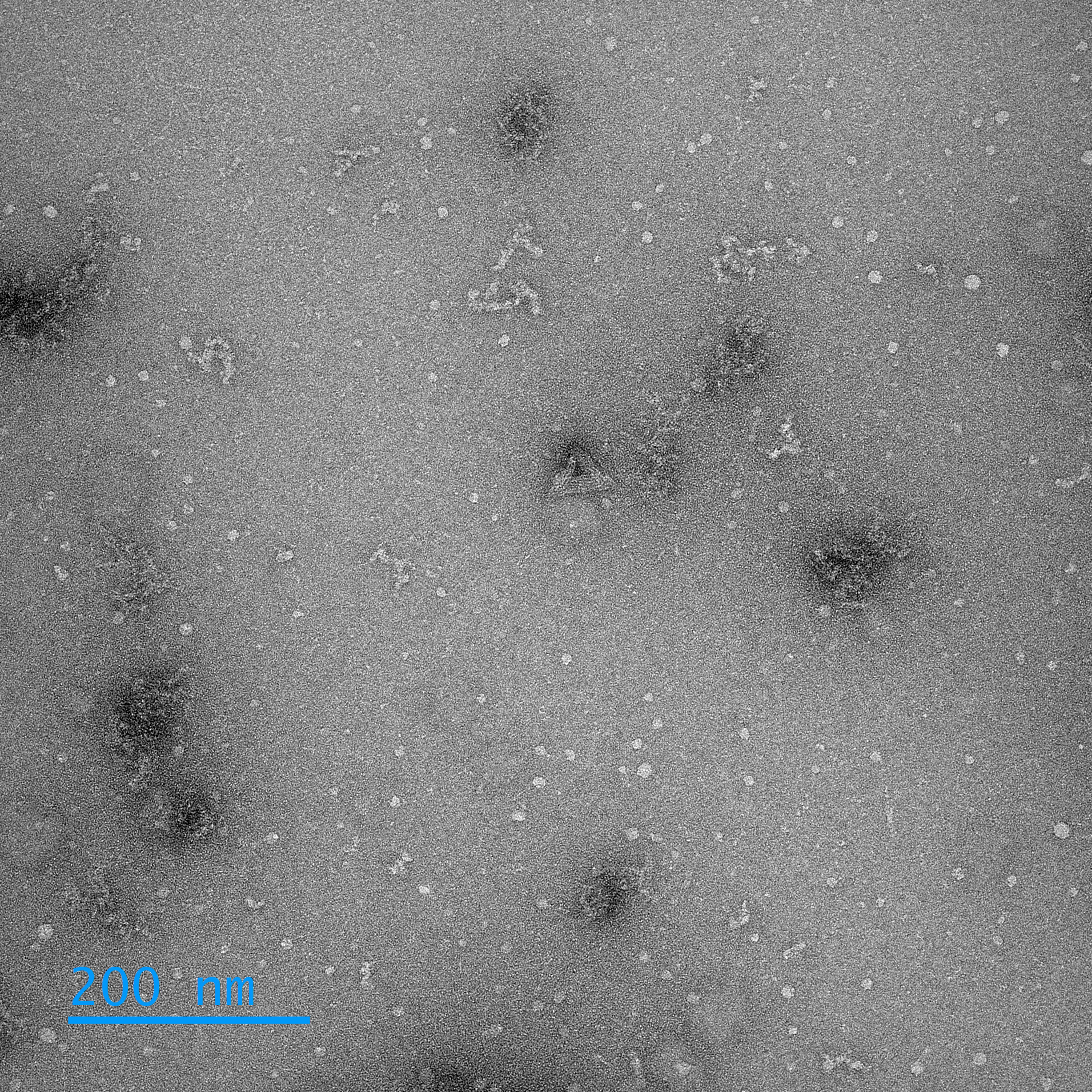

Supplement: Supplementary file 8 — Uncropped image of Figure 3A, Uncropped image of Figure 3B left, Uncropped image of Figure 3B right bottom, Uncropped image of Figure 3B right top, Uncropped image of Figure 3C left, Uncropped image of Figure 3C right, Uncropped image of Figure 3D left, Uncropped image of Figure 3D right [file 41565_2023_1468_MOESM8_ESM.zip › Source Data Figure 3/Uncropped image_Figure3B_right_top.jpg]

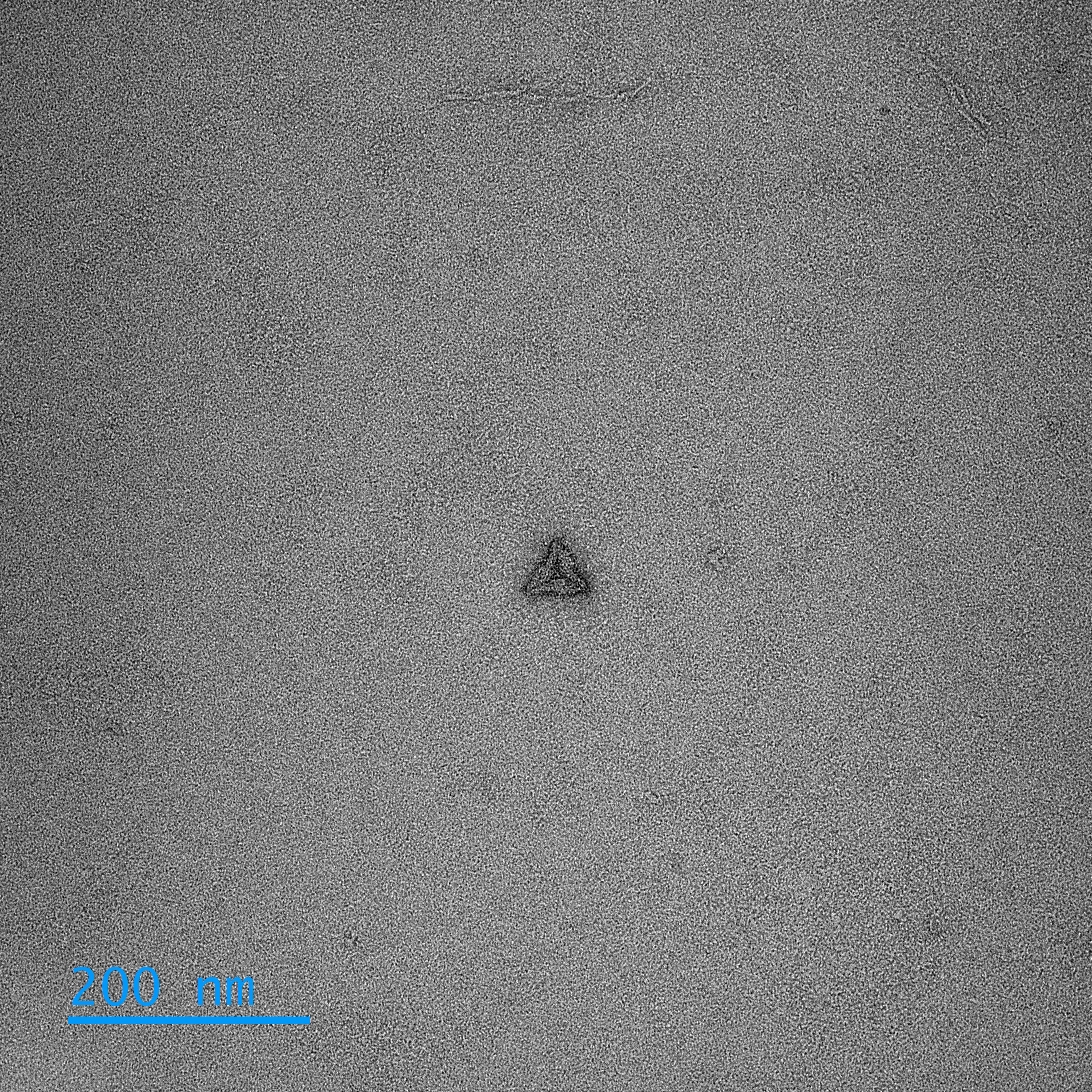

Supplement: Supplementary file 8 — Uncropped image of Figure 3A, Uncropped image of Figure 3B left, Uncropped image of Figure 3B right bottom, Uncropped image of Figure 3B right top, Uncropped image of Figure 3C left, Uncropped image of Figure 3C right, Uncropped image of Figure 3D left, Uncropped image of Figure 3D right [file 41565_2023_1468_MOESM8_ESM.zip › Source Data Figure 3/Uncropped image_Figure3C_left.jpg]

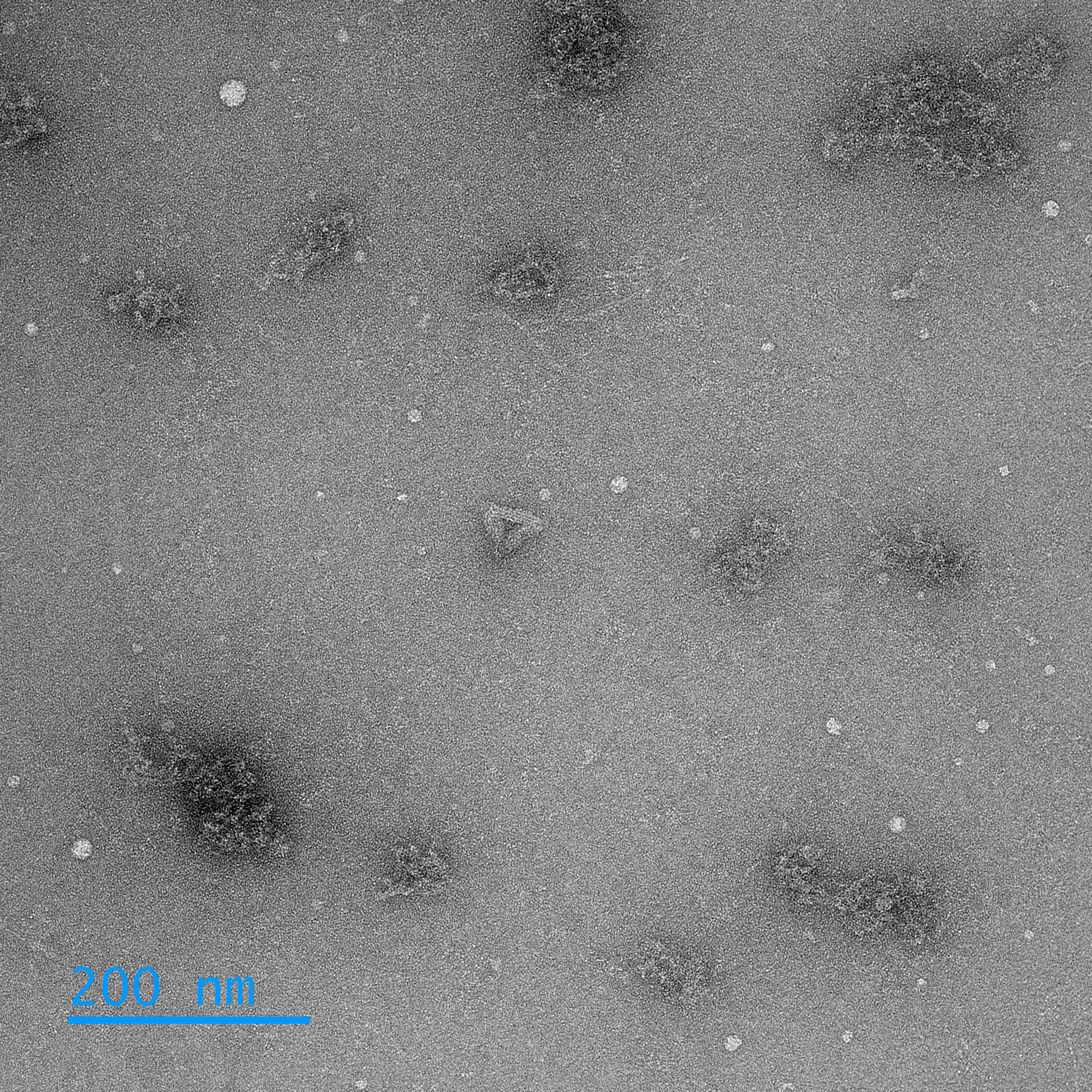

Supplement: Supplementary file 8 — Uncropped image of Figure 3A, Uncropped image of Figure 3B left, Uncropped image of Figure 3B right bottom, Uncropped image of Figure 3B right top, Uncropped image of Figure 3C left, Uncropped image of Figure 3C right, Uncropped image of Figure 3D left, Uncropped image of Figure 3D right [file 41565_2023_1468_MOESM8_ESM.zip › Source Data Figure 3/Uncropped image_Figure3C_right.jpg]

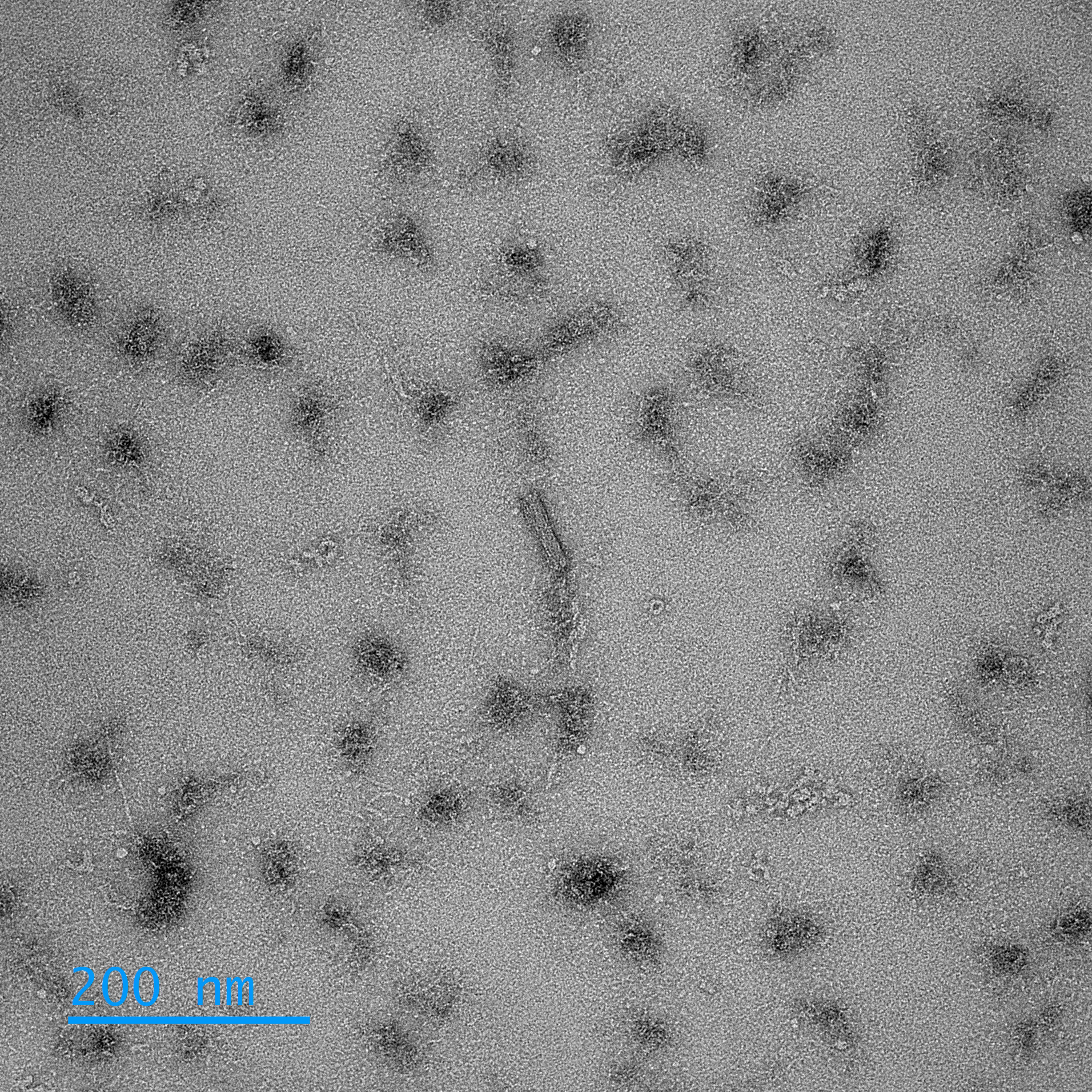

Supplement: Supplementary file 8 — Uncropped image of Figure 3A, Uncropped image of Figure 3B left, Uncropped image of Figure 3B right bottom, Uncropped image of Figure 3B right top, Uncropped image of Figure 3C left, Uncropped image of Figure 3C right, Uncropped image of Figure 3D left, Uncropped image of Figure 3D right [file 41565_2023_1468_MOESM8_ESM.zip › Source Data Figure 3/Uncropped image_Figure3D_left.jpg]

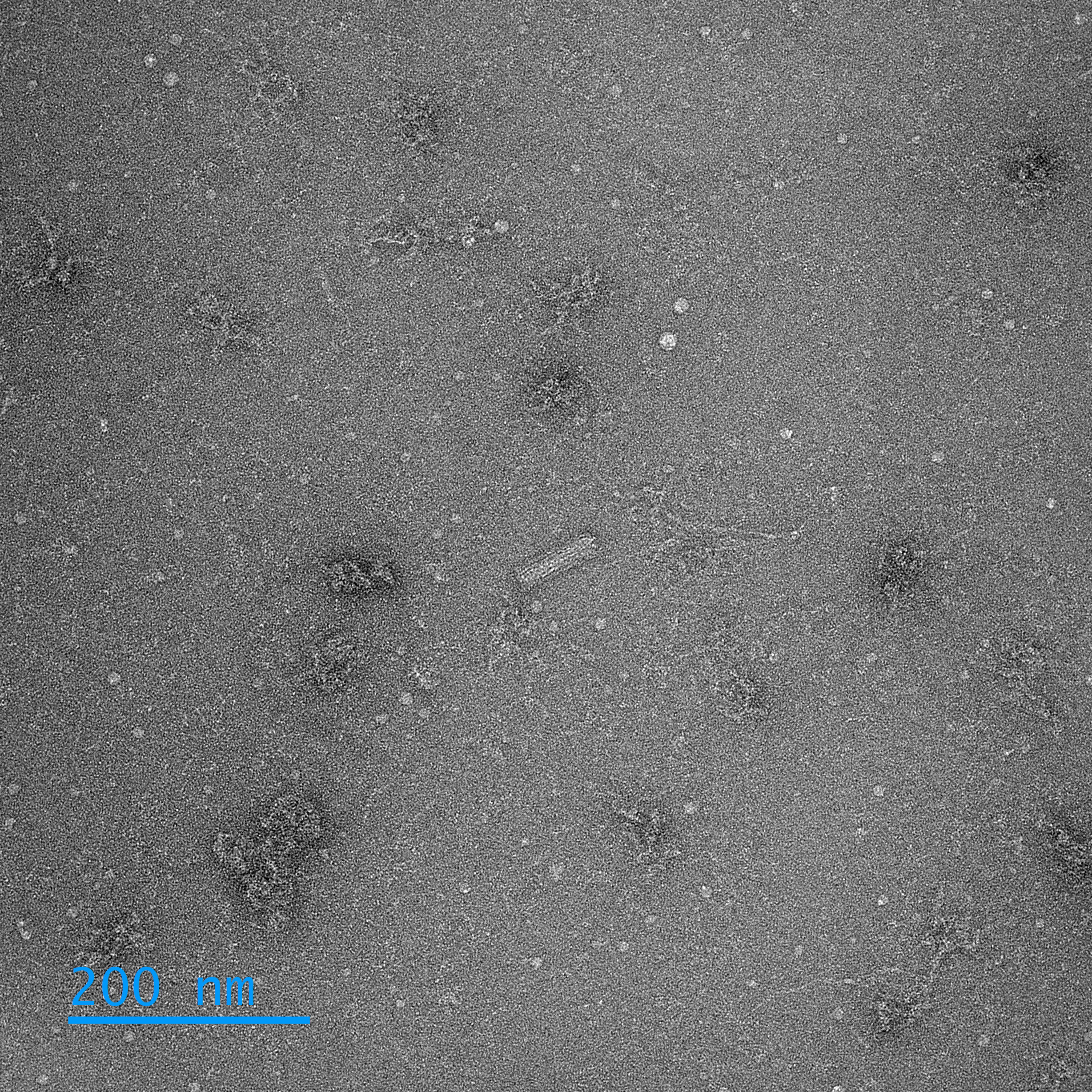

Supplement: Supplementary file 8 — Uncropped image of Figure 3A, Uncropped image of Figure 3B left, Uncropped image of Figure 3B right bottom, Uncropped image of Figure 3B right top, Uncropped image of Figure 3C left, Uncropped image of Figure 3C right, Uncropped image of Figure 3D left, Uncropped image of Figure 3D right [file 41565_2023_1468_MOESM8_ESM.zip › Source Data Figure 3/Uncropped image_Figure3D_right.jpg]
